# Global Changes in Secondary Atmospheric Pollutants During the 2020 COVID‐19 Pandemic

**DOI:** 10.1029/2020JD034213

**Published:** 2021-04-27

**Authors:** Benjamin Gaubert, Idir Bouarar, Thierno Doumbia, Yiming Liu, Trissevgeni Stavrakou, Adrien Deroubaix, Sabine Darras, Nellie Elguindi, Claire Granier, Forrest Lacey, Jean‐François Müller, Xiaoqin Shi, Simone Tilmes, Tao Wang, Guy P. Brasseur

**Affiliations:** ^1^ National Center for Atmospheric Research Atmospheric Chemistry Observations and Modeling Laboratory Boulder CO USA; ^2^ Environmental Modeling Group Max Planck Institute for Meteorology Hamburg Germany; ^3^ Laboratoire d’Aérologie Université de Toulouse CNRS UPS France; ^4^ Observatoire Midi‐Pyrénées Toulouse France; ^5^ Department of Civil and Environmental Engineering The Hong Kong Polytechnic University Hong Kong China; ^6^ Royal Belgian Institute for Space Aeronomy Brussels Belgium; ^7^ NOAA Chemical Sciences Laboratory/CIRES University of Colorado Boulder CO USA

## Abstract

We use the global Community Earth System Model to investigate the response of secondary pollutants (ozone O_3_, secondary organic aerosols SOA) in different parts of the world in response to modified emissions of primary pollutants during the COVID‐19 pandemic. We quantify the respective effects of the reductions in NOx and in volatile organic carbon (VOC) emissions, which, in most cases, affect oxidants in opposite ways. Using model simulations, we show that the level of NOx has been reduced by typically 40% in China during February 2020 and by similar amounts in many areas of Europe and North America in mid‐March to mid‐April 2020, in good agreement with space and surface observations. We show that, relative to a situation in which the emission reductions are ignored and despite the calculated increase in hydroxyl and peroxy radicals, the ozone concentration increased only in a few NOx‐saturated regions (northern China, northern Europe, and the US) during the winter months of the pandemic when the titration of this molecule by NOx was reduced. In other regions, where ozone is NOx‐controlled, the concentration of ozone decreased. SOA concentrations decrease in response to the concurrent reduction in the NOx and VOC emissions. The model also shows that atmospheric meteorological anomalies produced substantial variations in the concentrations of chemical species during the pandemic. In Europe, for example, a large fraction of the ozone increase in February 2020 was associated with meteorological anomalies, while in the North China Plain, enhanced ozone concentrations resulted primarily from reduced emissions of primary pollutants.

## Key Points

- During the COVID‐19 lockdown, the atmospheric concentration of primary pollutants (NOx, VOCs, CO, SO_2_) was considerably reduced
- The concentration of secondary pollutants increased in NOx‐saturated areas and decreased in NOx‐limited areas
- The response of the chemical system depends on the relative changes in NOx and VOC emissions, and is affected by weather variability

## 1 Introduction

With the development of the COVID‐19 pandemic and the resulting slowdown in economic activity, first in China and then in the rest of the world, anthropogenic emissions of primary pollutants were significantly altered after January 2020. This unanticipated planet‐wide experiment allows us to examine the response of the atmosphere's chemical system and in particular, the formation of secondary compounds such as ozone (O_3_) and the fraction of the airborne particles including PM_2.5_(particles with a diameter smaller than 2.5 μm) that is produced in situ. It offers a glimpse into a potential future in which air quality would be improved following structural regulations in the emissions of nitrogen oxides (NOx), carbon monoxide (CO), and volatile organic compounds (VOCs). A reduction in the emissions of the pollutants is expected to modify the level of photooxidants present in the atmosphere and the formation of secondary species including ozone (O_3_) or secondary organic aerosols (SOA) (Huang et al., 2020; Kroll et al., 2020; Miyazaki et al., 2020). In some polluted geographical areas, the large decrease observed in NOx concentrations might have shifted the chemical regimes from NOx‐saturated toward NOx‐sensitive conditions. A better understanding of the chemical processes that determine the oxidative potential of the atmosphere and their disruption during the pandemic is therefore useful in developing adequate measures to improve air quality.

The pandemic manifested itself first in China, where the first lockdown measures were adopted from the end of January to the month of March. In Europe, North and South America as well as India and the Middle East, lockdowns were imposed with varying degrees of stringency from March onwards and lasted at least until June.

Observations by spaceborne and ground‐based instruments during the first months of 2020 show a substantial decrease in the atmospheric concentrations of NO_2_ relative to measurements performed during the same period in 2019 (e.g., Bauwens et al., 2020; Le et al., 2020; Liu et al., 2020; Shi & Brasseur, 2020), relative to longer term averaged data (e.g., Deroubaix et al., 2021) or relative to model‐based weather benchmarks (Keller et al., 2020; Venter et al., 2020). Numerous specific studies analyzing air quality anomalies have focused on specific regions or urban areas (e.g., Baldasano, 2020; Bedi et al., 2020; Chauhan & Singh, 2020; Fu, Purvis‐Roberts, et al., 2020; He et al., 2020; Krecl et al., 2020; Menut et al., 2020; Otmani et al., 2020; Rodriguez‐Urrego & Rodriguez‐Urrego, 2020; Sicard et al., 2020; Siciliano, Carvalho, et al., 2020; Siciliano, Dantas, et al., 2020; Zangari et al., 2020 among many others). A large fraction of the observed reductions in air pollutant emissions has been attributed to a drastic disruption in road traffic and in manufacturing operations. In the city of Wuhan, where the pandemic started and very strict lockdown measures were imposed to the entire population, NO_2_ and PM_2.5_ concentrations were reduced by approximately 50%–60% and 30%–40%, respectively, while a large positive anomaly was reported in the concentration of surface ozone (Fu, Wang, et al., 2020; Lian et al., 2020; Shi & Brasseur, 2020). For the North China Plain (NCP), the ozone increase was estimated to be larger than 40% (Huang et al., 2020; Shi & Brasseur, 2020; Zhu et al., 2020). Compared to the first months of 2019, measurements made by the spaceborne Tropospheric Monitoring Instrument (TROPOMI) onboard the Sentinel‐5 Precursor satellite in early 2020 showed a decrease in the NO_2_ column of typically 40%–50% during the lockdown in northern China (Bauwens et al., 2020). Using TROPOMI data, Miyazaki et al. (2020) estimated a reduction of Chinese NOx emissions reaching 36% from early January to mid‐February 2020. Several studies assessing the impact of the COVID‐19 pandemic on the emissions of greenhouse gases showed for example that the emission of CO_2_ decreased by about 11%–25% in April 2020 relative to the mean 2019 levels (Forster et al., 2020; Le Quéré et al., 2020). To analyze observational data during the pandemic, all the reported numbers must be disentangled from the long‐term changes in pollutant emissions associated, for example, with air quality and climate policies, multi‐scale meteorological variability and the occurrence of occasional societal events such as the New Year festivals in China. The need to consider the influence of weather variability (i.e., anomalies in temperature, humidity, circulation, cloudiness, boundary layer stability) during the pandemic has been highlighted by Diamond and Wood (2020), Barré et al. (2020), Deroubaix et al. (2021), Liu and Wang (2020), Ordóñez et al. (2020), Wang and Zhang (2020), and several other authors. Models have the advantage that they can isolate these different effects and derive the response of the atmosphere to the specific forcing mechanisms.

In this study, we use a global earth system model with a comprehensive representation of atmospheric gas phase and aerosol chemistry to analyze the importance of the chemical and meteorological processes that have led to a change in the surface concentrations of primary pollutants (e.g., NOx, CO, VOCs, SO_2_, organic and black carbon), secondary photooxidants (e.g., ozone and radicals such OH, HO_2_, RO_2_, where R is an organic chain such as CH_3_ or C_2_H_5_) and aerosol particles in several regions of the world in response to the reduced emissions of volatile organic compounds, carbon monoxide and nitrogen oxides during the period January–May 2020. The subsequent situation, linked to the onset of a second wave of the pandemic in late 2020, is not considered in this work.

To quantify the role of different processes that affected the level of pollutants during the COVID‐19 pandemic period, different components of the atmospheric system must be carefully examined (Kroll et al., 2020):

(1) the changes in the emissions of primary pollutants resulting from the reduction in economic activities; these include primarily an abrupt disruption in road, air, and maritime traffic as well as in industrial activities, but a possible increase in domestic activities
(2) the changes in chemical regimes and specifically in the formation rate of secondary pollutants associated, for example, with a shift from VOC to NOx controlled conditions, and in the formation of ozone and secondary organic aerosols under lower NOx levels
(3) the changes in the concentration and chemical composition of particulate matter
(4) the changes in meteorological factors including temperature, humidity, dynamical variability, boundary layer physics, cloudiness, precipitation, and the related multiscale transport processes.

The purpose of the study is to assess the nonlinear relationship between the synergistic emission reduction of atmospheric primary pollutants during the COVID‐19 pandemic and the level of atmospheric photooxidants and secondary species (e.g., ozone and secondary organic aerosols) produced in different regions of the world during the early months of 2020. The results of this study should be compared with other global modeling studies including those of Weber et al. (2020), Miyazaki et al. (2020), and Li et al. (2021).

Ozone is formed during daytime by nonlinear processes at a rate that is determined by the atmospheric concentrations of VOCs and NOx. Under low NOx levels in remote or weakly polluted areas, the ozone production is controlled (or limited) by the concentration of NOx. In this case, NOx regulates the rate of the RO_2_ + NO reaction, which controls radical propagation. Ozone is primarily destroyed by reactions involving hydrogenated species leading eventually to the formation of hydrogen peroxide (HO_2_ + HO_2_ → H_2_O_2_), which is scavenged by wet and dry deposition. In very high NOx environments, that is, in heavily polluted areas including industrial and urban complexes, nitrogen oxides act as a sink for the OH radical, which slows down the oxidation of VOCs and hence the formation of peroxy radicals. As a result, the ozone production is considerably reduced. Rather, ozone is sequestered by NO to form NO_2_, which is eventually converted to nitric acid (NO_2_ + OH → HNO_3_) and removed from the atmosphere by wet and dry scavenging. This situation is referred to as NOx‐saturated or VOC‐controlled conditions. The reduction in VOCs and NOx during the pandemic is, therefore, expected to have led to a reduction of ozone in NOx‐limited regions, but to have caused an increase in the ozone concentration in the most polluted areas, especially during winter when the levels of NOx are the highest. An increased oxidation capacity in the eastern part of China has been reported in Huang et al. (2020), whereas enhanced concentrations of ozone in the North China Plain were reported by Liu and Wang (2020), Shi and Brasseur (2020), and Miyazaki et al. (2020). This question will be further examined in subsequent sections.

The dominant source of secondary organic aerosols is provided by the oxidation of biogenic hydrocarbons including isoprene and terpenes, and of anthropogenic VOCs (linear and aromatic hydrocarbons) resulting from fossil fuel consumption, the industrial and domestic use of solvents and of other products, and from biomass burning. The rate at which the degradation of primary hydrocarbons proceeds, depends on the concentration of oxidants and on the level of nitrogen oxides (NOx) present in the atmosphere (Hallquist et al., 2009). A reduction in VOCs tends to reduce the formation rate of SOA, while a reduction in NOx tends to increase the SOA production (Ng et al., 2007) under high NOx conditions.

The study is organized as follows. Section 2 provides a short description of the emission reductions that are considered here as a forcing factor to the anomalies in the concentrations of chemical species during the pandemic period. Section 3 presents a brief description of the global earth system model that is adopted to analyze the atmospheric response to this forcing during the pandemic. More details are found in the supplementary information. Section 4 provides a global view of the changes that have occurred in the chemical composition of the atmosphere in response to the reduced emissions of primary pollutants and to meteorological anomalies occurring during the pandemic. Section 5 assesses the calculated changes in the concentration of chemical species in selected regions (China, Europe, North and South America), where lockdowns were imposed during the first months of 2020. This section discusses in particular the respective impact of the reduction in NOx versus VOC emissions as well as the role of meteorological variability on the calculated chemical fields. A summary of the findings and key conclusions are provided in Section 6.

## 2 Adjustments in Emissions During the Pandemic

**Figure 1:**
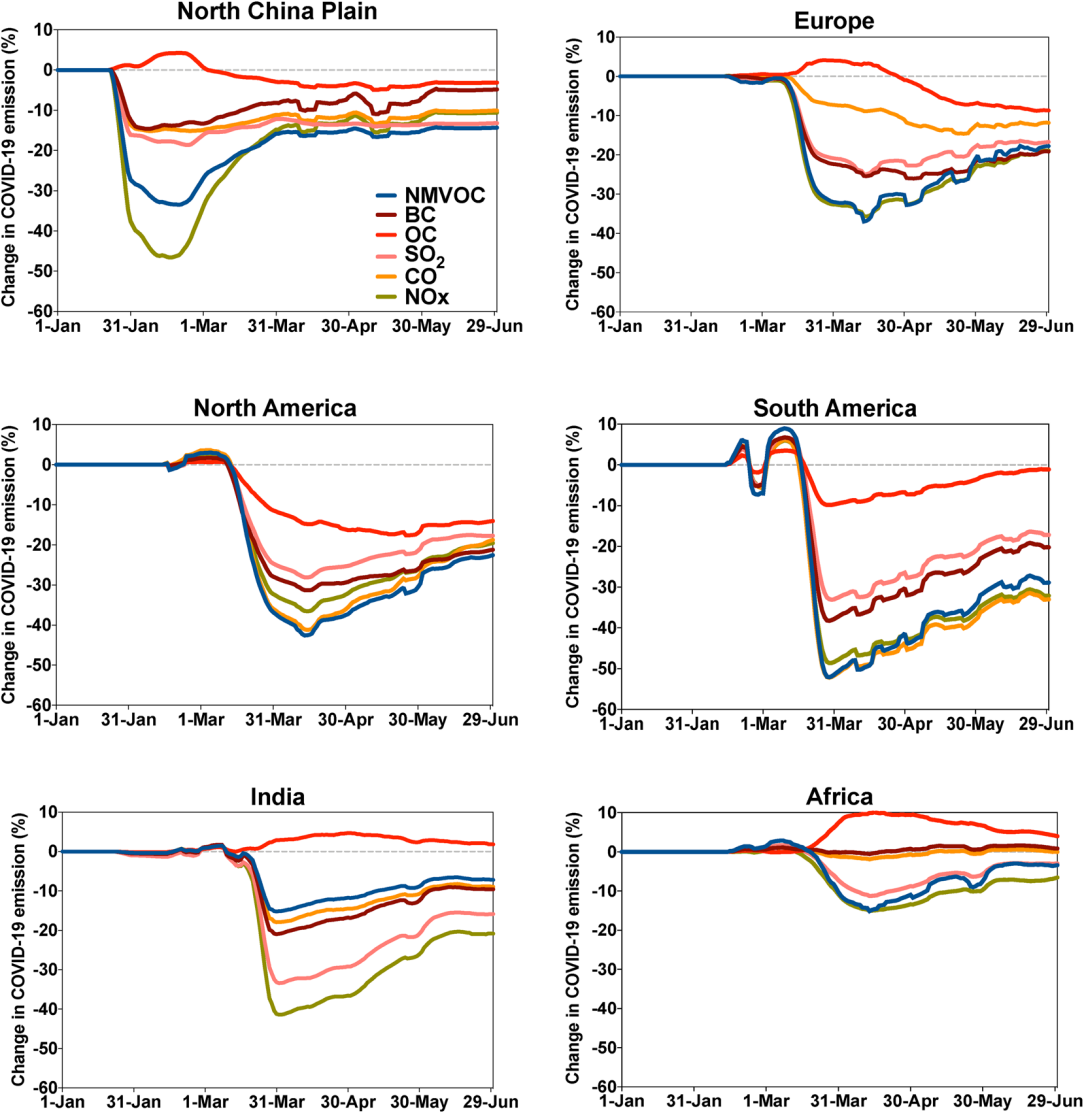
Evolution from January 1 to June 30, 2020 of the daily geographically averaged adjustment factors (percent) for the emissions for nitrogen oxides (NOx), carbon monoxide (CO), sulfur dioxide (SO_2_), organic carbon (OC), black carbon (BC), and nonmethane volatile organic carbon (NMVOC) in the North China Plain, Europe, North America, South America, India, and Africa. A weak filter has been applied to smooth out he high frequency variability in the curves. Based on Doumbia et al. (2021).

The change in the emissions of primary pollutants associated with the COVID‐19 pandemic has considerably varied among different economic sectors and geographic areas. The time at which the lockdowns were enforced and the severity of the measures taken to protect the population were different from country to country and even from region to region. Several studies (Doumbia et al., 2021; Guevara et al., 2020) have attempted to estimate these changes in emissions on the basis of available economic information regarding different sectors: transportation (road, air, and sea traffic), industrial production, energy consumption, and residential activity. Here, we adopt the global estimates provided by Doumbia et al. (2021) gridded at a spatial resolution of 0.1 × 0.1° (about 10 × 10 km). In this study, adjustment factors (applied to the baseline emissions without COVID‐19 effects) were derived for each economic sector and geographic region based on activity data, and the resulting changes in the emissions of a given primary chemical species were calculated at each grid point of the model based on the relative contribution of each sector to the total emission. In some cases, the input data used to derive the adjustment factors was available at the country level, but in some cases, more resolved subregional‐scale and even local‐scale information was used. Some input data, for example, the reduction of road traffic intensity, was accessible on a day‐to‐day basis for major cities in many (but not all) countries, and allowed Doumbia et al. (2021) to provide 10 km resolution emission estimates on a daily basis from January–August 2020. The reduction in the emissions for the shipping and aviation sectors adopted in the present study is also obtained from Doumbia et al. (2021). Figure 1 shows the geographically averaged percentage surface emission adjustment applied during the pandemic in different regions of the world and for different chemical species. In the Northern China Plain, the change in the emissions attributed to the COVID‐19 pandemic occurred as early as January 23, 2020 and, as the lockdown was immediately implemented, happened abruptly. The largest reduction in emissions occurred in mid‐February 2020. At that time, the reduction in NOx reached 50% and is explained in large part by the nearly complete shutdown of road traffic. The second largest reduction factor, as estimated by Doumbia et al. (2021), is the decrease of 30% for the emissions of nonmethane volatile organic compounds (NMVOC). For carbon monoxide (CO), black carbon (BC), and sulfur dioxide (SO_2_), the maximum adjustment factors were close to 0.9 (10% reduction). In the case of organic carbon particulates (OC), an increase of a few percent resulted from the enhanced domestic activity (stay‐at‐home policy), particularly related to more extensive cooking and heating during the pandemic period. In the other regions of the world, the COVID‐related perturbations in the emissions was most pronounced in mid‐March to mid‐April at a time where China’s emissions were already in a recovery phase. In the European Union and North America, the estimated change in the emissions was largest in April. In South America and India, a sharp decrease appeared in the second half of March followed by a slow recovery from April to June. In Africa, only a small reduction occurred for NOx and VOCs with a maximum reduction in mid‐April and a slow recovery afterward. In all countries, except the America, a slight increase in organic carbon was derived during the pandemic.

Figure S1 in the supplementary information shows a global view of the monthly mean reduction in the emissions of NOx CO and SO_2_ for January–May 2020. In February, the reduction in the emissions is contained in China. In March and April with lockdown measures imposed in other parts of the world, substantial reductions are seen in the NOx and VOC emissions of Europe, the Middle East, India, and North America. In the Southern Hemisphere, emissions are substantially reduced on both coasts of South America and South Africa. The decrease over the global ocean accounts for the reduced shipping activity in response to the slowdown of the economy.

We should acknowledge here that large uncertainties might reside the aforementioned adjustment factors as discussed in Doumbia et al. (2021). As an example, Guevara et al. (2020) estimated for the period March 23–April 26, 2020 in Europe an average emission reduction of 33% for NOx, 8% for VOCs, and 15% for CO. The average reductions by Doumbia et al. (2021) for the similar period are 33% for NOx, 30% for VOCs, and 15% for CO. The difference in the VOC emission adjustment factors might be due to a different treatment of the reduction in solvent emissions during the pandemic, as solvents contribute a large share of the anthropogenic VOC emissions in Europe. In the model simulations presented in the following sections, we address this particular uncertainty by considering a case in which the high VOC emission reduction of Doumbia et al. (2021) is adopted (upper limit) and a case in which no reduction in VOC and CO emissions is applied (lower limit).

## 3 Global Model Description

The distribution of chemical species presented in our study are derived from the Community Earth System Model (CESM) version 2.2 (Danabasoglu et al., 2020). The atmospheric component of CESM, the Community Atmosphere Model (CAM‐Chem) (Emmons et al., 2020; Gaubert et al., 2020; Gettelman et al., 2019; Tilmes et al., 2020) that is described in the supplementary information, provides a comprehensive description of atmospheric chemistry and aerosol processes with 221 gas phase and aerosol species, and 528 chemical and photochemical reactions (Emmons et al., 2020). Aerosols are represented by the four‐mode Modal Aerosol Model (MAM4, Liu et al., 2016; Mills et al., 2016). To closely track actual meteorological conditions, the wind velocity components and the temperature are nudged toward the Modern‐Era Retrospective Analysis for Research and Applications version 2 (MERRA‐2, Gelaro et al., 2017). Adopted biogenic, pyrogenic, and anthropogenic emissions are described as part of the supplementary information. Baseline emissions obtained from an interpolation of the monthly averaged emissions are adjusted for COVID‐related runs by applying the daily adjustment factors discussed in Section 2 (Doumbia et al., 2021).

To evaluate the relative influence of different forcing processes responsible for the variations in the surface concentrations of reactive species, we perform different model simulations that are summarized in Table 1. In case 1, referred to as the “control” case, the anthropogenic and pyrogenic emissions and the meteorology nudged to the MERRA‐2 analysis correspond to year 2020; the effect of COVID‐19 is ignored. Case 2 refers to a simulation performed for the period 2001–2015 with annually repeated anthropogenic and pyrogenic emissions and with nudged meteorology (evolving from year to year). A climatology (called “Climato”) is derived from this simulation and is used as a reference to isolate the anomalies caused by the particular meteorological situation of 2020. Case 3 (called COVID‐All) is similar to case 1, but with an adjustment in all the emissions during the COVID‐19 pandemic period. Cases 4 and 5 are similar, but with adjustments for only NOx (called COVID‐NOx) and for only VOCs and CO (called COVID‐VOC), respectively. Global distributions of simulated chemical species obtained with the control case are displayed in Figure S2 of the supplementary information. Information about the performance of the model is provided in Figure S3 and Figure S4a–S4h.

**Table 1:** Description of the Different Model Simulations Used in the Present Study

Case	Name	Reduction in emissions	Meteorology	Notes
1	Control (2020)	None	MERRA‐2 for 2020	Control with business‐as‐usual 2020 emissions (no COVID effect)
2	Climato (2015–2019)	None	MERRA‐2 evolving from 2001 to 2019	Repeated emissions
3	COVID‐All (2020)	All emitted species	MERRA‐2 for 2020	Combined emission adjustment for COVID effects
4	COVID‐NOx (2020)	NOx only	MERRA‐2 for 2020	Impact of NOx only adjustment
5	COVID‐VOC (2020)	VOCs and CO only	MERRA‐2 for 2020	Impact of VOC and CO reduction only adjustment

## 4 Changes in Surface Air Quality during the COVID‐19 Pandemic

We first examine the global response of the chemical composition to the changes in the surface emissions during the pandemic. In Section 4.1, we compare the model results obtained with and without the modified emissions (COVID‐all minus Control) as described above. In order to isolate the impact of the changes in emissions, we constrain the model in both cases by the same meteorological input corresponding to the year 2020. In Section 4.2, we show how the particular dynamical situation in 2020 has produced anomalies relative to a multiyear averaged meteorology. We present panels that provide the percentage change in the monthly mean surface concentration of key chemical species for two different months: February and April, which correspond to the peak time of the lockdown episode in China and in the rest of the world, respectively. Model results for the period January–June 2020 are provided as supplementary information (Figure S5). Note that the patterns presenting relative changes may be very different from patterns of absolute changes. The seasonal evolution of the ozone response to the COVID‐19 perturbation in the free troposphere as derived by the CESM model are shown by Bouarar et al. (2021). Analyses of observed anomalies in the surface concentrations of primary and secondary air pollutants (NOx, ozone) in different regions of the world are reported by Tang et al. (2020). These authors provide information that is useful for the validation of the model results presented here. In this Section, we present results at the global scale. A more detailed analysis of the chemical processes that are responsible for the calculated changes in selected regions of the world is provided in Section 5.

### 4.1 Response to Changes in Surface Emissions of Primary Pollutants

**Figure 2:**
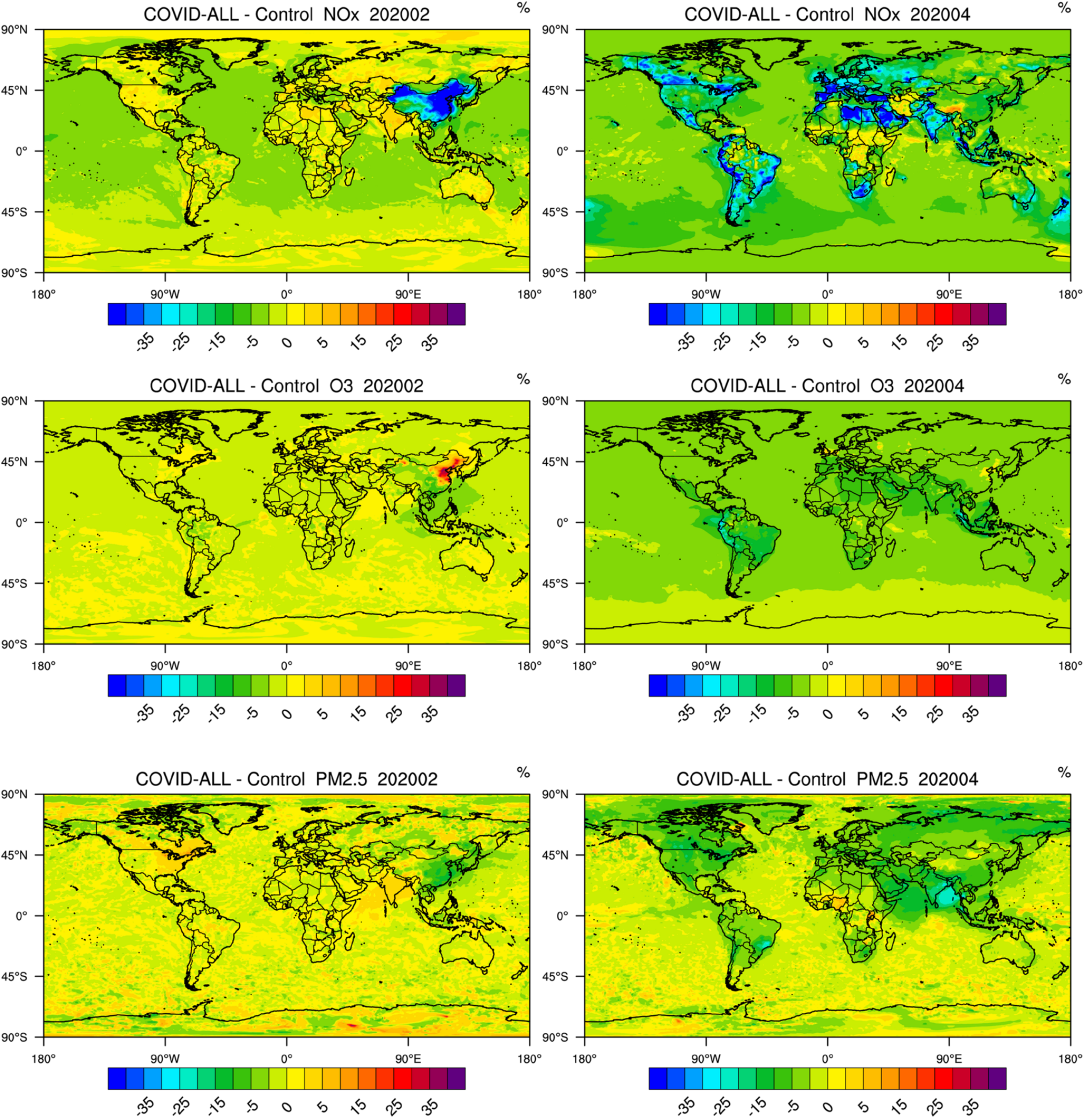
Relative change (percent) in February and April 2020 in the global monthly mean concentration of (from top to bottom) of NOx, ozone, and PM_2.5_ resulting from the change in the adopted surface emissions of primary pollutants during the COVID‐19 pandemic period.

When examining the changes in the surface abundance of nitrogen oxides resulting from the synergetic emission reduction of NOx and VOCs (Figure 2), we note a reduction in the concentration during February that amounts to 30%–50% in China, particularly in the Northern China Plain (i.e., north of the Yangtze River) and in the western province of Xinjiang. No significant reduction is yet detected in other parts of the world. Only a small decrease of a few percent is found over the oceans, particularly along the ship tracks and accounts for the assumed slowdown in international shipping activities. In April, the calculated NOx reduction in China is a factor of three smaller than two months earlier, but the impact of the pandemic has now reached most regions of the world. Reductions of typically 25%–40% are derived by the model in India, Western Europe, Saudi Arabia, Canada, and in the Southern Hemisphere, South Africa, Bolivia, Peru, and Ecuador. In Eastern Europe, New Zealand, the east coast of Australia, most regions of the United States, and Brazil, the surface concentration of NOx is reduced by 20%–30%. Very small changes are calculated for Central Africa, the center and western coast of Australia, the Asian regions of Russia and Iran. The absence of reduction calculated for Iran is consistent with the observations derived by the TROPOMI instrument (Bauwens et al., 2020).

**Figure 3:**
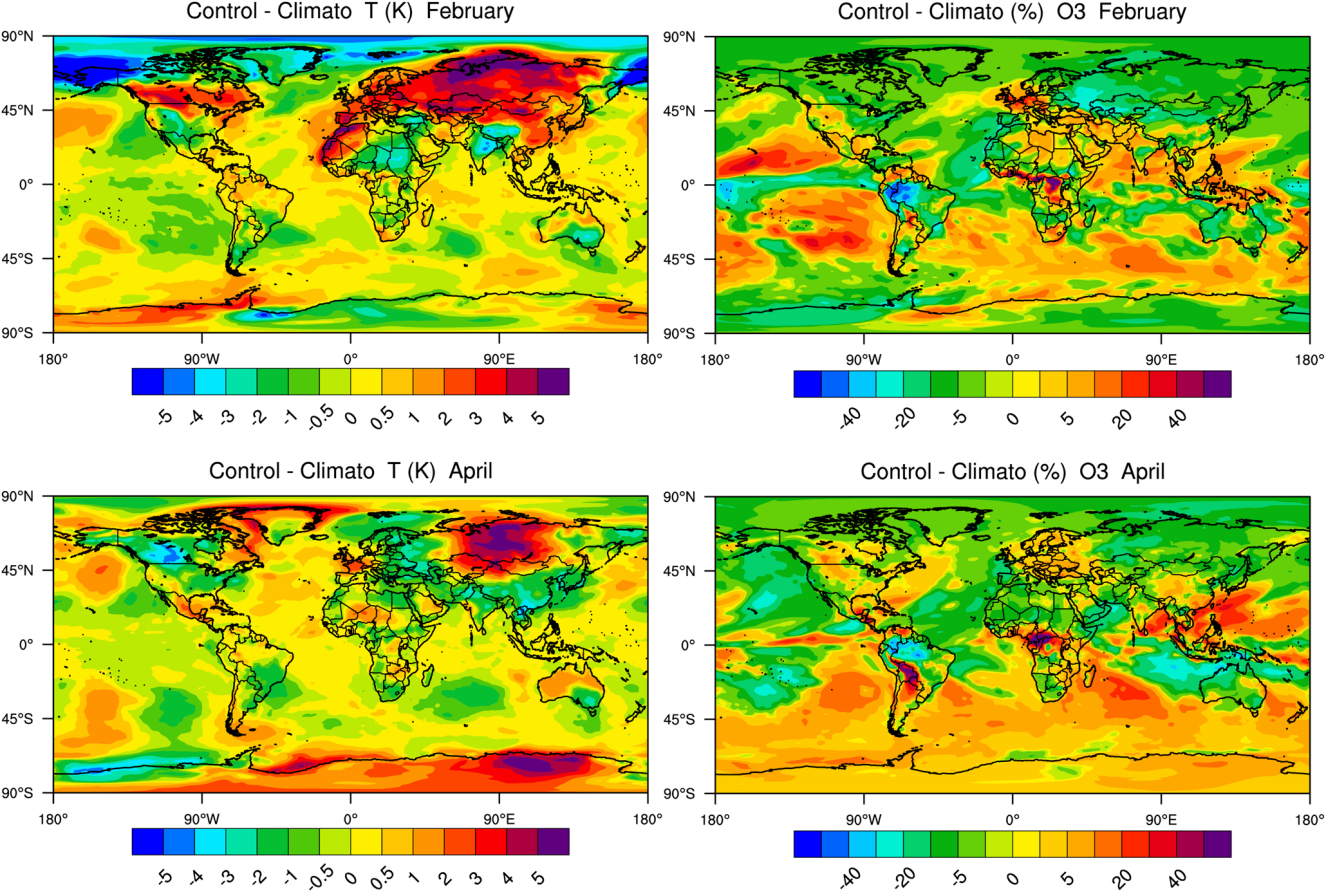
Anomalies in monthly mean surface temperature (K) and ozone concentration (percent) in 2020 relative to a 5‐year monthly mean (2015–2019) climatology highlighting the perturbation effects of the meteorological situation during the year of the pandemic (2020).

The response of ozone during the lockdown of February is characterized by a concentration increase of typically 20%–40% in the northeastern part of China. A small spot with a similar increase is found in the province of Xinjiang. In April, the ozone increase in China is vanishing, but changes in the concentration of this gas have now spread in other regions of the world. The largest relative surface ozone reductions are found in the tropics (5%–20%), specifically in northern Peru and Ecuador as well as along the Indian coasts, in Indonesia, and in Malaysia. Some increases are noted in a few regions including the urbanized regions of northern Europe, eastern Canada (Québec), and northeastern United States (East Coast, Chicago). Such specific situations will be further discussed in Section 5.

The changes in the monthly mean surface concentration of the hydroxyl radical (OH) (Figure S6), which provides indications about the change in the oxidation potential of the atmosphere, is characterized during the month of February by an increase to typically 30%–40% in northeastern and northwestern China. At the same time, the model highlights a decrease in the southern and southwestern regions of the country. During the month of March and April, an increase of OH concentration has become apparent in Northern Europe. The level of OH, however, decreases in the southern part of Europe. In the populated regions of Canada and the northeastern United States, OH concentration anomalies are positive. In the Southern Hemisphere, the OH concentration usually decreases, except in urban areas such as Santiago, Buenos Aires, Sydney, and Melbourne, where positive anomalies are derived. The level of OH is also reduced during the pandemic, along the ship tracks, as maritime traffic is limited and the related NOx emissions are smaller than under unperturbed situations.

Formaldehyde, which is directly emitted from combustion and industry, is also produced as an intermediate species in the photooxidation of primary hydrocarbons, a process that is influenced by the presence of nitrogen oxides. For this oxygenated VOC, we note a reduction in the surface H_2_CO concentration of 10%–30% in China during February 2020 (Figure S6). In April, reductions of the same order of magnitude are found in Canada, southern Europe, South Africa, as well as along the Pacific and Atlantic coasts of South America.

When considering the changes in particulate matter as calculated by the model, we note (Figure 2) that the concentration of PM_2.5_ first decreased by nearly 20% in China during the month of February. Later in April, it was reduced by 10%–25% in India, the US, and Canada and by about 10% in Europe. A fraction of this reduction is attributed to the decrease of the direct emission of particulate matter during the pandemic. However, the change in the emission of gas‐phase precursors and in their photooxidation processes under reduced NOx concentrations must also be considered. We address this issue by examining the changes affecting the quantity of SOA as derived by the model, based on the oxidation scheme described by Tilmes et al. (2020). Figure S7 shows that, according to the model (COVID‐All cases), the SOA concentration was substantially reduced, during February 2020 in China (20%–30%) and later during April in other parts of the world including the eastern US and a large area of South America. A fingerprint of the reduced SOA concentration extends in a plume over the northern Pacific Ocean. Interestingly, if only the NOx emissions had been reduced (Case 4 or COVID‐NOx), the concentration of SOA would have increased in northern part of China (20% in February and up to 10% in April) as well as in the region surrounding the English Channel (April), where the oxidation capacity increased after the pandemic outbreak. A smaller increase in SOA is seen in India, eastern Brazil, and the eastern US. If only the VOC and CO emissions had been reduced (Case 5 or COVID‐VOC), the SOA concentration would have decreased everywhere in April. The patterns of the SOA in response to the combined decrease in NOx and VOCs/CO emissions is similar to the patterns derived for the COVID‐VOC case, but with smaller concentration reductions in high NOx regions such as northern China during wintertime.

### 4.2 Effect of Meteorological Anomalies

The analysis of observed changes in the chemical composition during the pandemic requires to carefully assess the influence of meteorological variability, and when examining monthly mean values of weather anomalies for the month under consideration. The early months of 2020 were strongly affected by weather events, for example, by the passage of two storms (Ciara and Dennis) in northern Europe during the month of February and the influence of two other storms (Karine and Myriam) in southern Europe (Barré et al., 2020; Petetin et al., 2020). We assess to what extent meteorological variability during the pandemic has generated variations in the calculated chemical fields. This information should help in the analysis of observed chemical species that are affected by both the COVID‐related changes in the emissions and by weather anomalies (combined atmospheric dynamics, temperature, cloudiness, precipitation, atmospheric stability, etc.). For this purpose, we derive in Figure 3 the difference in the surface temperature and monthly mean ozone concentration in February and April 2020 relative to a 5‐year climatology derived from a model simulation conducted for the period 2015–2019. In this last case (referred to as the Climato‐case or Case 2), the surface emissions are subject to their usual seasonal variations, but their values are repeated from one year to the other.

In February 2020, besides variations occurring over the oceans, we note a small impact of the mesoscale weather situation on the monthly mean ozone fields in China. A positive ozone anomaly of 5%–10%, however, is seen along a line that stretches from northern India to Europe. This anomaly reaches about 10%–15% in northern Europe including the north of France, the Benelux countries, the UK, and Germany. In Spain, the ozone anomaly is negative (−10%–−20%). A negative anomaly of up to 20% is derived in northern China, Mongolia, and Russia. In the US, a positive anomaly of a few percent is seen in the vicinity of Chicago and along the Rocky Mountains, while there is a small negative anomaly in the eastern part of the country. In South America, the largest ozone anomaly is found along the Andes in Peru and western Brazil.

In April, the patterns of variations relative to our 5‐year climatology are characterized as follows: positive anomalies in southern China (5%–10%) and in South Asia (20%–30%), in northern and eastern Europe (5%–10%), as well along the Rockies in the US and Canada (10%–20%) and along the Andes in Argentina, Bolivia, Peru, Ecuador, and Colombia (20%–30%), negative anomaly in Russia (10%–20%), in Spain and the southwest of France (5%–15%).

### 4.3 Combined Chemical and Meteorological Effects

**Figure 4:**
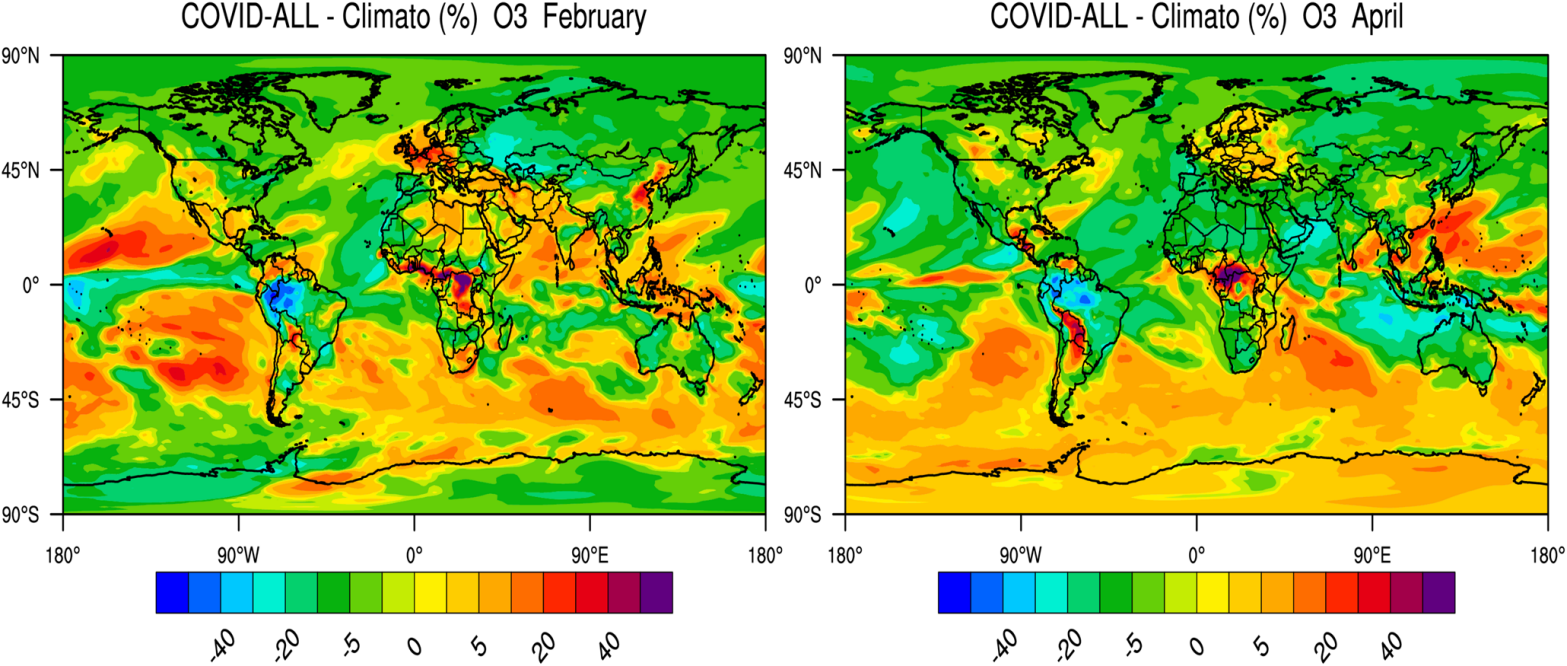
Relative change (percent) in February and April 2020 in the global monthly mean concentration of ozone resulting from the combined changes in surface emissions of primary pollutants during the COVID‐19 pandemic period and the meteorological anomalies during the same period.

Finally, we show in Figure 4 the response in February and April 2020 of the surface ozone concentration to the combined effects of the entire COVID‐related emission adjustments and of the meteorological anomalies. The purpose is to reproduce as closely as possible the real changes in ozone relative to the monthly mean values averaged over 5 years (2015–2019) without accounting for long‐term trends in emissions (COVID‐All minus Climato cases). In February, the model produces positive anomalies for ozone in northern China, northwestern Europe, in the western part of the US, in the region of the Great Lakes, and in the Middle‐East. In April, ozone is higher than the climatological values in northern and eastern Europe, in southern China, along the northern Rockies near the US‐Canadian border, east of the Andes in South America, and in the western Pacific.

We summarize the results of our model simulations (Table 2) by providing values (orders of magnitude) that characterize the ozone changes in different populated regions of the world during the middle of the pandemic (monthly mean values for February in China and for April in the rest of the world). We compare the relative importance of the contributions of chemistry (reduced emissions) and meteorological anomalies in 2020.

**Table 2:** Relative Changes (Orders of Magnitude in Percent) in the Monthly Mean Values of the Surface Concentrations of ozone as Calculated for Different Regions (Non‐urban conditions)

Region	Emission Adaptation	Meteorological anomaly
North China Plain	0–+30	0–+5
Southern China	−10–−5	−5–+5
India	−15–−5	−5–+5
Northern Europe (UK, Benelux, Germany, northern France)	+2–+5	+2–+5
Southwestern Europe (south of France, Spain)	−10–−5	−20–−5
Northeastern US and southern Canada	+2–+5	−5–+2
Eastern Brazil	−25–−10	−5–+15
Peru Ecuador	−35–−25	+5–+25
South Africa	−10–−5	−5–+2

Changes due to modified emissions during the pandemic, to specific meteorological anomalies of 2020 (relative to the average from a climatology of years 2015–2019) and to the combined effects.

## 5 Process Analysis and Discussion

To identify the chemical processes that explain the changes in the concentrations of secondary pollutants (e.g., ozone, SOA), we now examine in more detail than in Section 3, the response of a set of chemical species, which contribute to the formation and destruction of these secondary pollutants. We focus on several regions of the world, which are differentiated by the intensity of incident solar radiation and by environmental conditions such as, for example, the level of nitrogen oxides in the boundary layer. We take advantage of the fact that the season that corresponds to the lockdowns was different in different regions of the world. To quantify the respective role of nitrogen oxides and carbon compounds, we consider in addition to the simulations (COVID‐All) considered in Section 3 two additional cases: in one of them (COVID‐NOx), only the reduction of nitrogen oxide emissions is taken into account, while in the second case (COVID‐VOC), only VOC and CO emissions are reduced following Doumbia et al. (2021).

### 5.1 Air Quality in China During the Pandemic

**Figure 5:**
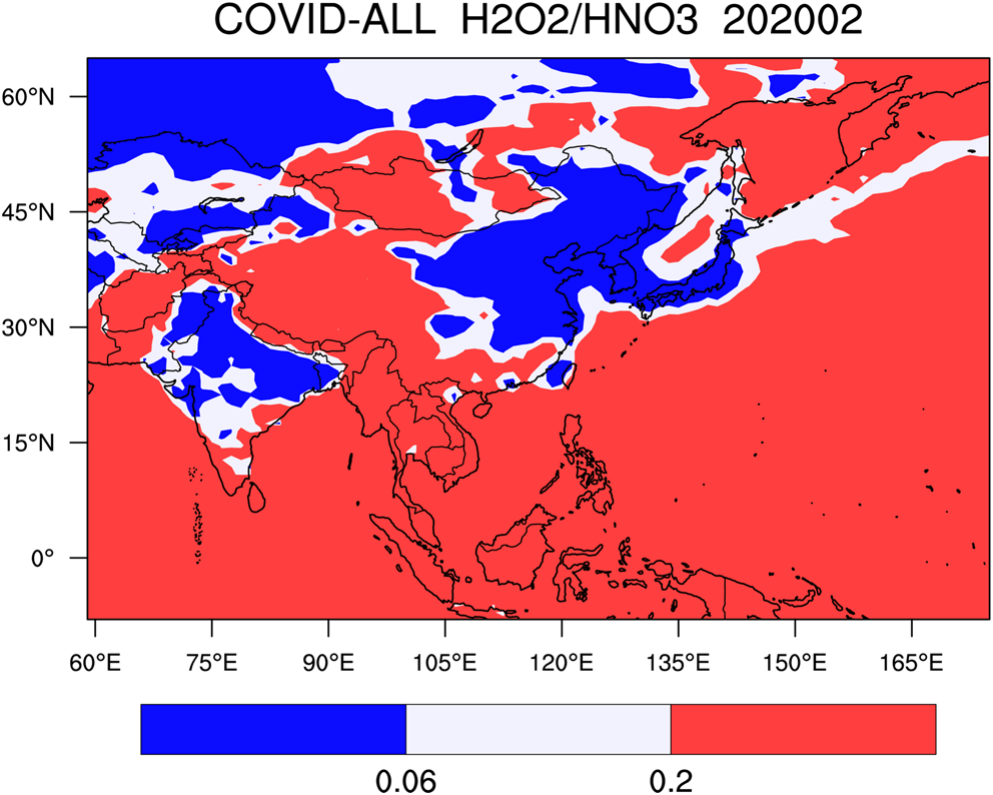
Ratio between the monthly mean production rate of hydrogen peroxide and nitric acid, a measure of the chemical conditions governing the formation of ozone. Geographical areas in which ozone is NOx controlled (red) and NOx‐saturated or VOC controlled (blue). The white area represents an intermediate situation.

Our first case focuses on the significant changes that took place in China during the 2020 lockdown (Zhang et al., 2020). To analyze the response of secondary species, it is first useful to determine the distribution of chemical regimes (VOC/NOx control of ozone) during the winter period (February). To estimate if a region is NOx‐limited or NOx‐saturated (VOC‐limited), we represent in Figure 5 the ratio *R* of the monthly mean production of H_2_O_2_ relative to the monthly mean production of HNO_3_. When *R* is greater than 0.2 (red zone), the ozone production is controlled by the level of nitrogen oxides, while if it is less than 0.06 (blue zone), the region is NOx‐saturated and the ozone formation is controlled by the atmospheric level of VOCs (Fu, Wang, et al., 2020; Tonnesen et al., 2000; Zhang et al., 2009). The white zone shown in the figure corresponds to an intermediate situation. We note that in continental areas, where the population density and the economic activity are low or moderate and over the oceans, ozone is as expected, NOx‐limited. In the north of China, in India, Korea, Japan, Kyrgyzstan, Kazakhstan, and certain highly urbanized zones (e.g., Hong Kong and Guangzhou, Taipei), ozone is NOx‐saturated and therefore VOC‐limited. This condition corresponds to a winter and early spring situation. In summer, however, when the concentration of NOx is lower, the area with VOC‐limited conditions is reduced. In China during the lockdown period, the limit between the VOC and NOx controlled regions is located along a line extending from approximately Lanzhou in the center of China to Xiamen along the ocean in the vicinity or Taiwan. Inside the NOx‐limited regions, urban centers are often VOC‐controlled. Since the ozone sensitivity is determined by the sources of odd hydrogen radicals, we show in Figure S8 the relative contribution of the two most effective contributions of surface HOx production in the North China Plain in winter and summer: the photolysis of formaldehyde (H_2_CO) and of nitrous acid (HONO).

**Figure 6:**
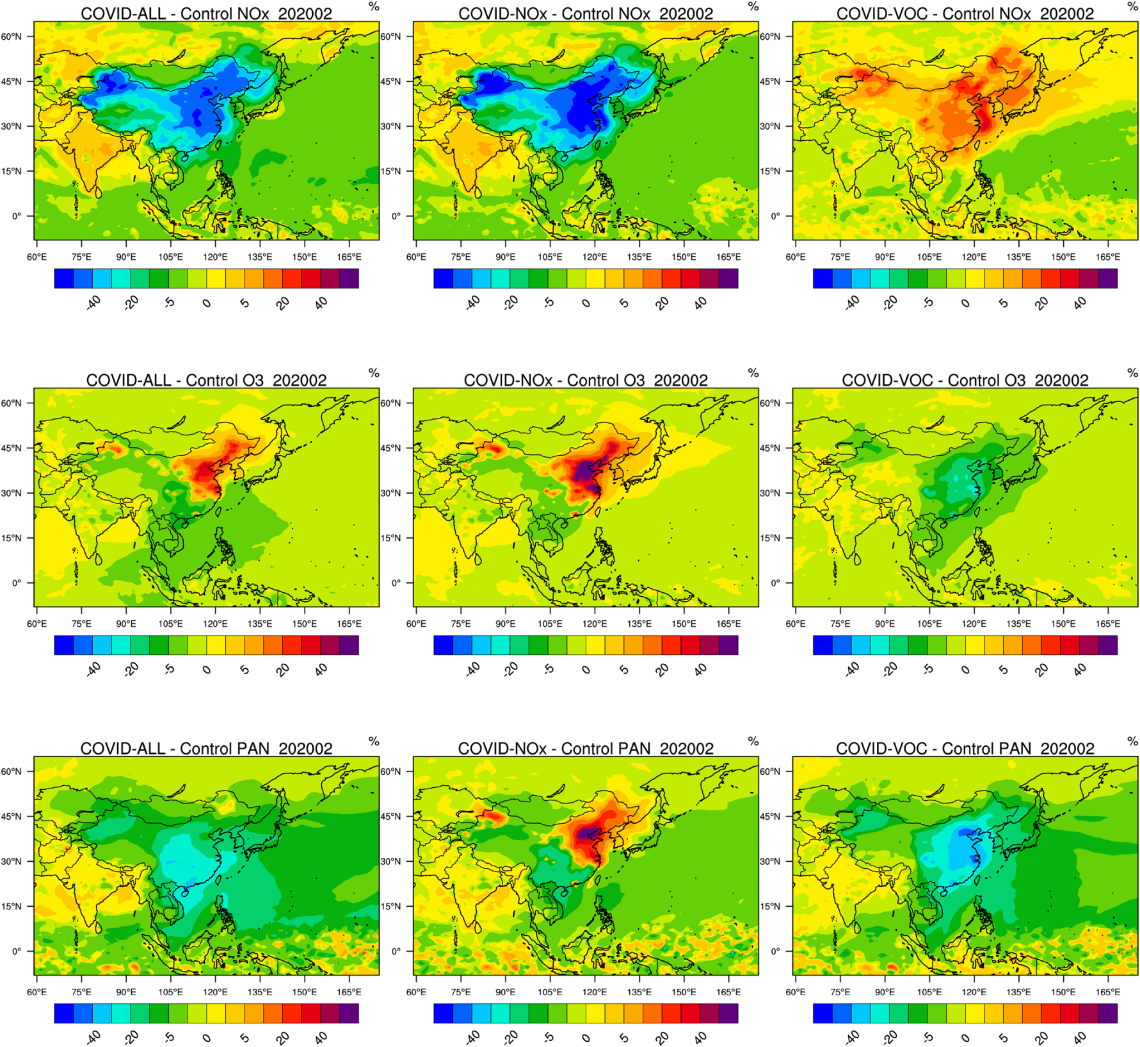
Percentage change in several chemical variables in China in response to reduced emissions of primary pollutants in February 2020 during the COVID‐19 pandemic. Panels from the top to the bottom: NOx, ozone, and PAN. Left column: reduction in all emission; center panel: reduction in NOx emissions only; right panel: reduction in VOC and CO emissions only. Results for other chemical species are provided in the supplementary information (Figure S9).

Figure 6 shows that, during the lockdown of February, the surface concentration of NOx was severely reduced (40%–50%) in most areas of eastern China and in the northwest of the country. At the same time, the concentration of ozone increased in the northeastern part of China and locally in several large urban areas of other regions as also evidenced by surface observations (e.g., Huang et al., 2020; Liu & Wang, 2020; Shi & Brasseur, 2020). Further, a reduction in ozone occurred in the southern part of the country. This result is consistent with the regional model study of Liu and Wang (2020) and with surface observations (e.g., Fu, Wang, et al., 2020; Lian et al., 2020). To further address this question, we show how surface NOx and ozone would have responded according to the model if only the emissions of VOC/CO or of NOx had been reduced.

#### 5.1.1 COVID‐VOC Case: Reduction Only in the VOC and CO Emissions

If only VOC and CO emissions are reduced, while the emissions of other species including NOx remain unchanged in China during February 2020 (Figures 6 and S9), the ozone and peroxyacetyl nitrate (PAN) concentrations as calculated by the model decrease in the North China Plain by 15%–25% and 30%–50%, respectively. A substantial reduction in the concentration levels of hydrogen radicals (HOx = OH + HO_2_) also occurs, but the concentration of NOx slightly increases due to the reduced loss rate via HNO_3_ formation, the reduced formation of organic nitrates and peroxy nitrates, and the reduced uptake of NOx by SOA. With the adjustment factors adopted for VOCs, the model derives a reduction of around 30% for OH, 50% or higher for HO_2_, 50% for CH_3_O_2_, 20%–30%, for formaldehyde, 15%–20% for hydrogen peroxide, and 15%–30% for nitric acid in the North China Plain (see Figure S9). The concentration of OH, however, is slightly enhanced (5%–10%) outside this particular region. The concentration of the NO_3_ radical, which is a major oxidant during nighttime, slightly increases (typically 2%–5%) in most regions of China except in the North China plain, where it decreases by as much as 30%. The decrease in the level of HOx directly results from the reduction in the sources of these radicals, including the reactions of alkenes with ozone, the photolysis of formaldehyde and of other carbonyls, and the photolysis of nitrous acid (HONO) since the heterogeneous formation of this last compound on the surface of aerosol particles is reduced as the aerosol concentration (including the concentration of SOA and sulfates) has decreased. Under the assumptions adopted here, the HO_2_ to OH ratio declines since the conversion of OH to HO_2_ by CO and VOCs is slowed down, while the conversion of HO_2_ back to OH slightly accelerates due to slightly enhanced NOx concentrations. We note that the relatively large reduction in HO_2_ and CH_3_O_2_ (more than 50%) together with a smaller increase in NOx (10%–30%) leads to a decrease in the photochemical production of ozone.

#### 5.1.2 COVID‐NOx Case: Reduction Only in the NOx Emissions

If we make a simulation in which only the NOx emissions are reduced during the pandemic, the response to the chemical system (Figures 6 and S9) is very different (opposite sign) than in the previous case. Under our assumption, the concentrations of OH and HO_2_ increase by 50% or more, mostly in the northeastern part of China. The concentrations of methyl peroxy (CH_3_O_2_) and formaldehyde (HCHO) increase by 10%–20%. The increase in HOx is attributed primarily to a reduced recombination of OH with NO_2_, which leads to the reduction in the HNO_3_ concentration derived by the model. Since the photolysis of HCHO is a significant source of HOx radicals, the increase in the concentrations of OH and HO_2_ radicals also results from the enhanced concentration of formaldehyde (Li et al., 2021). The reduction in the NO concentration tends to shift the balance between HO_2_ and OH toward HO_2_. The concentration levels of the NO_3_ radical and of PAN are enhanced in northern China and particularly in the region of Beijing (reaching more than 60% for both species), but are reduced in southern China. The response of ozone (increase in northern China of 30%–60% and decrease of about 5% in southern China) results from synergetic changes in both the production and destruction rates of the molecule. First, the simultaneous reduction in NOx, and enhancement in HO_2_ and CH_3_O_2_ concentrations result, according to the model, in a reduced photochemical ozone production rate of 20%–30%. Second, the titration of ozone by NO_2_, a major loss for ozone in the highly polluted areas of northern China, is reduced, while the direct ozone loss due to the enhanced levels of OH and HO_2_ increased. Taking into consideration these two processes acting in different directions, we find a resulting ozone loss that is reduced. This suggests that the most important factor explaining the ozone increase in northern China is the reduction of the ozone titration by NO_2_. In southern China, where the background levels of nitrogen oxides are lower and solar radiation intensity is higher, the reduction in NOx has led to an enhanced net ozone destruction and hence a reduction in the surface concentration of this molecule except in cities where the ozone concentration increases. As expected, the net ozone production rate calculated for February 2020 (Figure S9) is positive in northeastern China and negative in other regions.

#### 5.1.3 COVID‐All Case: Reduction in the NOx, VOC, CO, and Aerosol Emissions

The response of the surface composition, when all emission adjustments for the emissions are taken into consideration (Figures 6 and S9), leads to an intermediate situation between the two cases described above. In fact, the response of most chemical species to the NOx and VOC emission reduction generally happens in opposite directions. When the two effects are combined, the reduction in NOx is of the order of 40%–60% in the North China Plain and in the northwest of the country. The increase in HO_2_ and CH_3_O_2_ is of the order of 50% and that of OH around 30%. The mean concentration of the NO_3_ radical increases by upto 50% in the urbanized regions of Beijing and Shanghai. The decrease in HCHO concentration is limited to 10%–20%. PAN and SOA concentrations decrease only by a few percent in northern China, but decrease more substantially (20%–30%) in the central and southern parts of the country. The exact quantitative response of PAN and SOA depends critically on the relative amplitude of the VOC and NOx emission reduction. It would have been positive in the North China Plain during February, if the adopted VOC emission reduction had been somewhat smaller. In the case of SOA, a plume with decreased concentration values in noticeable over Korea, Japan, and the Western Pacific Ocean. The change in ozone is positive in the northeastern part of China (about 30%–40%) and negative in the southern part of the country (about 10%). As seen in Figures 6 and S9, the most pronounced changes in the concentration level of most chemical species are located in the North China Plain and further north. These results are consistent with the analysis of surface measurements performed by Shi and Brasseur (2020), Liu and Wang (2020), and Tang et al. (2020).

In order to provide some insight on the relative forcing effects of the emission reduction during the pandemic and of the meteorological variability, we provide in Figure S10 an estimate of the ozone anomaly generated by weather dynamics and by the combined effects of the two forcing factors. Wang and Zhang (2020) provide a detailed assessment of the effects of meteorological elements during the pandemic period. Our model simulations as nudged toward the MERRA‐2 meteorology show that, during February 2020 and relative to our 5‐year climatology, Eastern China was abnormally warm by 1.5–2.5 K and subject to high cloud fraction; northern China was 2–4 K warmer with cloud fraction lower relative to the previous 5‐year average. During this month, ozone anomalies associated with meteorological variability were dominant in the tropical regions south of China, but were relatively weak on the Chinese mainland. Abnormally low ozone was found along the border between China and Mongolia related to the abnormally high NO_2_ concentration calculated during February 2020. The increase in the monthly mean ozone concentration in the North China Plain (upto 5%) predicted by the model in response to meteorological anomalies adds to the ozone perturbation caused by the reduction in emissions. Our simulations suggest that chemical disturbances rather than meteorological anomalies explain the ozone concentration increase in the North China Plain during February 2020. Shorter time fluctuations linked to specific weather conditions should be considered in a finer analysis to explain, for example, the acute air pollution episodes reported in several urban areas during January and February 2020 (Li et al., 2021; Wang et al., 2020). In southern China, where the perturbed chemistry tended to reduce ozone, a small positive anomaly is visible along the South China Sea. The change resulting from the two simultaneous effects is however negative except in the urban zone of Guangzhou/Hong Kong/Macao. In short, the enhancement in the level of oxidants in the North China Plain appears to be primarily a direct consequence of the reduction on chemical emissions triggered by the pandemic, but could have been facilitated by unfavorable weather conditions.

### 5.2 Air Quality in Europe During the Pandemic

**Figure 7:**
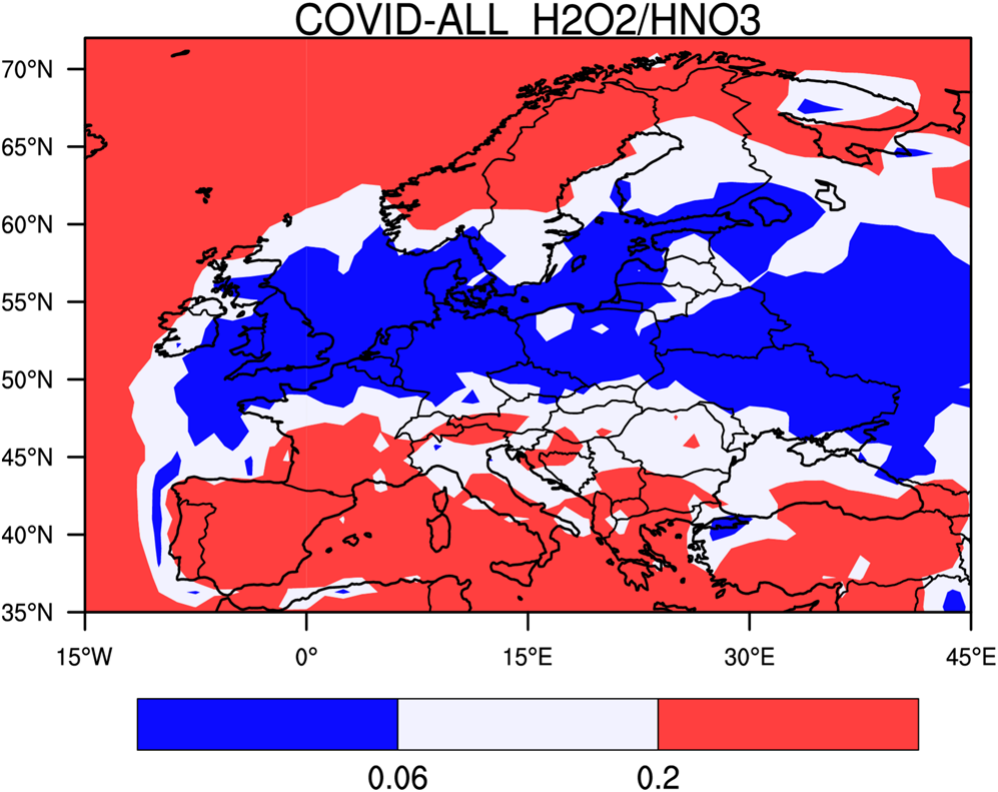
Ratio between the monthly mean production rate of hydrogen peroxide and nitric acid, a measure of the chemical conditions governing the formation of ozone. The geographical area in which ozone is NOx controlled is shaded in red and VOC controlled in shaded in blue. The white area represents an intermediate situation between fully NOx and VOC controlled situations.

The response of ozone in Europe resulted from a combination of chemical and meteorological processes. As in China, a small ozone increase was derived by our model in highly polluted areas, but the anomalies in the weather situation often played a dominant role. We first show in Figure 7 that, during the period of the lockdowns (15 March–15 April), the ozone production in most regions of Europe was controlled by NOx except in the most densely populated areas, where the influence of VOC was significant.

**Figure 8:**
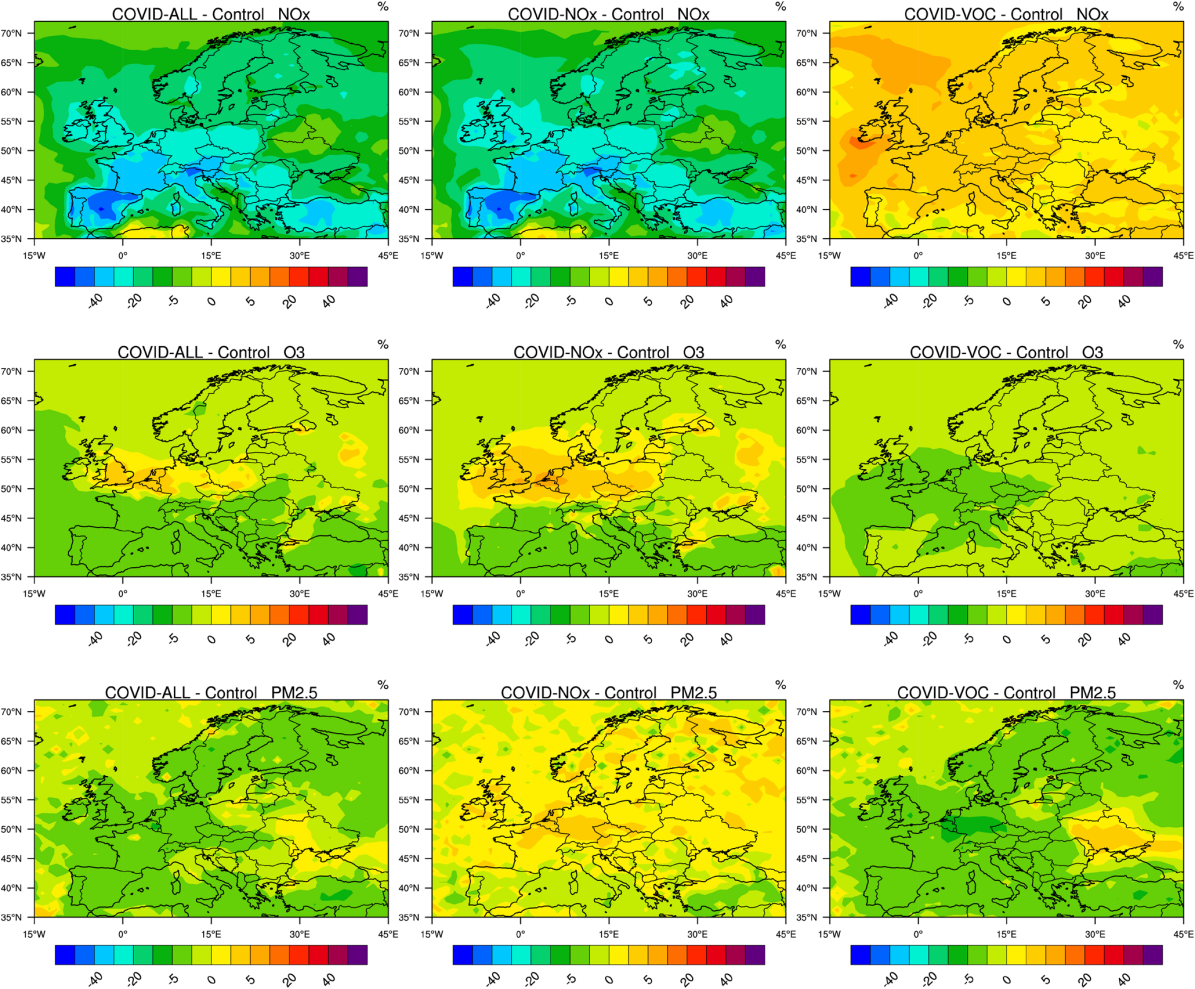
From top to bottom: percentage change in the surface NOx, ozone and PM2.5 concentrations across Europe in response to the emissions of primary pollutants adjusted for the COVID‐19 pandemic period of March 15–April 14, 2020. Left column: reduction in all emissions; center panel: reduction in NOx emissions only; right panel: reduction in VOC and CO emissions only.

In Figure 8, we show that, in March–April 2020, the relative reduction in NOx concentrations associated with the reduced emissions covers the entire European continent, but with the most pronounced effects occurring in the western and southern part of the continent (30%–50% in areas of Spain and France; 20%–30% in Germany, Switzerland, Eastern Europe, and 10%–30% in Scandinavia). This reduction is accompanied by an increase in the level of photooxidants (OH, ozone) that is most pronounced in the UK, Belgium, the Netherlands, northern France, and in the western part of Germany. The ozone increase in this area is typically 5%–10%, while the OH increase (Figure S11) reaches 30%. In southern Europe, ozone concentrations are reduced by 5%–10%. These results are consistent with the findings of Tang et al. (2020) and Deroubaix et al. (2021) based on their analysis of ozone anomalies in Europe. The net ozone production (Figure S11) slightly increases over the central and western parts of the continent with a notable exception in Spain. The largest values are found again in the region extending from the UK to western Germany with hot spots in several urban or industrial areas. These patterns of ozone change are consistent with the regional model simulations performed by Menut et al. (2020) for western Europe. These authors derive on March 28, 2020 ozone anomalies (relative to a “business as usual” reference case) that are positive in the geographical area extending from in northern France and the UK to Germany and Poland. Negative anomalies are found in southern France and Spain.

The same type of behavior is found in our global model when only NOx emissions are reduced, but with reinforced changes in secondary products. When only VOC emissions are reduced, ozone decreases by 2%–5% with the largest response located in an area extending from the Atlantic to Germany in the vicinity of the English Channel. In this area, OH concentrations (Figure S11) are 5%–10% lower than in the baseline case. Finally, we note again, in this particular case, a slight increase in the concentration of NOx (2% with higher values of 5% over the sea east and north of the UK) resulting from a reduced conversion of nitrogen species to nitro‐organic compounds.

PM_2.5_ concentrations are reduced by a few percent in most parts of western Europe. This result combines the decrease of about 5% derived in the COVID‐VOC case and an increase of 2%–5% calculated in the COVID‐NOx case.

**Figure 9:**
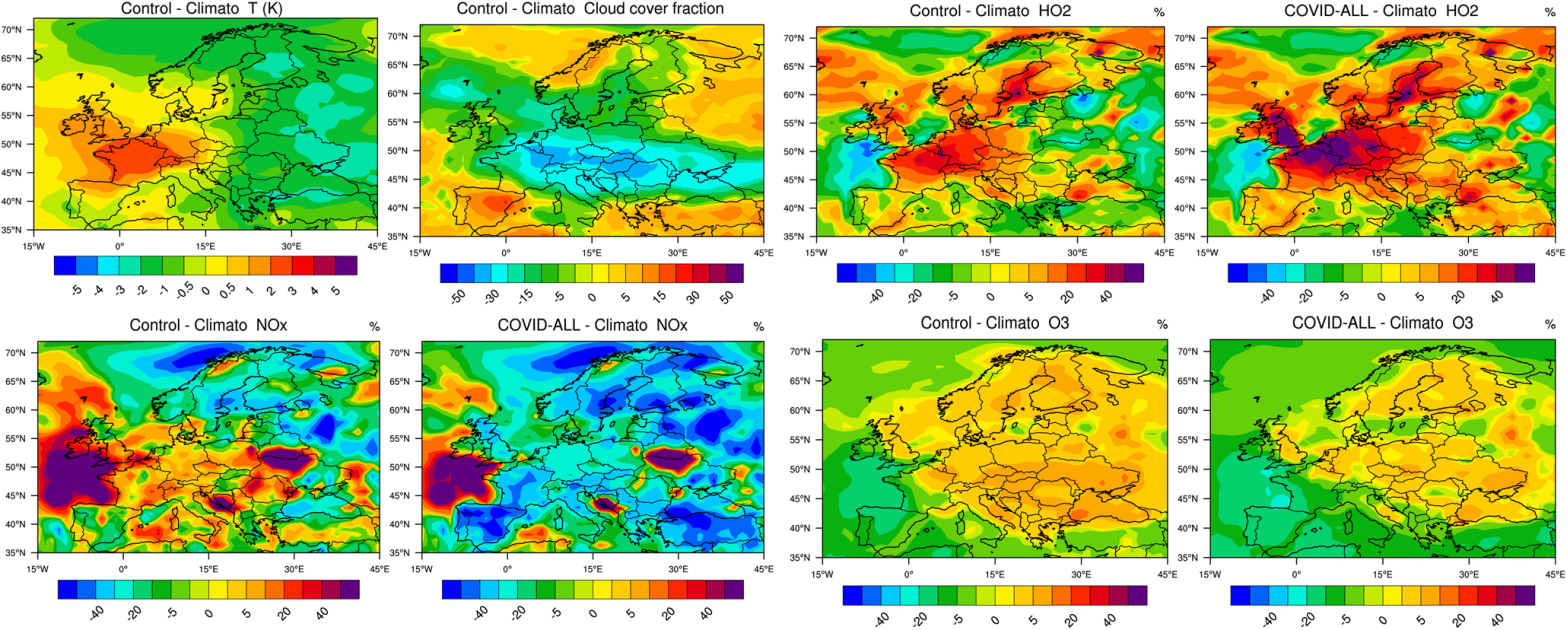
Change in temperature (K) and in the cloud fraction in Europe across Europe during the period March 15–April 14, 2020 relative to the value averaged over 5 years (2015–2019). Percentage change in the surface concentration of NOx, HO_2_, and ozone for the same conditions (Control − Climato). Response taking into account the adjustment of the emissions associated with the pandemic and the meteorological anomaly (COVID‐All − Climato).

We now examine the effects of meteorological anomalies on the calculated changes in the surface concentrations of NOx, HO_2_, and ozone. Figure 9 shows the changes in the concentration of several chemical species in response to the anomalies in the meteorology in March‐April 2020 (Control − Climato). Meteorological analyses (Deroubaix et al., 2021; Ordóñez et al., 2020) show that this period was characterized by unusual clear sky periods in central and northern Europe and cloudy skies in southwestern Europe. Figure 9 also shows the anomaly in temperature and in cloud cover calculated by the model for the month of April 2020 relative to a 5‐year climatology. During this particular month, the temperature is higher than the mean value in France, in Spain, near the Baltic Sea, and in Eastern Europe. Abnormal low cloudiness is predicted in Central Europe extending from France to the Black Sea and from Italy to Denmark. Cloudiness, however, is higher than normal in Spain, Turkey and part of Norway.

When considering only the effect of meteorological variability and ignoring the adjustments in the emissions, we see that during the 15 March–14 April period, the level of nitrogen oxides is abnormally high at the western edge of the European continent, as well as in France and in large parts of Italy and Central Europe. It is low along the eastern coast of Spain and in the southeastern part of Scandinavia. The change in NOx concentrations due to the meteorological effects (COVID‐All − Climato) is more pronounced than in the case in which only the emissions are adjusted to the COVID‐19 case (see Figure 8). Meteorological anomalies play therefore a substantial role. In the case of HO_2_, meteorological perturbations reinforce the disturbances due to the changes adopted for the emissions. The same reinforcement is also found in the case of ozone. In fact, for this particularly chemical species, meteorological anomalies are responsible for most of the changes in the surface concentrations. The ozone increase attributed to the combined reductions in NOx and VOC emissions is visible only in the region that covers the southern UK, the Benelux, and parts of Germany, as well as the eastern coast of Spain and areas in the Mediterranean. In summary, contrary to what has been found for China, a large fraction of the ozone increase noted in Europe during the pandemic must be attributed to meteorological anomalies (Deroubaix et al., 2021; Ordóñez et al., 2020); the reduction in pollutant emissions has substantially affected only a few specific regions of the continent.

### 5.3 Air Quality in North America During the Pandemic

**Figure 10:**
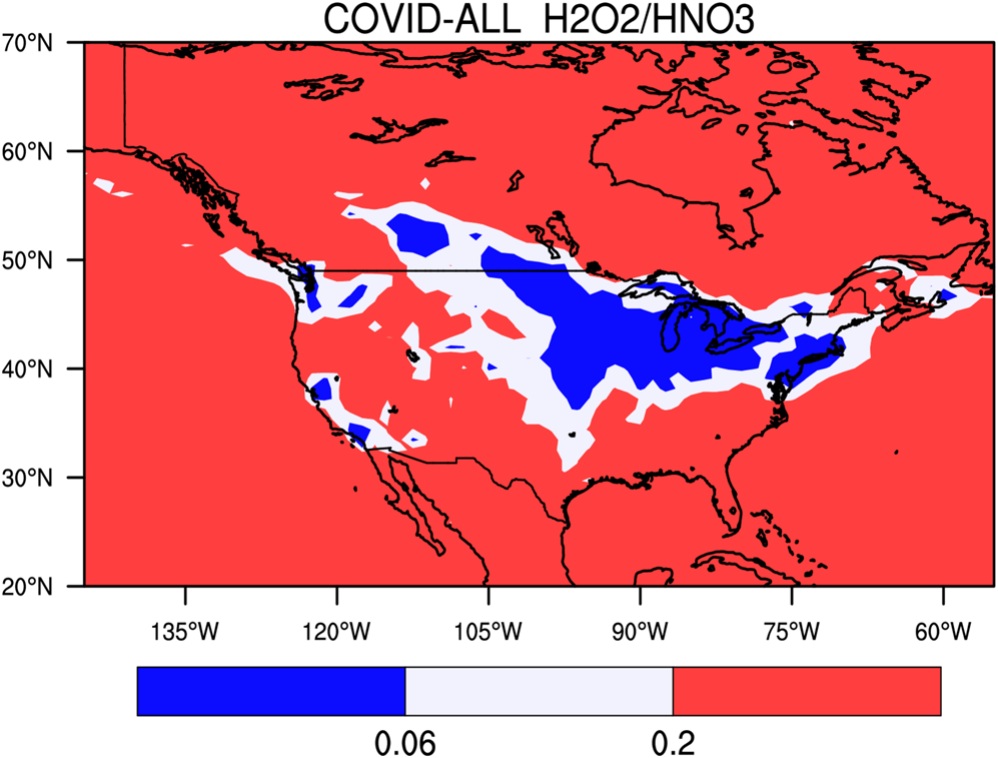
Ratio between the monthly mean production rate of hydrogen peroxide and nitric acid, a measure of the chemical conditions governing the formation of ozone. The geographical area in which ozone is NOx controlled is shaded in red and volatile organic carbon (VOC) controlled in shaded in blue. The white area represents an intermediate situation between fully NOx and VOC controlled situations.

We now examine the results provided by the model in North America (COVID‐All case) and focus again on the period ranging from mid‐March to mid‐April 2020. In most of the regions, particularly in rural areas, ozone is NOx‐limited during the spring conditions (Figure 10). However, in a region extending from the US East Coast to Alberta in Canada including the region of the Great lakes and part of the Middle West, ozone is VOC controlled.

**Figure 11:**
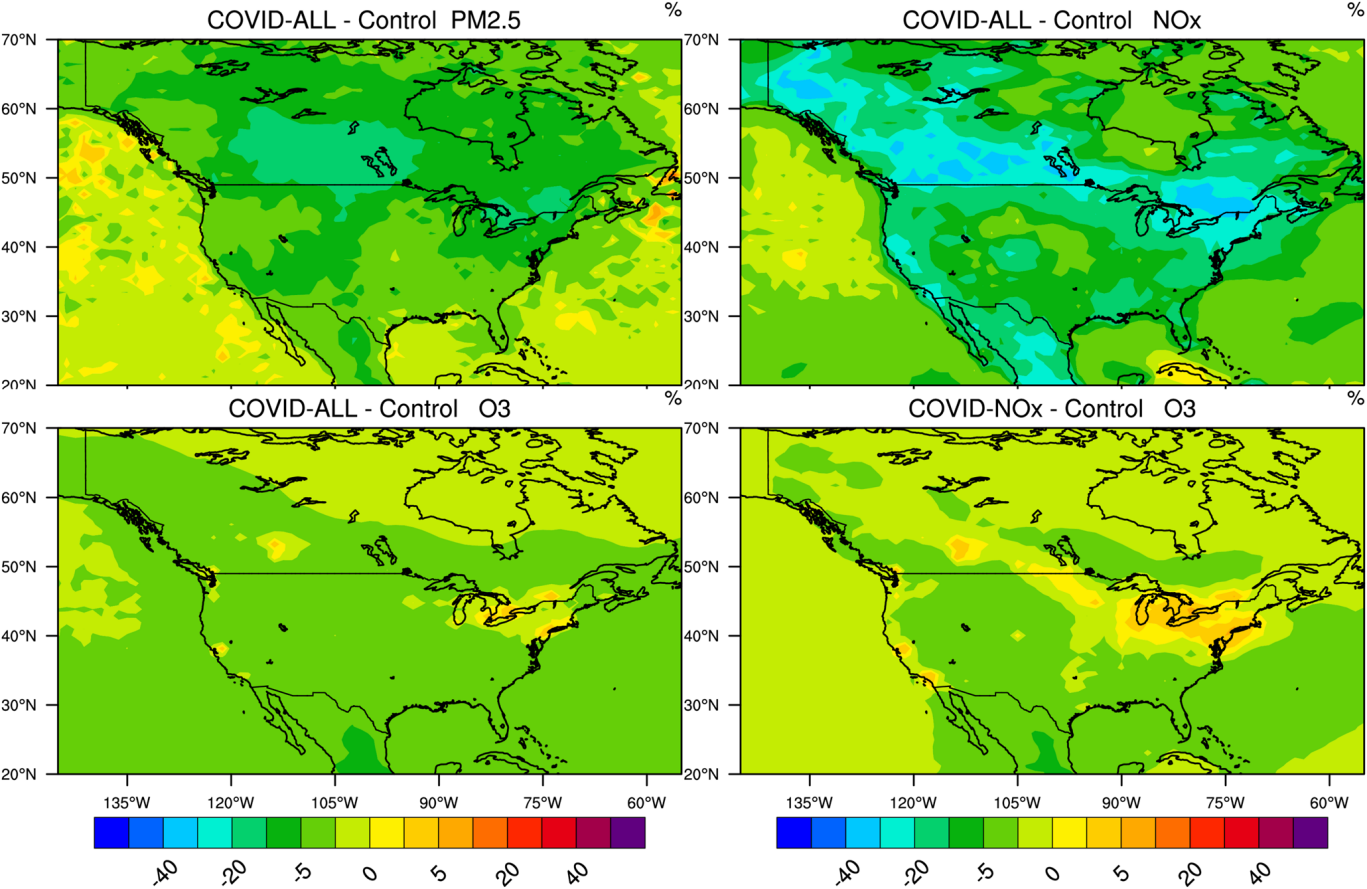
Percentage change in the surface concentration of (from top left to bottom right) PM_2.5_, NOx, and ozone across North America in response to adjusted emissions of primary pollutants during the COVID‐19 period of March 15–April 14, 2020. All calculated fields result from a COVID‐All simulation except the bottom right panel, which is obtained from a COVID‐NOx simulation (no reduction in volatile organic carbon [VOC] and carbon monoxide [CO] emissions). Model results for other species are found in the supplementary information (Figure S12).

In relative terms, the largest decrease in NOx concentrations is found in southern Canada (25%–40%) as well as in the northeastern US (30%–40%), notably near the Great Lakes and along the St Lawrence River (Figure 11). Substantial reductions in NOx are also noticeable along the west coast (20%–30%) and in the western and southern states of the US and in Mexico (20%–30%).

The surface concentration of the hydroxyl and peroxy radicals (Figure S12) has increased most in the region of the Great Lakes, along the US‐Canadian Border (including the region of Calgary), in the central plain of the US, as well as in urban areas of the west coast including Los Angeles, San Francisco and Seattle (5%–15% for OH, 30%–50% for HO_2_, and CH_3_O_2_). The reduction in formaldehyde is relatively small (less than 10%) except in southern Canada and the region of the St Lawrence, where it reaches 10%–20% (Figure S12). The change in the net ozone production rate during March–April is limited to a few percent and so is the change in the surface ozone concentration. Since ozone is NOx‐controlled in rural areas, the reduction in NOx leads to a small ozone decrease, mostly in the central and southern parts of the US. Only small ozone increases (2%–10%) in response to the changes in emissions are noticeable in the model results for the period 15 March–14 April, and are located around the Great Lakes, particularly near densely populated urban areas like New York, Boston, Toronto, Chicago, Calgary, Los Angeles, and San Francisco. The bottom right panel in Figure 11 provides the response of ozone resulting from a reduction in the NOx emissions only (no VOC and CO emission reduction, COVID‐NOx case). The patterns are the same as those discussed for the COVID‐All simulations with, however, more pronounced ozone increases along the US‐Canadian border and in the urban areas of the west coast. Chen et al. (2020) analyzed data acquired from 28 urban and suburban air quality stations across the United States that showed widespread nonuniform NOx reductions relative to a pre‐lockdown reference as well as mixed and relatively minor changes (less than 20%) in ozone. Tang et al. (2020) in their analysis of surface measurements showed that ozone increased only in the region of the Great lakes (3–6 ppbv) and near San Francisco (6 ppbv), and decreased slightly (upto 5 ppbv) or remained unchanged in the other areas of the United States, in good agreement with our model results. Additional model results are provided in Figure S12 of the supplementary information.

### 5.4 Air Quality in South America During the Pandemic

**Figure 12:**
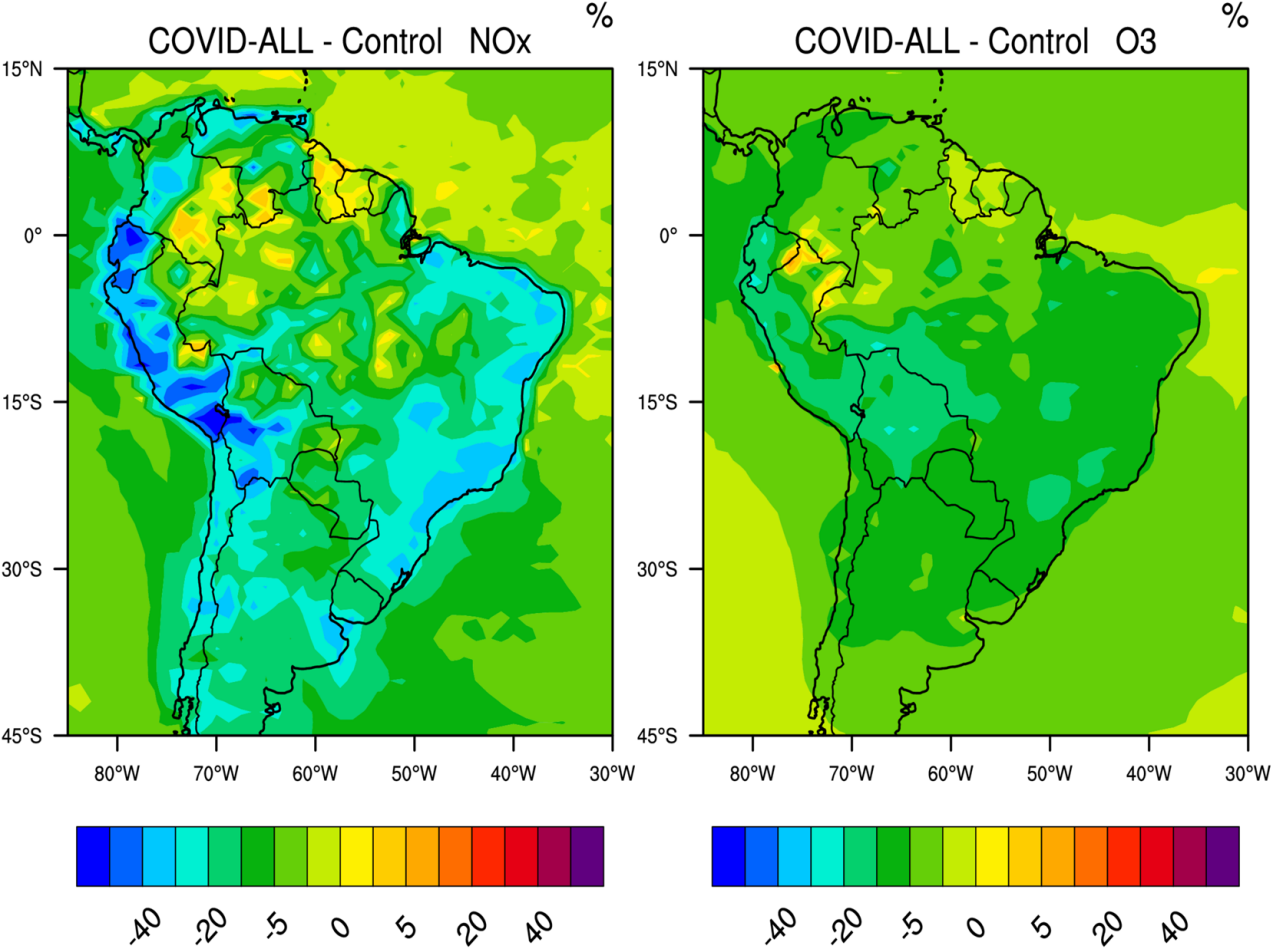
Percentage change in the surface concentration of NOx and ozone across South America in response to adjusted emissions of primary pollutants during the COVID‐19 period of March 15–April 14, 2020. Model results for other chemical species are found in the supplementary information (Figure S13).

In South America (Figures 12 and S13), a significant reduction in the surface concentration of nitrogen oxides is derived for the period 15 March–14 April, specifically along the Atlantic coast in Brazil (25%–35%) and the Pacific coast in Peru and Ecuador (30%–60%). Reductions of 30%–40% are also found in the region of Buenos Aires, Argentina and Santiago, Chile. The reduction in formaldehyde is generally limited to a few percent across the continent since a large source of this compound is due to biogenic emissions, which is unchanged in this simulation. Except in urban areas, the level of OH (Figure S13) decreases (20%–25% in eastern Brazil; 30%–40% in Peru and Ecuador). However, the concentration of HO_2_ (Figure S13) increases by 5%–15% in Chile, eastern Brazil and eastern Argentina, specifically in and near large South American metropolitan areas (Sao Paulo‐Rio de Janeiro region, Buenos Aires, Santiago, Lima, Guayaquil). The concentration of nitric acid decreases along both coasts (30% in Brazil; 40%–50% in Peru and Ecuador) and that of hydrogen peroxide slightly increases (up to 5%) in Chile, in the region of Sao Paulo and Rio de Janeiro as well as in the northern part of the South American continent. A small reduction in the surface ozone concentration (5%–10%) is derived in Brazil and a larger decrease (15%–20%) is calculated in Bolivia, Peru, and Ecuador. Cazorla et al. (2020) note that, in the city of Quito, Ecuador, the average ozone level during the lockdown in April was not significantly different from the ozone level in January, which they attribute to unusually high cloudiness and to frequent precipitation during the month of April.

## 6 Summary and Conclusions

The worldwide disruption in the economic activities during the COVID‐19 pandemic in early 2020 has generated large perturbations in the emissions of air pollutants. These perturbations have been prominent first in China, where the pandemic outbreak was reported and later in other countries of both hemispheres. The response of photooxidants to the simultaneous reductions in NOx, VOC, and CO emission has varied according to the geographic location and the time of the year. In the NOx‐saturated region of northeastern China, which was hit by the pandemic under winter conditions, an increase in the concentrations of ozone, OH, HO_2_, and RO_2_ radicals was derived by the model. The concentration of the NO_3_ radical, a powerful nighttime oxidant and of PAN, a secondary pollutant was also increased in the North China Plain. The reduced NOx emissions also led to less titration of ozone, a reduced conversion of OH by NO_2_ and an increased HO_2_/OH concentration ratio. Further, even though the intensity of solar radiation is low during February, the photochemical production of ozone and OH was not suppressed. However, the strong decrease in NO resulting from reduced activities during the pandemic was not compensated by a sufficiently large increase in peroxy radicals, so that the overall ozone production by the limiting HO_2_ + NO and RO_2_ + NO reactions was reduced during the month of February. The ozone concentration increase was therefore due primarily to a relatively larger reduction in the ozone loss. In the NOx‐limited region of southern China, the concentration of ozone and other photooxidants decreased because their formation rate favored by NOx was reduced, except in VOC‐limited urban areas like Guangzhou or Hong Kong, where the model predicted ozone enhancements.

In the other regions of the world during the peak of the lockdown period (corresponding to Northern Hemisphere spring and Southern Hemisphere fall), the oxidation level was also disturbed by the reduced emissions of ozone precursors. During April 2020, for example, the level of oxidants including ozone was enhanced in the regions of Europe where the background level of NOx is relatively high. In response to the perturbed emissions of pollutants, ozone concentrations increased in a region extending from the UK to Germany, and OH levels increased in most of western Europe except in Spain. In North America, the reduced emissions led to enhanced concentrations of oxidants along the US‐Canadian border and ozone concentrations increased slightly in the region of the Great Lakes. In South America, during this period of late summer and early fall, the level of photooxidants decreased except in metropolitan areas where elevated concentrations of OH and HO_2_ were calculated by the model.

The level at which the oxidizing capacity of the atmosphere changed in Northern China and to a lesser extent in Europe and North America, as well as the related increase in the concentration of secondary products such as ozone, OH, HO_2_, NO_3_, and PAN depends on the relative amplitude in the change in VOC and in NOx emissions. Both forcing processes act in different directions. Therefore, if the VOC emission reduction adopted here was overestimated, the formation of the secondary species would be somewhat underestimated. In this case, a more likely description of the response of the atmosphere during the pandemic should be intermediate between the fields provided by the COVID‐All and COVID‐NOx simulations.

These results are obtained by model simulations that isolate the changes in surface emissions and consider them as the only forcing mechanism. However, meteorological variability provides an additional forcing mechanism that produces substantial changes in the monthly mean concentrations of chemical species; these changes can be comparable and in some cases, larger than the chemical response to emission reductions. In China, although large‐scale meteorological anomalies as derived by the model during the month of February may have contributed to the ozone increase in the North China Plain, the largest effect should be attributed to chemical perturbations related to the reduction in emissions. In most areas of Europe, however, the situation was different: during the acute period of the pandemic between mid‐March and mid‐April 2020, most of the ozone increase calculated by the model was associated primarily with weather anomalies rather than the emission reduction. Chemical perturbations contributed significantly to the ozone increase, but only in a limited region extending from the UK to Germany and including the Benelux countries.

In summary, the simulations performed by the global atmospheric model (CESM v.2.2) with a detailed chemical scheme (MOZART TS1 mechanism) driven by emission changes of primary pollutants and forced by realistic weather conditions reproduce reasonably well the changes observed in the chemical composition of the atmosphere, and specifically in the perturbations of surface ozone and other oxidants during the COVID‐19 pandemic. At least qualitatively, the response of the atmosphere to the gigantic chemical experiment that took place in the atmosphere during the first half of 2020 is found to be explained to a satisfactory degree by our current understanding of the photochemical theory, in particular in what concerns ozone formation. This unexpected global event allows us, however, to address unresolved questions related to the nonlinear atmospheric system with its complex chemical regimes including the mechanisms that control the formation of secondary pollutants under different chemical environments. More detailed and specific studies that investigate regional responses to emission reductions together with mesoscale and local weather variability should be conducted with higher resolution models.

## Supporting information

Supporting Information S1

## Data Availability

CESM2.2.0 is a publicly released version of the Community Earth System Model and freely available online (at www.cesm.ucar.edu, last access: October 2, 2020). For Europe, the observational data set is provided by the Air Quality e‐Reporting (AQ e‐Reporting), available at https://www.eea.europa.eu/data-and-maps/data/aqereporting-8 (permanent link: b21a537e763e4ad9ac8ccffe987d6f77), last access: November 4, 2020. For São Paulo region, the observational data set is provided by the CETESB Network of the environmental state agency of São Paulo, available at https://qualar.cetesb.sp.gov.br/qualar/home.do, last access: November 4, 2020. For the North China Plain region, the observational data set is provided by the China Environmental Observation Network operated by the China National Environmental Monitoring Center, available at http://www.cnemc.cn/en/, last access: November 4, 2020. For the USA, the observational data set is provided by the US Environmental Protection Agency, Air Quality System available at http://www.epa.gov/ttn/airs/aqsdatamart, last access: October 26, 2020. This publication contains modified Copernicus Sentinel‐5 TROPOMI data for 2019–2020. TROPOMI data versions 1.2.2 and 1.3.0 used here are available at https://s5phub.copernicus.eu.
