# Supplementary material for: Global Changes in Secondary Atmospheric Pollutants During the 2020 COVID‐19 Pandemic
Source: J Geophys Res Atmos. 2021 Apr 27;126(8):e2020JD034213. doi: 10.1029/2020JD034213 (PMC8250227; doi:10.1029/2020JD034213)
Supplement: Supplementary file 1 — Supporting Information S1 [file JGRD-126-e2020JD034213-s001.docx]

*
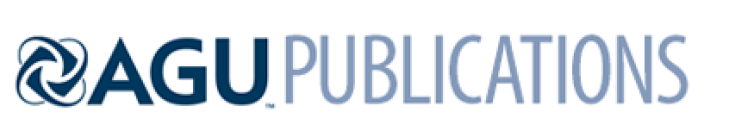
*

*Journal of Geophysical Research*

Supplementary Information

Global Changes in Secondary Atmospheric Pollutants during the 2020 COVID-19 Pandemic

Benjamin Gaubert^1^, Idir Bouarar^2^, Thierno Doumbia^3^, Yiming Liu^5^, Trissevgeni Stavrakou^6^, Adrien Deroubaix^2^, Sabine Darras^4^, Nellie Elguindi^3^, Claire Granier^3,7^, Forrest Lacey^1^, Jean-François Müller^6^, Xiaoqin Shi^2^, Simone Tilmes^1^, Tao Wang^5^, and Guy P. Brasseur^1,2,5^

^1^Atmospheric Chemistry Observations and Modeling Laboratory, National Center for Atmospheric Research, Boulder, CO, ^2^Environmental Modeling Group, Max Planck Institute for Meteorology, Hamburg, Germany, ^3^Laboratoire d’Aérologie, Université de Toulouse, CNRS, UPS, France, ^4^Observatoire Midi-Pyrénées, Toulouse, France, ^5^Department of Civil and Environmental Engineering, The Hong Kong Polytechnic University, Hong Kong China, ^6^Royal Belgian Institute for Space Aeronomy, Brussels, Belgium, ^7^NOAA Chemical Sciences Laboratory/CIRES, University of Colorado, Boulder, CO.

Corresponding author: Guy P. Brasseur (guy.brasseur@mpimet.mpg.de)

**Text S1**: Evolution of the changes in the emissions of primary pollutants is displayed during the first months of the COVID-19 pandemic.


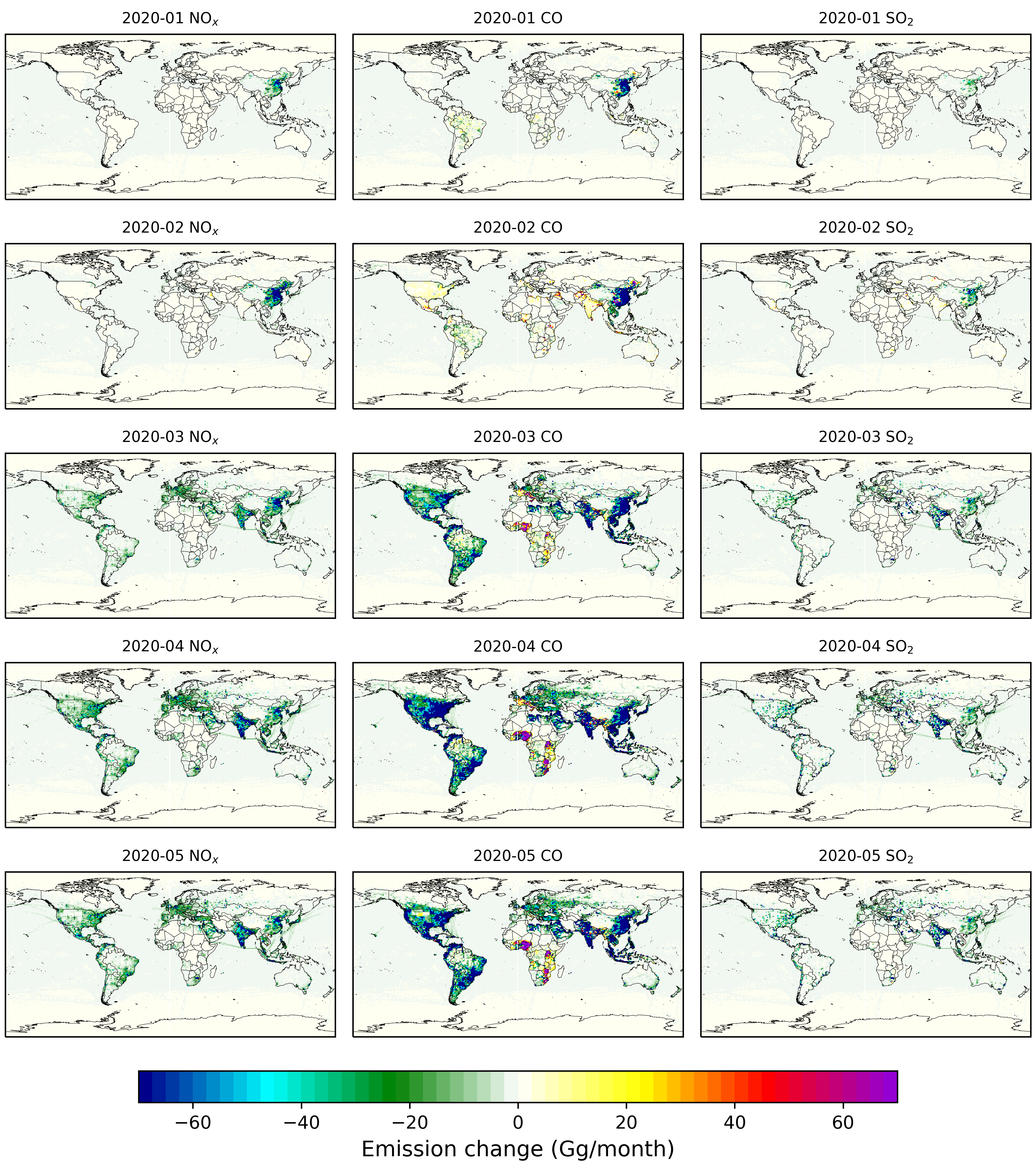


**Figure S1**. Change in the monthly mean emissions of NOx (left) CO (middle) and SO_2_ (right) from January 2020 (upper panels) to May 2020 (lower panels). The values are expressed in Gg per month for each grid point.

**Text S2.** Baseline distribution of the surface mixing ratios of different chemical species calculated by the CESM model for February and April 2020.


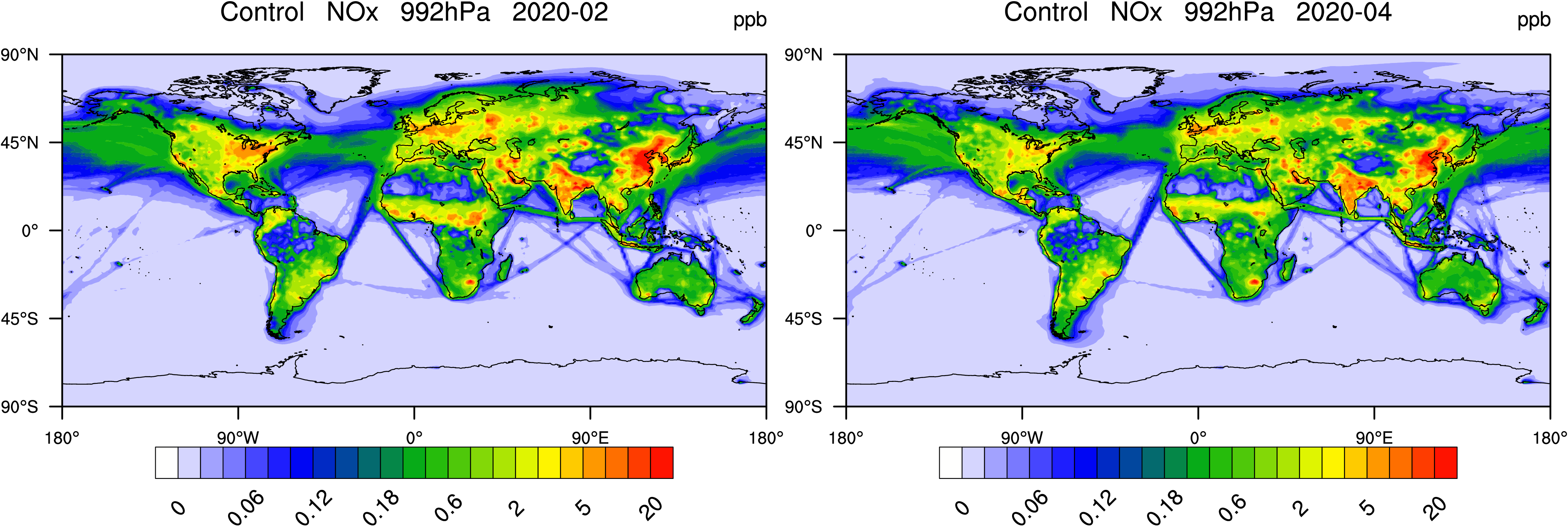


**
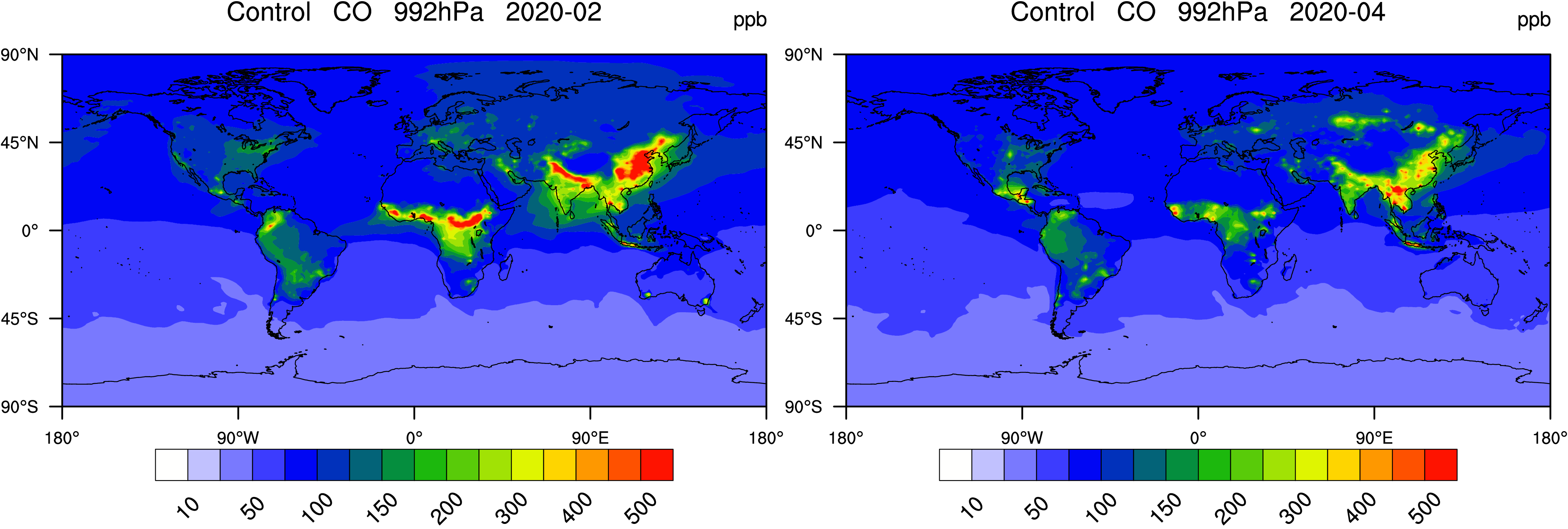
**

**
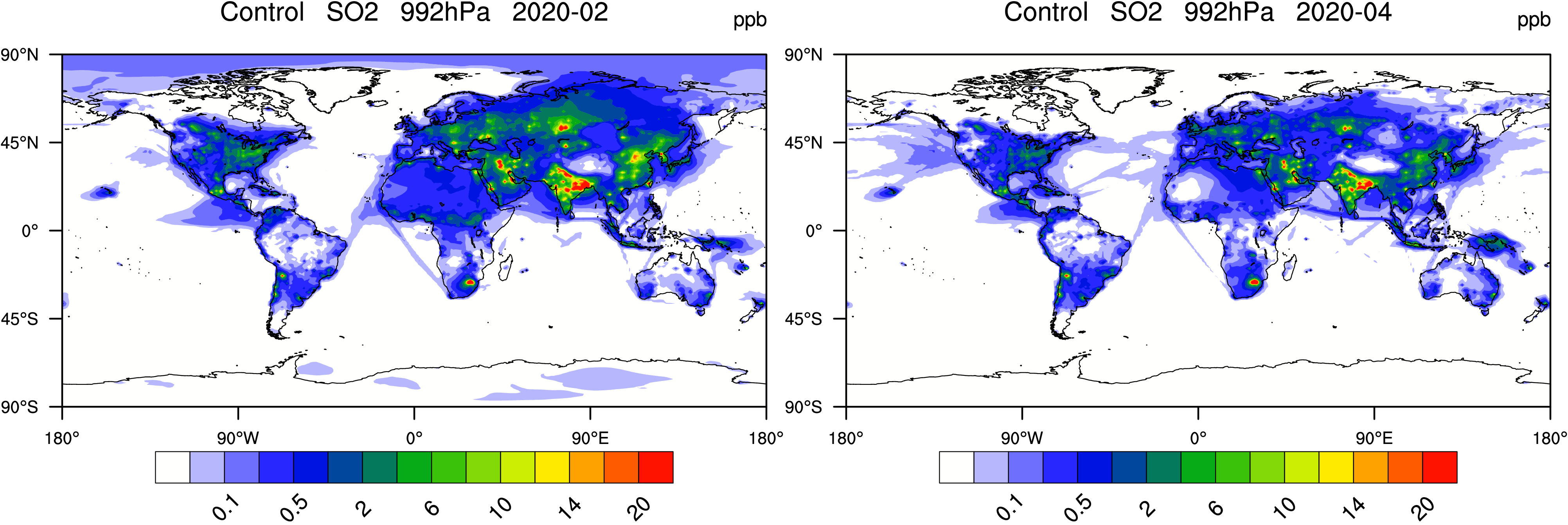
**

**
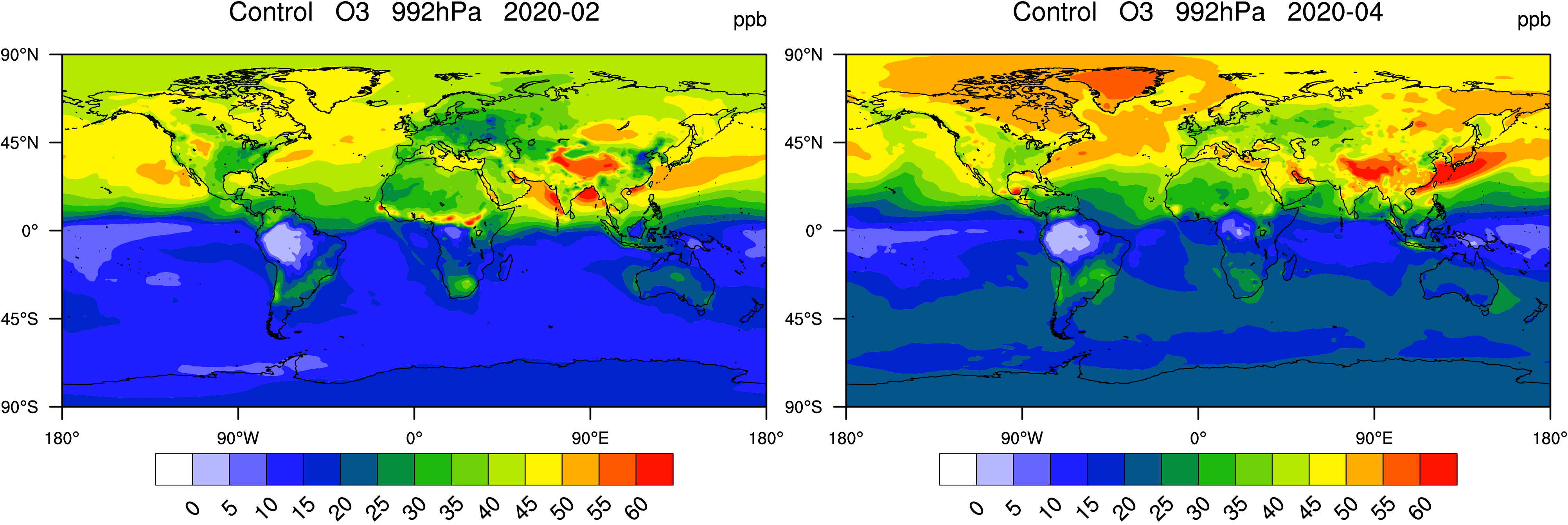
**

**
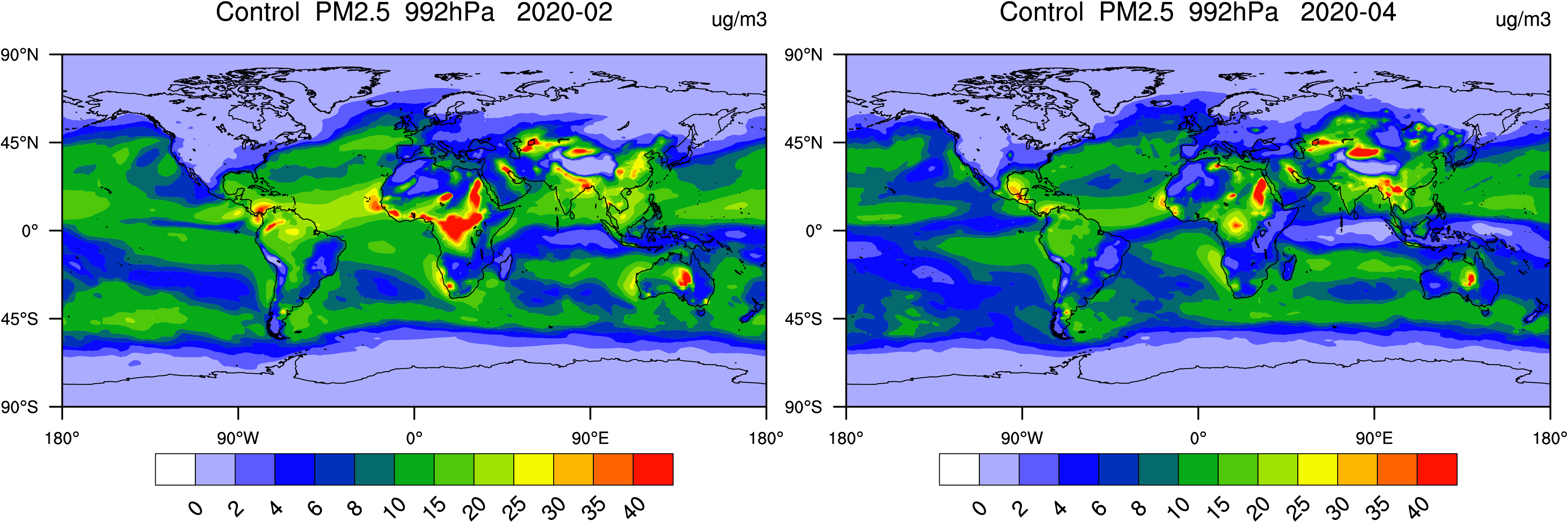
**

**Figure S2**. Global distribution of the surface mixing ratio of (from top to bottom) NO_2_, CO, SO_2_, O_3_ and PM_2.5_ (ppbv) for the month of February (left column) and April (right column) resulting from the control simulation of the CESM model.

Text S3 and S4a-h. Model description and evaluation

**The Community Earth System Model version 2.2 (CESM2.2)**

The global distributions of chemical species presented here are provided by the Community Earth System Model (CESM) version 2.2 that accounts for interactive physical, chemical and dynamical processes (Danabasoglu et al., 2020). The atmospheric component of CESM, the Community Atmosphere Model (CAM-Chem), provides a comprehensive description of atmospheric chemistry and aerosol processes (Gettelman et al., 2019; Tilmes et al., 2019; Emmons et al., 2020; Gaubert et al., 2020) with a spatial resolution of 1.25° in longitude by 0.95° in latitude (about 100 x 100 km^2^ at mid-latitude) and with 32 vertical layers from the surface to the pressure height of 3.6 hPa (about 40 km altitude). The MOZART Troposphere Stratosphere (TS1) chemistry mechanism includes 221 gas phase and aerosol species and 528 chemical and photochemical reactions, and provides therefore a rather comprehensive explicit and interactive representation of tropospheric and stratospheric chemical processes (Emmons et al., 2020). Aerosols are represented by the four-mode Modal Aerosol Model (MAM4, Liu et al., 2016; Mills et al., 2016). The updated Secondary Organic Aerosol parameterization includes the Volatility Basis Set (VBS) approach discussed by Tilmes et al. (2019).

In order to accurately represent the meteorological conditions, the wind velocity components and the temperature are nudged towards the Modern-Era Retrospective Analysis for Research and Applications version 2 (MERRA-2, Gelaro et al. 2017) meteorological analysis. The data from the three-hourly meteorological horizontal wind and temperature analysis are regridded to the CAM-chem horizontal and vertical resolution, and nudged at every CAM-Chem physical step (30 min) with a Newtonian relaxation of about 6 hours. The ocean and sea-ice interfaces are prescribed from a climatology.

The model includes a two-way coupling between the atmosphere and the Community Land Model version 5 (CLM5; Lawrence et al., 2019), where the deposition of gases and aerosols is calculated. Biogenic emissions are calculated online in CLM5 with the Model of Emissions of Gases and Aerosols from Nature (MEGAN v2.1; Guenther et al., 2012). Daily biomass burning emissions are based on the biomass burning CO_2_ emission inventory available from the Quick-Fire Emissions Dataset (QFED; Darmenov and Da Silva, 2014). The chemical speciation is derived using the Fire INventory from NCAR (FINN) emissions ratios.

The anthropogenic emissions are specified according to the CAMS-GLOB-ANT_v4.2-R1.1 global inventory described by Granier et al. (2019) and Elguindi et al. (2020). This inventory provides monthly-averaged emissions of the main chemical compounds and 25 speciated volatile organic compounds for the 2000-2020 period at a spatial resolution of 0.1x0.1 degree. It is based on the EDGARv4.3.2 inventory developed by the European Joint Research Center (Crippa et al., 2018) and the CEDS emissions (Hoesly et al., 2018), which provide historical emissions for the 6^th^ IPCC Assessment Report (AR6). EDGARv4.3.2 emissions are available until 2012: the emissions are linearly extrapolated to 2020 according to the trends derived from the CEDS emissions for the years 2011-2014. For China, the emissions from MEIC1.3 (Zheng et al. 2018) are used to account for the recent decrease in the emissions of most pollutants in this region. Daily averaged emissions obtained from an interpolation between values at the 15^th^ of the month assumed to be equal to the monthly averaged emissions. These daily emissions are used for the baseline simulations and are adjusted for COVID-related runs by applying the daily adjustment factors discussed in Section 2 (Doumbia et al., 2021).

**Model evaluation**

We provide some insight about the performance of the CESM model regarding its ability to reproduce the observed concentration of reactive species at the surface. Since a detailed evaluation of the model is beyond the scope of this paper and has been performed in earlier studies (Emmons et al., 2020), we only compare the calculated time series of ozone and other key atmospheric species (NO_2_, CO, SO_2_, PM_2.5_) with measurements made at selected locations in different parts of the world. We first show in Figure S3 the global distribution of the monthly mean difference between 2019 and 2020 in the monthly mean NO_2_ column derived from TROPOMI measurements in February and compare it with the change in the column as calculated by the model. The comparison between the two figures should be viewed as qualitative since the TROPOMI data (Bauwens et al., 2020) are representative of early afternoon measurements while the model results refer to 24-hour average values and for not adjusted for the average kernel associated with the satellite observations. Further, both 2019 and 2020 simulations with the meteorology specific for each year have been performed with the same background emissions (case 2). In the 2020 case, however, the COVID-adjusted emissions are accounted for (case 3). What needs to be stressed here is that, in addition to the NO_2_ decrease seen in China during the month of February in response to the economic slowdown, a significant reduction in the NO_2_ column is found in northern Europe, in the North Atlantic and in the northern part of the US. Since no lockdown was imposed at that time in Europe and in North America, these changes should be viewed as a fingerprint of meteorological variability. Barré et al. (2020) and Goldberg et al. (2020) stress that meteorological variability complicates the analysis of observed data, and that such natural variations have a large effect when comparing, for example, observations between two consecutive years. Keller et al. (2021) accounted for meteorological variability by using a machine learning algorithm based on a large number of NO_2_ and ozone observations from January through June 2020. The reduction in NOx observed in northern Europe during February has generated an increase in ozone (see Figure 4 in the paper). Interestingly, both the model and the space observations also show a small NO_2_ increase in the southern and eastern part of the US and in the western part of Russia.


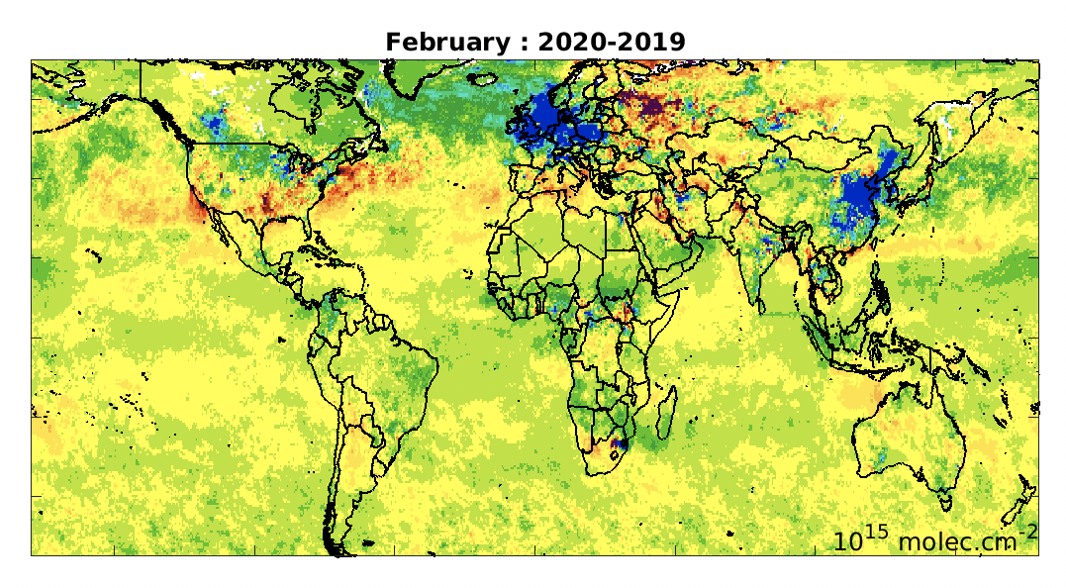

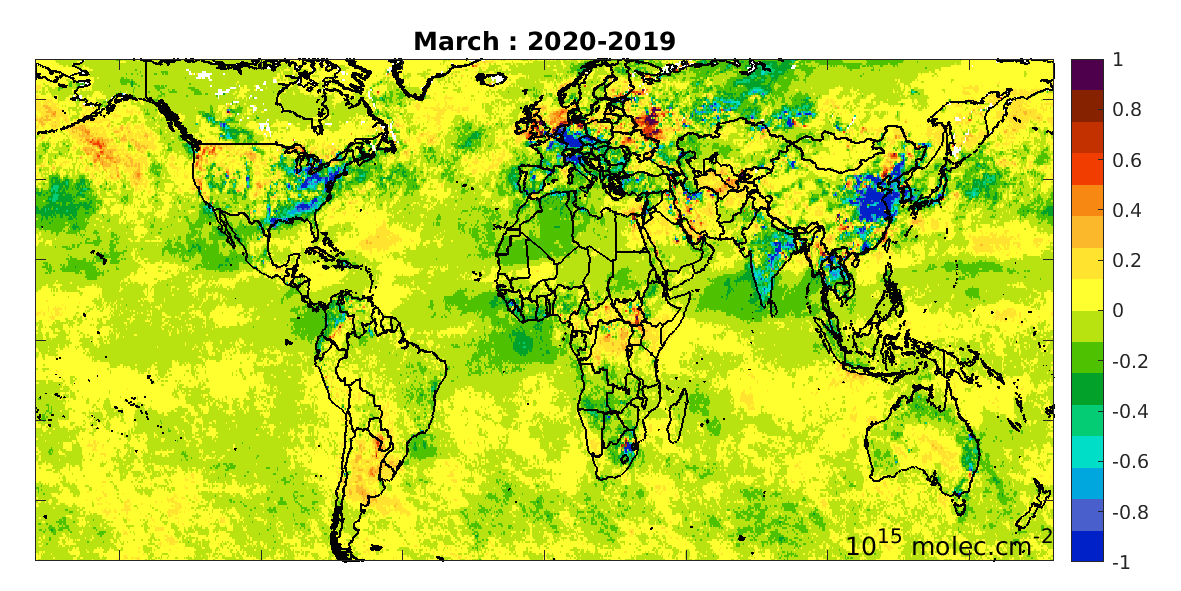


**
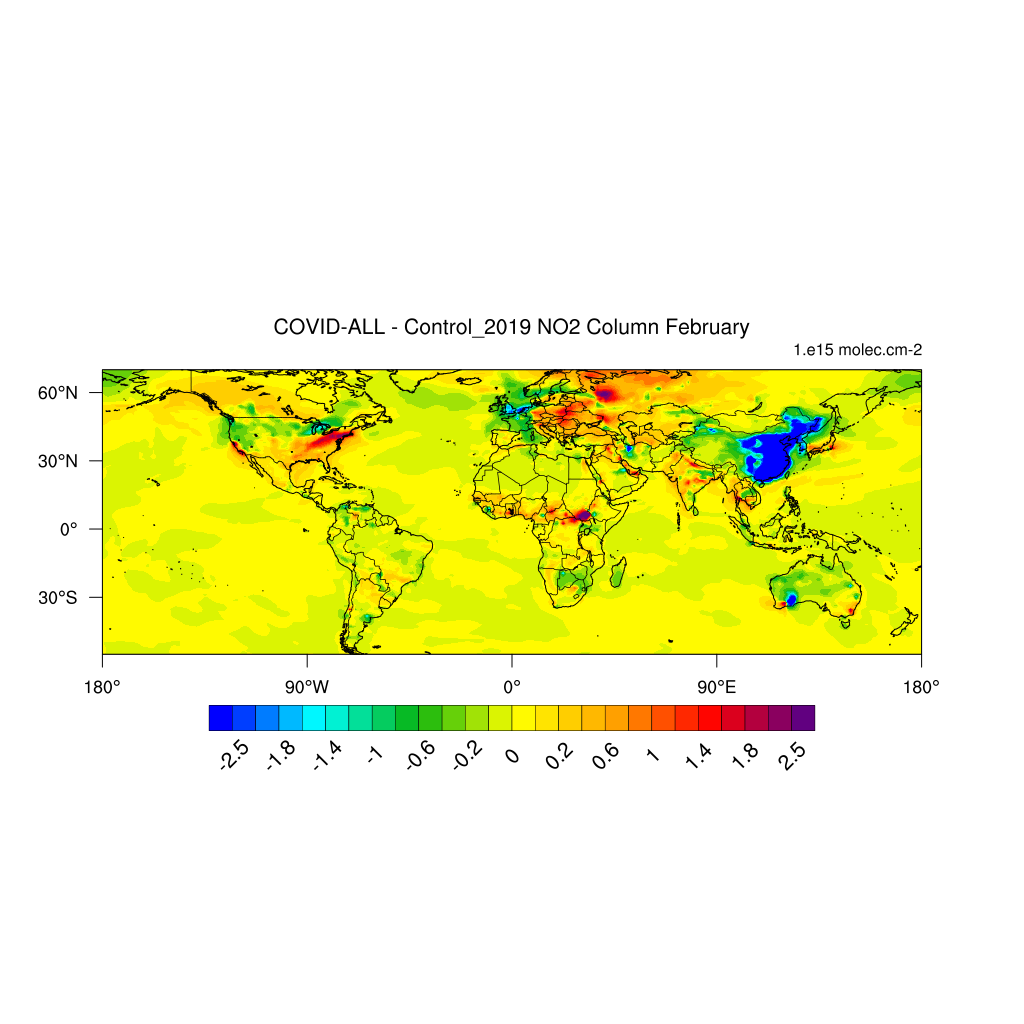
**

**Figure S3**. Difference between February 2020 and February 2019 in the global distribution of the monthly mean NO_2_ column abundance (10^15^ cm^-2^) Upper Panel: Observed values derived from TROPOMI satellite instrument. Lower Panel: Model values based on 2019 and 2020 simulations accounting for the reduction in surface emission and meteorological variability (2020 in case 3 minus 2019 in case 1).

We provide comparisons of calculated and observed time series for surface concentrations of different chemical species in selected regions where measurements are available. We show in Figure S4a-g the evolution of the species concentration between 1 January and 31 July 2020 for two model cases: (1) a baseline case with no correction for COVID-19 effects (case 1 or control case; green curves in the figures) and (2) a perturbed case with emissions adjusted as described above to account for the COVID-19 effect (case 2 or COVID-All case; red curves in the figures). We also show as supplemental information (Figure S4h) the evolution of the ozone mixing ratio averaged over areas of about 100 x 100 km^2^ around 6 European cities. The observations represent an average of the measurements made at monitoring stations in these areas. The outputs of the simulations are first interpolated to the location of these stations and, as for the observational data, are averaged over the chosen area.

In most regions, the model reproduces relatively well the chemical composition of the lower layers of the atmosphere. The long-term average evolution of the concentrations calculated by the model are influenced mainly by emissions from primary species and by chemical processes that occur in the atmosphere, while day-to-day variability is directly dependent on atmospheric dynamics and other meteorological processes. Data from monitoring stations are influenced by local emissions and by small-scale dynamic processes that cannot be captured by a model with a relatively coarse spatial resolution. Further, monitoring stations are often located in areas prone to high air pollution. Polluted areas may therefore be oversampled relative to the spatial averages provided by the model. In Europe and in the Sao Paulo region, we address this issue by removing from our analysis the data from stations located in areas with a population density larger than 1500 inhabitants per km², which is the threshold adopted by the European Union to define “high-density areas”.

We evaluate the model results by comparing the surface concentrations averaged over relatively large areas with the average of measurements made in these areas (Table S1). Model outputs are bi-linearly interpolated from the model grid points to each selected station. We then calculate the mean concentration and the standard-deviation of all selected stations to construct the daily time series. In all cases, we retain the stations for which 70% of hourly data are available. We then reject the stations for which more than 10% of the daily averages are missing. In Europe, we also ignore stations in heavily urbanized areas (more than 1500 inhabitants per km^2^) as well as stations whose altitudes are higher than 300 m. For the North China Plain, only the first criterion (availability of more than 70% of hourly data) is used because this flat area is strongly urbanized.

**Table S1** Domain names, acronyms, coordinates of the south-western and north-eastern corners of the domain’s frames, and the number of stations per species retained in the present study.

| Domain Name | Domain Acronym | Coordinates | Species (Number of Stations) |
| --- | --- | --- | --- |
| North China Plain  (China Environmental Observation Network) | NCP | (112°E; 34°N) to  (119°E; 42°N) | NO_2_ (220), CO (220), O_3_ (220), SO_2_ (220), PM_2.5_ (220) |
| Northern Europe  (AQ e-Reporting Database) | N-Eu | (1°W; 47°N) to  (11°E; 54°N) | NO_2_ (151), CO (16), O_3_ (156), SO_2_ (35), Ox (115), PM_2.5_ (53) |
| Southwestern Europe  (AQ e-Reporting Database) | SW-Eu | (9°W; 39°N) to  (3°E; 45°N) | NO_2_ (32), CO (9), O_3_ (42), SO_2_ (19), Ox (29), PM_2.5_ (10) |
| Northeastern USA and Southern Canada  (Air Quality System Data Mart Database EPA) | NE-US | (126°W; 36.5°N) to  (94°W; 52°N) | NO_2_ (122), CO (81), O_3_ (233), PM_2.5_ (257) |
| Southern USA  (Air Quality System Data Mart Database EPA) | S-US | (94°W; 24°N) to  (66°W; 36.5°N) | NO_2_ (43), CO (27), O_3_ (113), PM_2.5_ (86) |
| Western USA  (Air Quality System Data Mart Database EPA) | W-US | (94°W; 24°N) to  (66°W; 52°N) | NO_2_ (243), CO (120), O_3_ (417), PM_2.5_ (291) |
| Sao-Paulo  (CETESB Network) | Sao-P | (47.5°W;24°S) to (45.5°W;22°S) | NO_2_ (6), CO (1), O_3_ (47), SO_2_ (3), Ox (4), PM_2.5_ (3) |

The model results obtained for the North China Plain (32^0^ – 40^0^ N, 112.5^0^ – 120^0^ E) show that the model satisfactorily reproduces the concentrations of NO_2_, ozone and Ox (= O_3_ + NO_2_) as well as their temporal variability (Figure S4a). The model also underestimates the CO concentration by a factor of 1.5-2 during and after April 2020. In the case of SO_2_, the agreement of the model with the observation is satisfactory, except during the months of January and February when the modeled concentrations and their temporal variability are considerably higher than in the observations. During this period, the model also underestimates the concentrations and temporal variability of atmospheric particles (PM_2.5_) and CO. The repeated high peaks in PM_2.5_, which are observed, but whose amplitude is underestimated by the model, show acute haze episodes as those reported for example in Beijing (Li et al., 2021).

In northern Europe (Figure S4b), the calculated concentrations of NO_2_, ozone and Ox are generally in good agreement with observations. However, the calculated concentrations of CO are about 40% lower than the observations. This discrepancy, also noted in Northern China in April and May, repeats itself in most regions of the northern hemisphere and remains an open scientific question since it appears in most models unless their emissions have been artificially increased (Stein et al., 2014) or observationally constrained using data assimilation (Gaubert et al., 2016, 2020). The calculated SO_2_ variability is larger than observed and the concentration values generally higher than observed.

In southwestern Europe (Figure S4c), the concentrations of CO, NO_2_, SO_2_ and PM_2.5_ are underestimated by the model, while the concentrations of ozone and Ox are overestimated.

In the USA (Figures S4d-f), the concentrations of NO_2_ are in fair agreement with measurements in the northeastern (NE) US and southern Canada; there are, however, lower than observations in the southern and western regions of the US. Ozone is in fair agreement with measurements in the NE US, but overestimated in the southern and western parts of the country. CO is underestimated in all regions. PM_2.5_ are underestimated in the NE and western US and in good agreement in the southern region.

In Sao Paulo, Brazil (Figure S4g), the temporal evolutions of ozone, Ox and PM_2.5_ are correctly simulated despite a large underestimation of NO_2_ (factor 3-4). SO_2_ is underestimated by a similar factor. It should be noted, however, that, in this very heterogeneous megapolis, the NO_2_ and SO_2_ measurements vary greatly from one location to another and that these differences cannot be taken into account by a model whose mesh size is of the same order of dimension of the city. CO concentrations are in fair agreement with observed values.


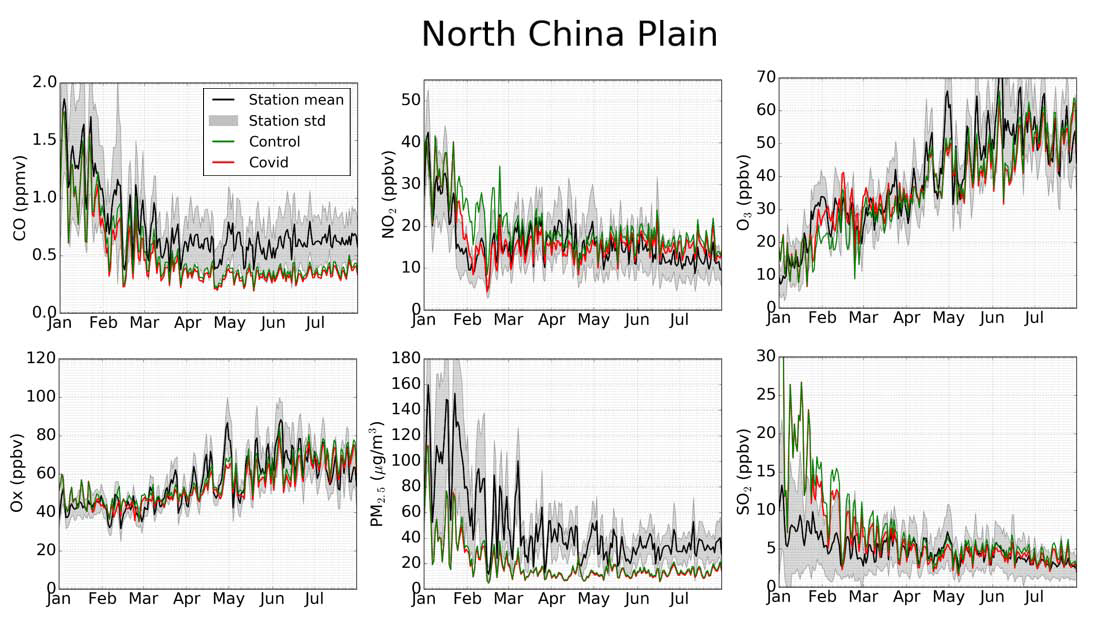


**Figure S4a.** Evolution for the period 1 January – 31 July 2020 of the surface concentration of NO_2_, CO, O_3_, Ox, SO_2_ and PM_2.5_ in the North China Plain. Black curve: measurements from monitoring stations. Green curve: model control case. Red curve: model case with emissions modified to account for the effect of the COVID-19 pandemic.


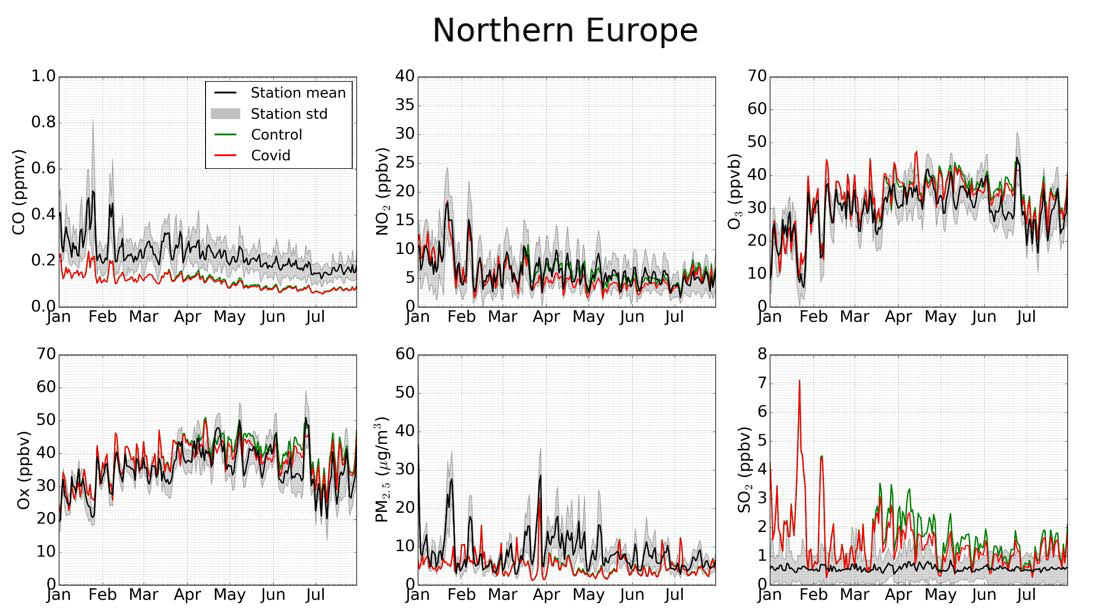


**Figure S4b.** Evolution for the period 1 January – 31 July 2020 of the surface concentration of NO_2_, CO, O_3_, Ox, SO_2_ and PM_2.5_ in northern Europe. Black curve: measurements from monitoring stations. Green curve: model control case. Red curve: model case with emissions modified to account for the effect of the COVID-19 pandemic.

**
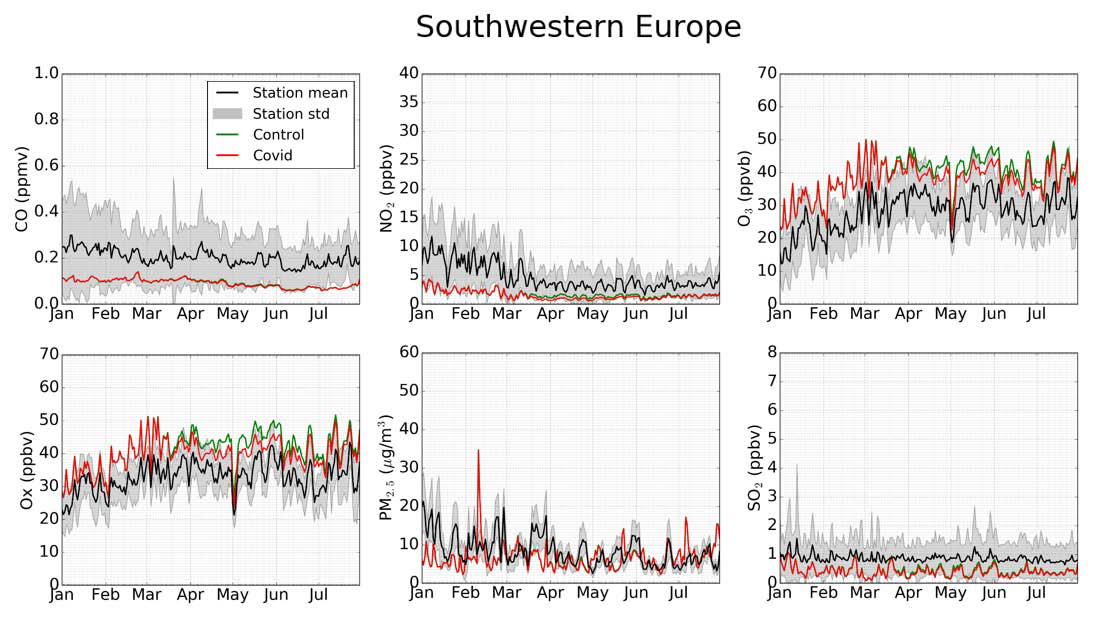
**

**Figure S4c.** Evolution for the period 1 January – 31 May 2020 of the surface concentration of NO_2_, CO, O_3_, Ox, PM_2.5 and_ SO_2_ in southwestern Europe. Black curve: measurements from monitoring stations. Green curve: model control case. Red curve: model case with emissions modified to account for the effect of the COVID-19 pandemic.


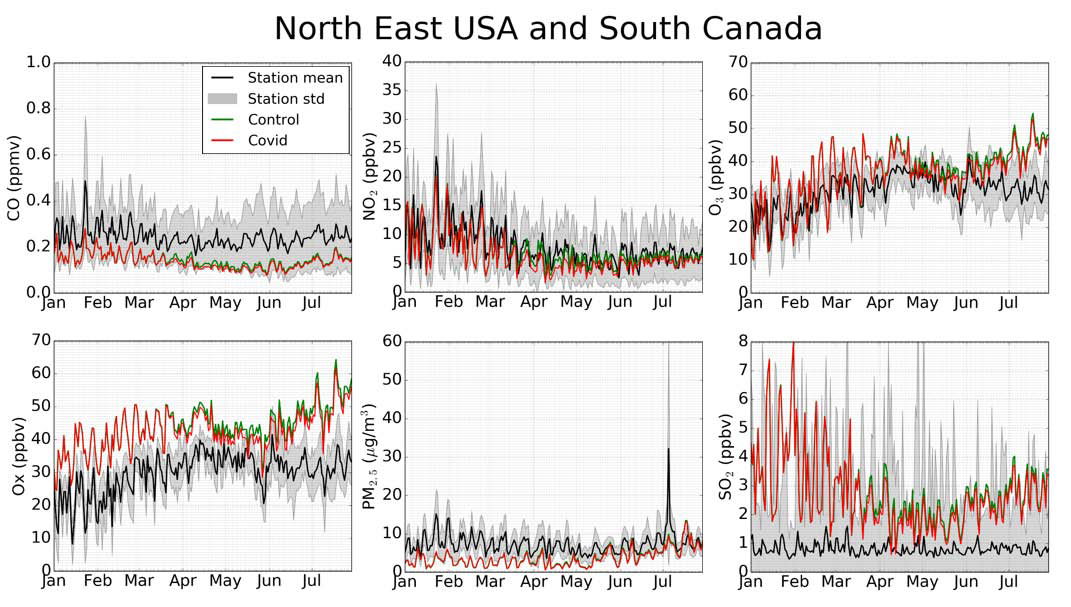


**Figure S4d.** Evolution for the period 1 January – 31 May 2020 of the surface concentration of NO_2_, CO, O_3_, Ox, PM_2.5_ and SO_2_ in Northeastern USA and South Canada. Black curve: measurements from monitoring stations. Green curve: model control case. Red curve: model case with emissions modified to account for the effect of the COVID-19 pandemic.


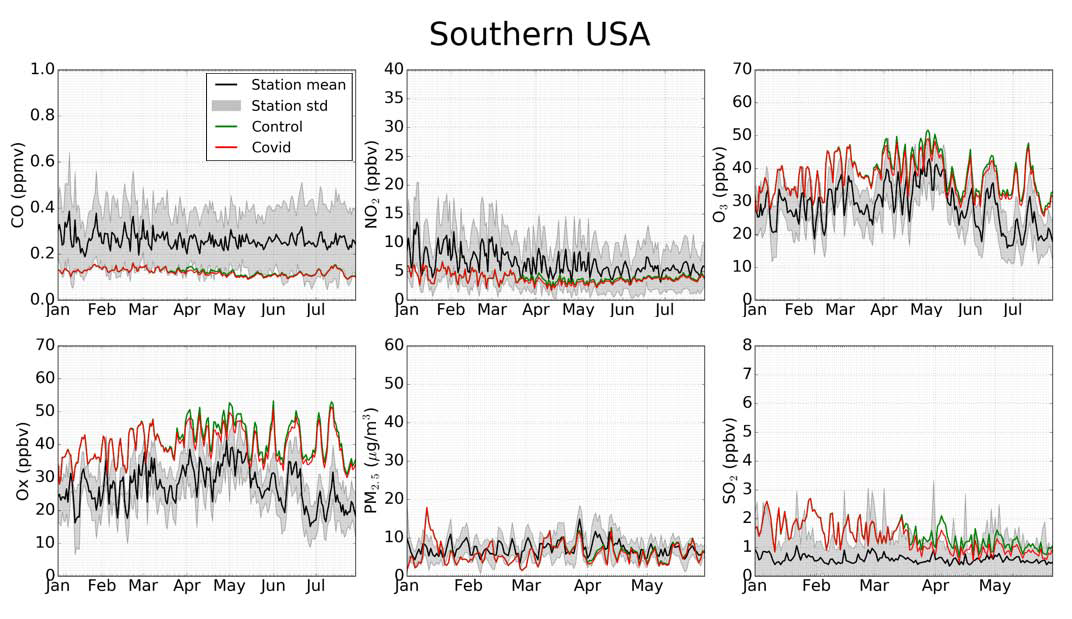


**Figure S4e.** Evolution for the period 1 January – 31 May 2020 of the surface concentration of NO_2_, CO, O_3_, PM_2.5_ and SO_2_ in Southern USA. Black curve: measurements from monitoring stations. Green curve: model control case. Red curve: model case with emissions modified to account for the effect of the COVID-19 pandemic.


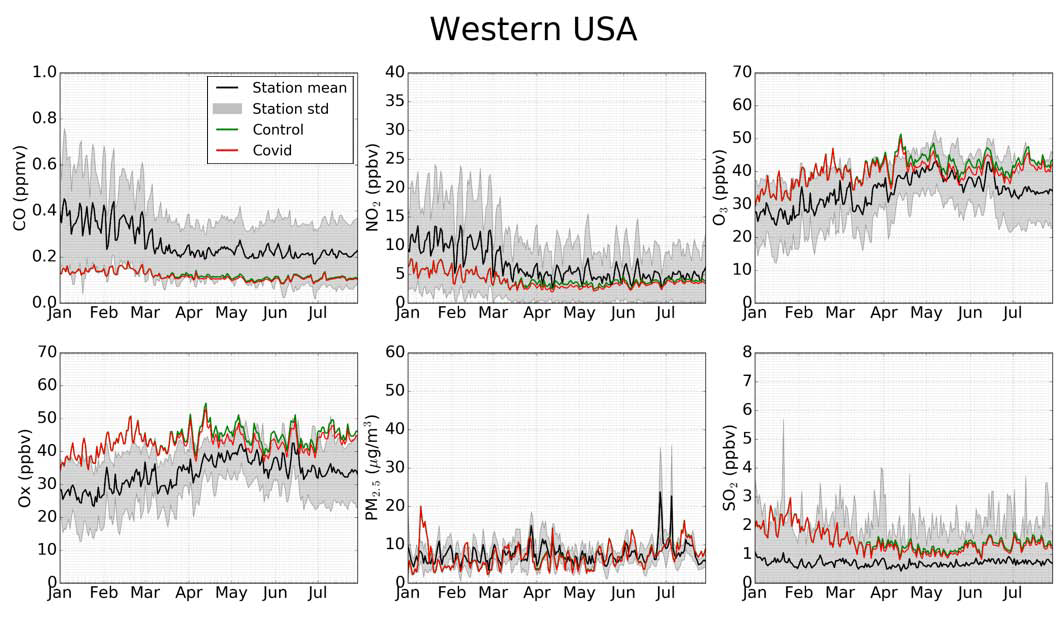


**Figure S4f.** Evolution for the period 1 January – 31 May 2020 of the surface concentration of NO_2_, CO, O_3_, SO_2_ and PM_2.5_ in Western USA. Black curve: measurements from monitoring stations. Green curve: model control case. Red curve: model case with emissions modified to account for the effect of the COVID-19 pandemic.

**
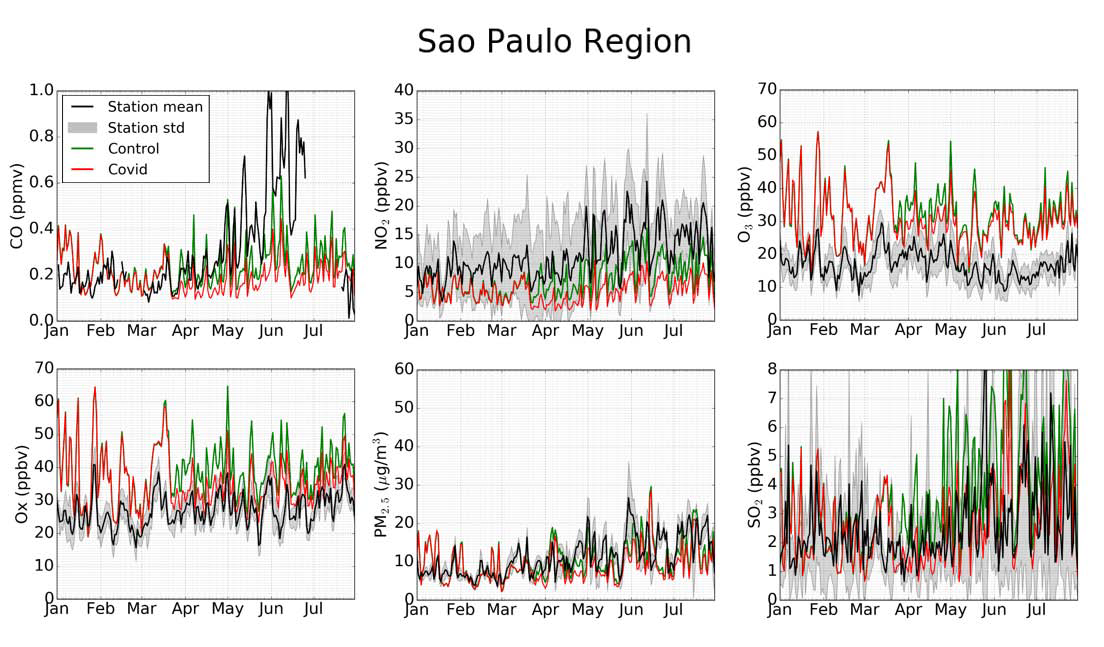
Figure S4g.** Evolution for the period 1 January – 31 July 2020 of the surface concentration of NO_2_, CO, O_3_, Ox, PM_2.5_ and SO_2_, in the region of Sao Paulo. Black curve: measurements from monitoring stations. Green curve: model control case. Red curve: model case with emissions modified to account for the effect of the COVID-19 pandemic.

Finally, an examination of the ozone concentration in several densely populated areas of Europe (Figure S4h), shows a good agreement between the model and the ground measurements, particularly near Berlin, Hamburg and Paris. In the area around London, Milan and Madrid, however, the model slightly overestimates the concentration of this gas in January, but the difference is reduced in the following months.


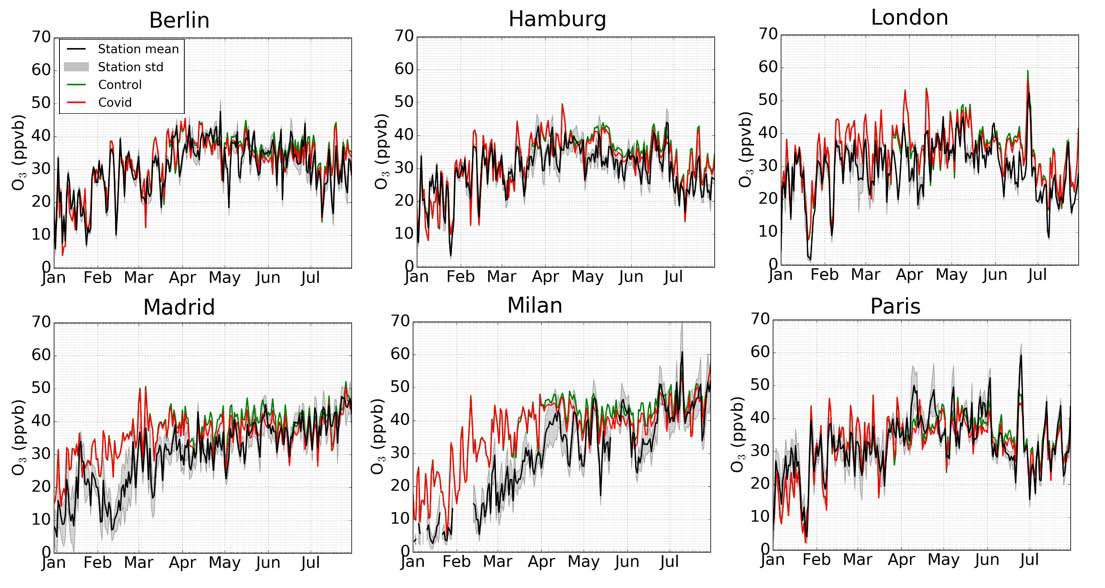


**Figure S4h.** Evolution for the period 1 January – 31 July 2020 of the surface concentration of ozone in the urban areas of Berlin, Hamburg, London, Madrid, Milan and Paris. Black curve: measurements from monitoring stations. Green curve: model baseline case. Red curve: model case with emissions modified to account for the effect of the COVID-19 pandemic.

**Text S5.** The following maps display the evolution of the montly mean changes in the surface mixing ratio of several species during the first months of 2020 relative to a baseline case with no COVID-19 related changes in the emissions, but with similar dynamics.


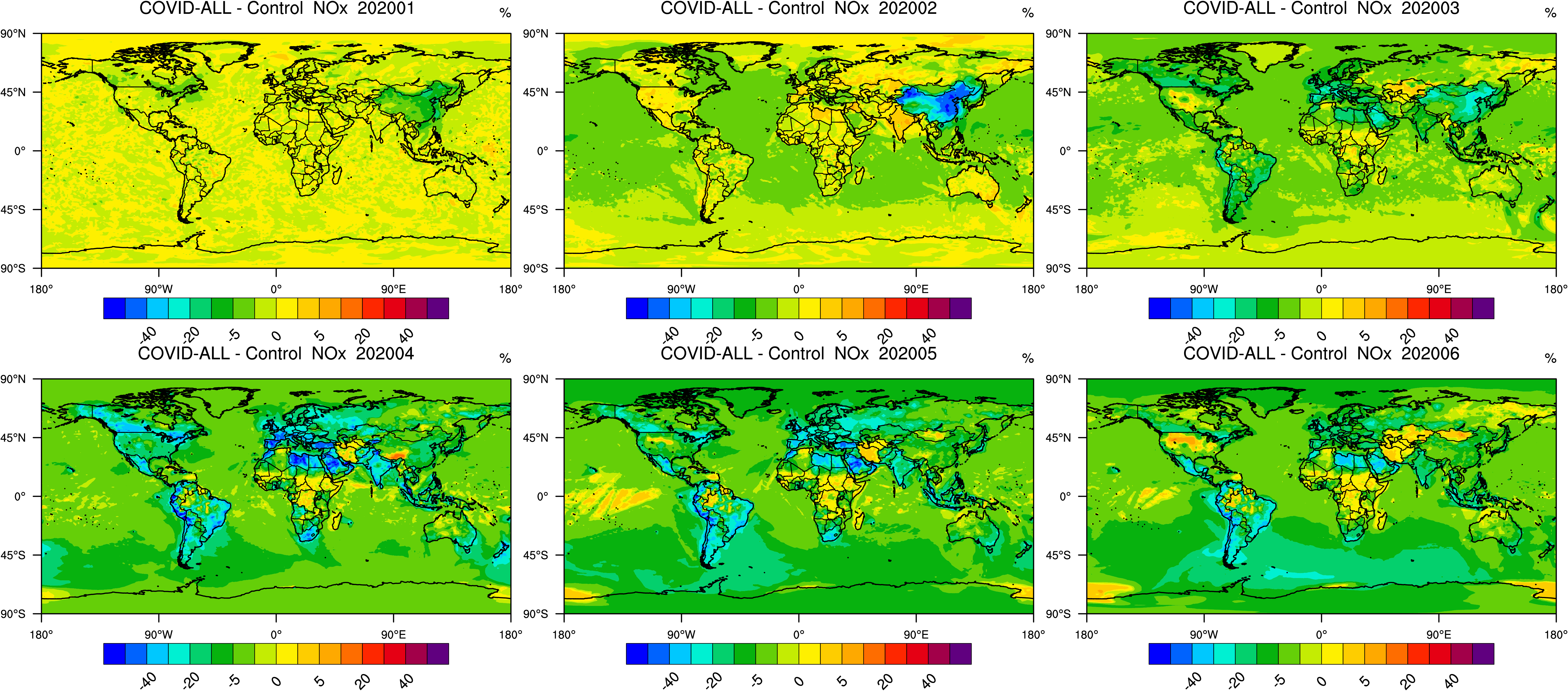


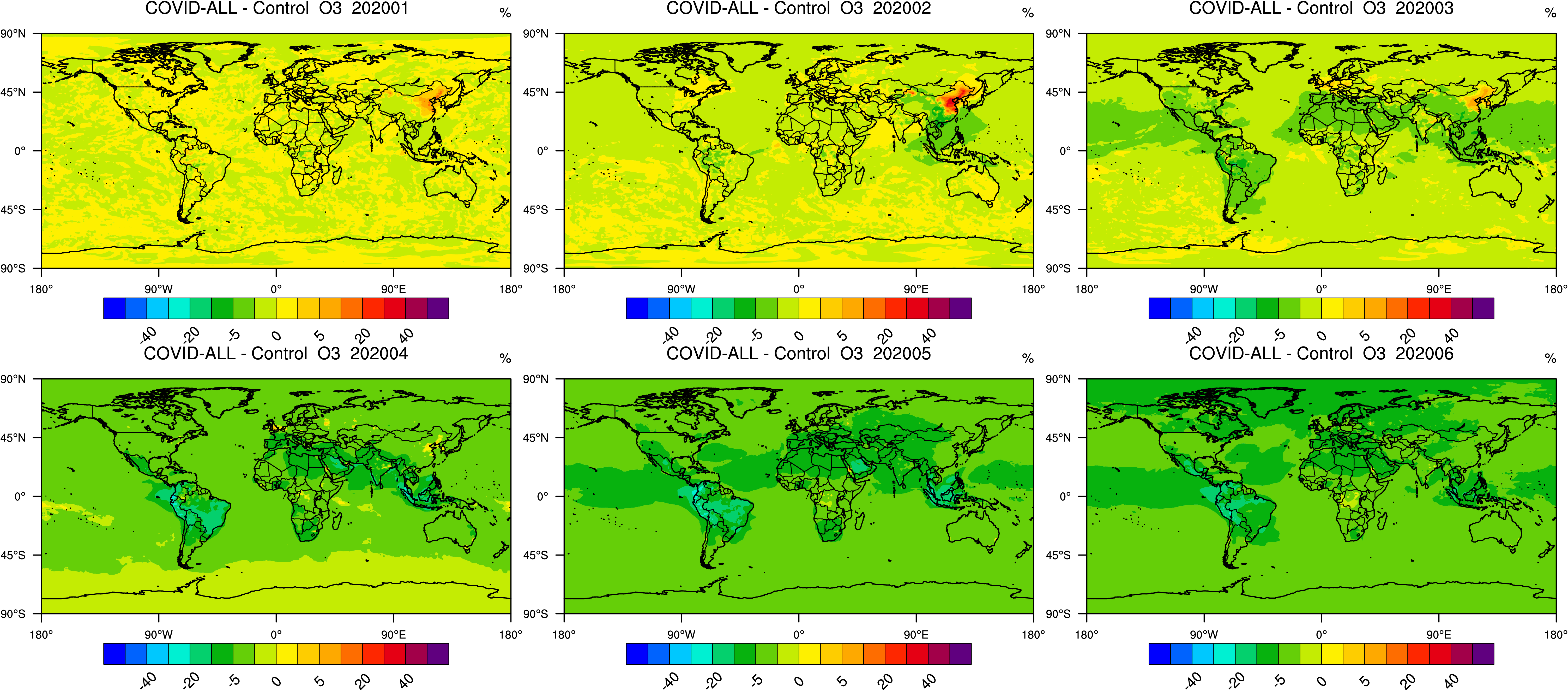


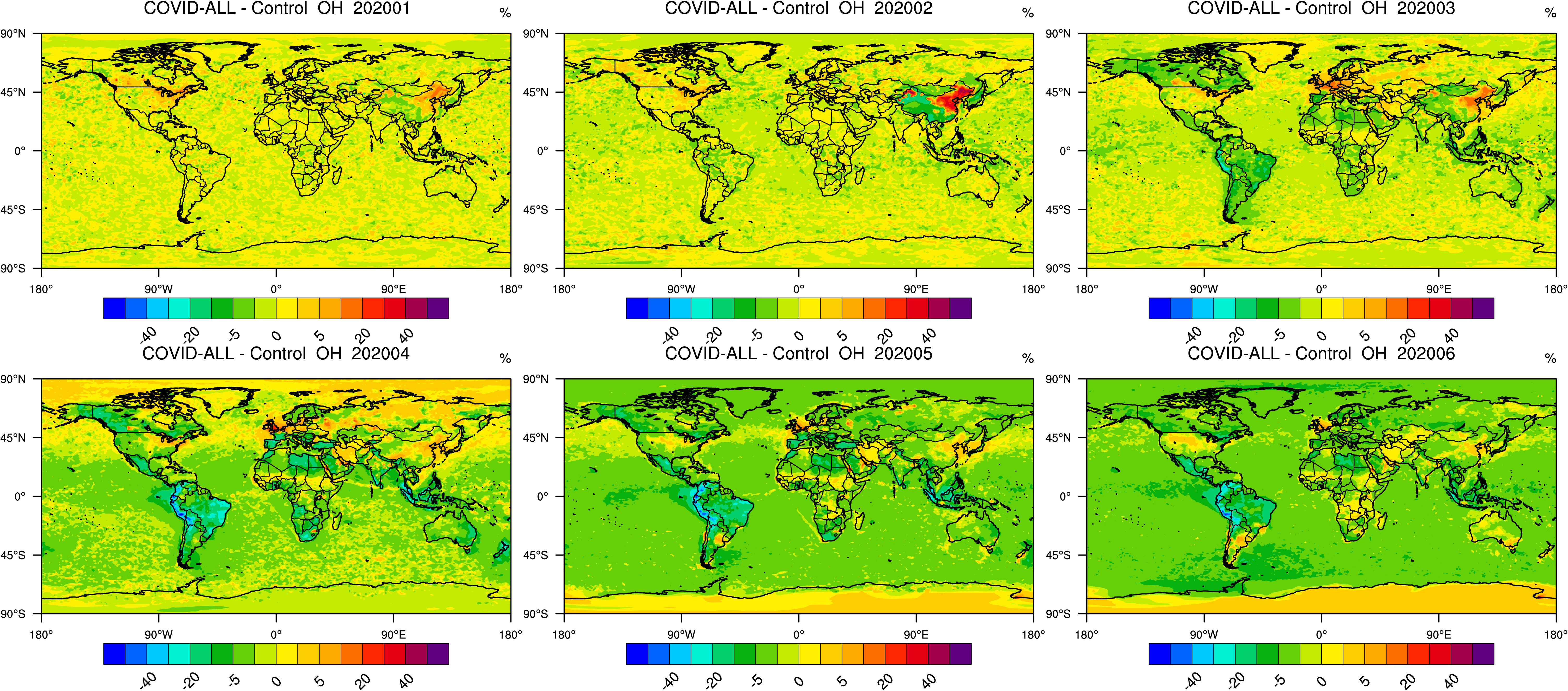


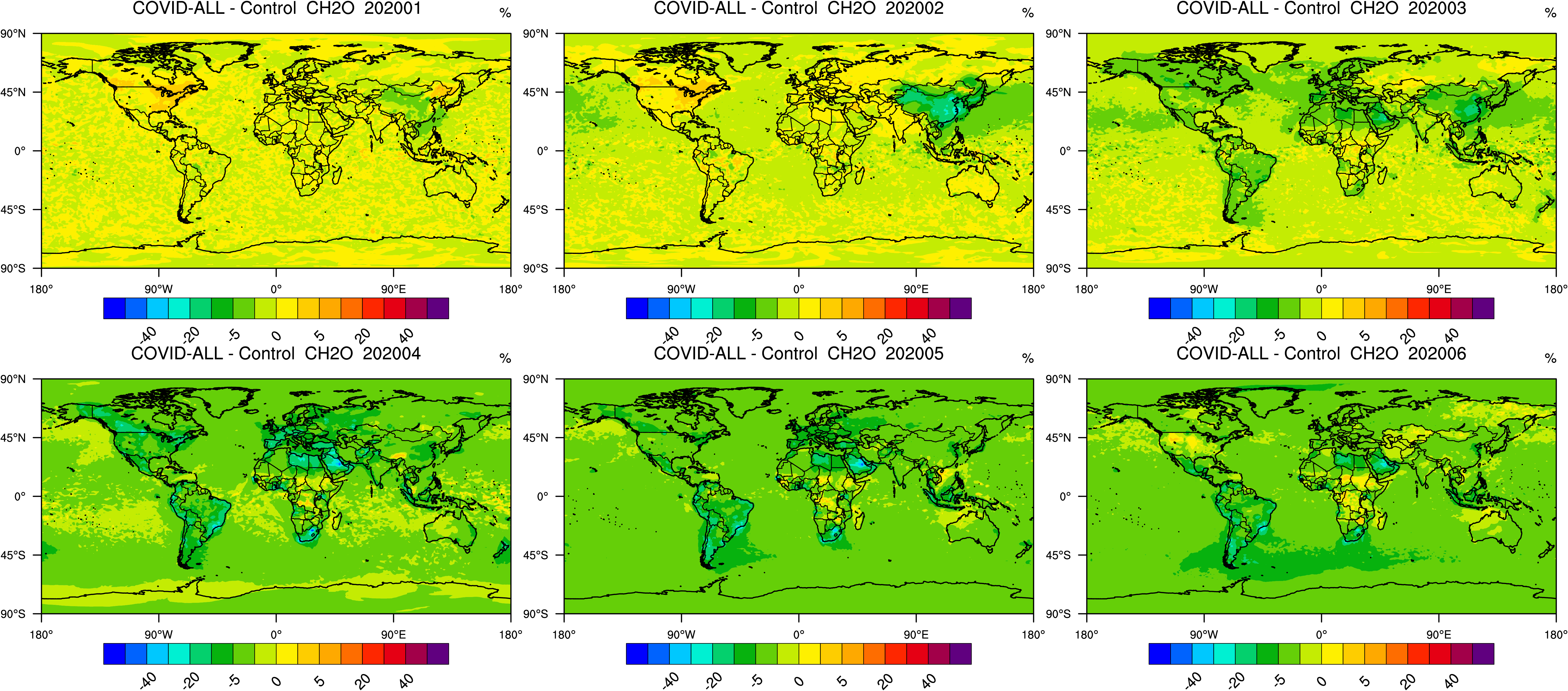


**
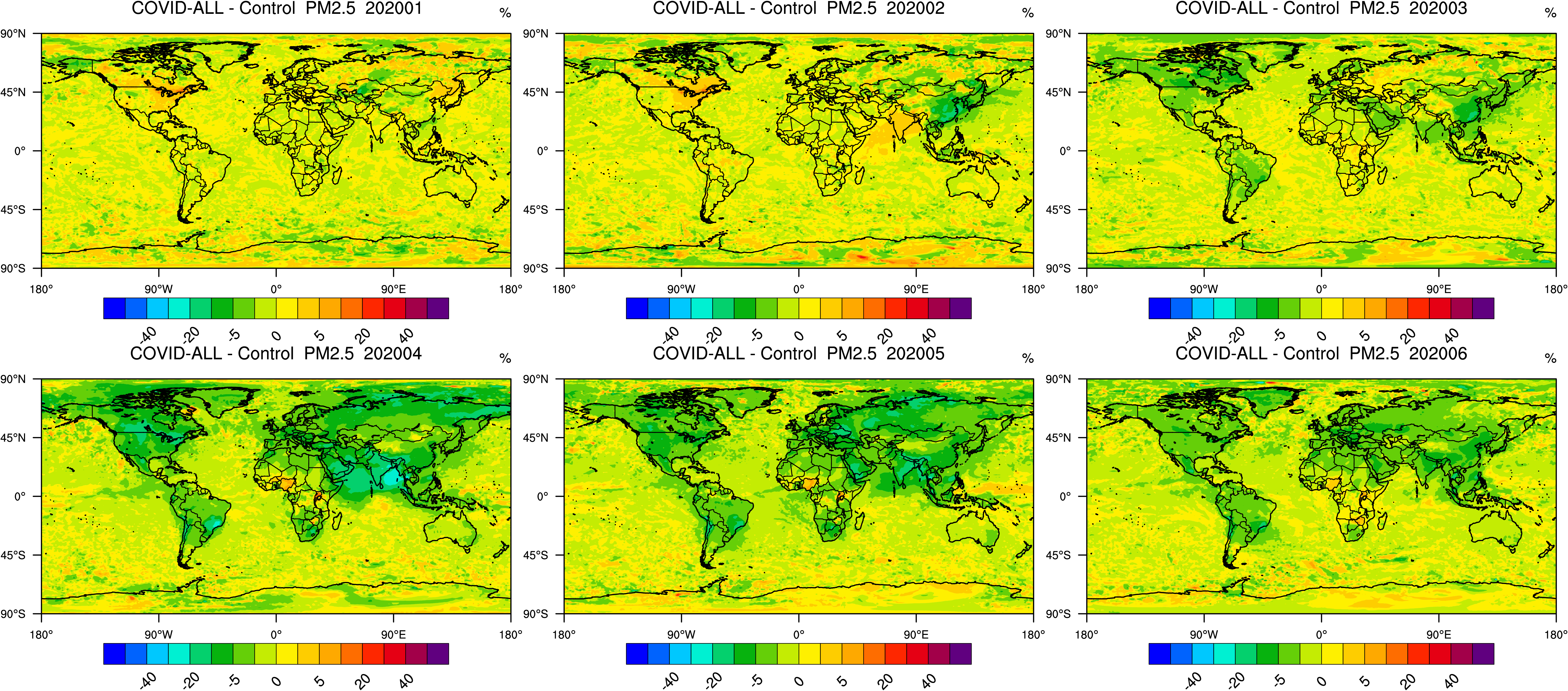
**

**Figure S5**. Evolution of the monthly mean change (percent) in the surface mixing ratio of NOx, ozone, OH, HCHO, and PM2.5 from January to May 2020 in response to the reduction in the anthropogenic emissions of primary pollutants resulting from the COVID-19 pandemic.

**Text S6.** Changes in the global OH concentration in February 2020 relative to a case with baseline emissions and same dynamics.


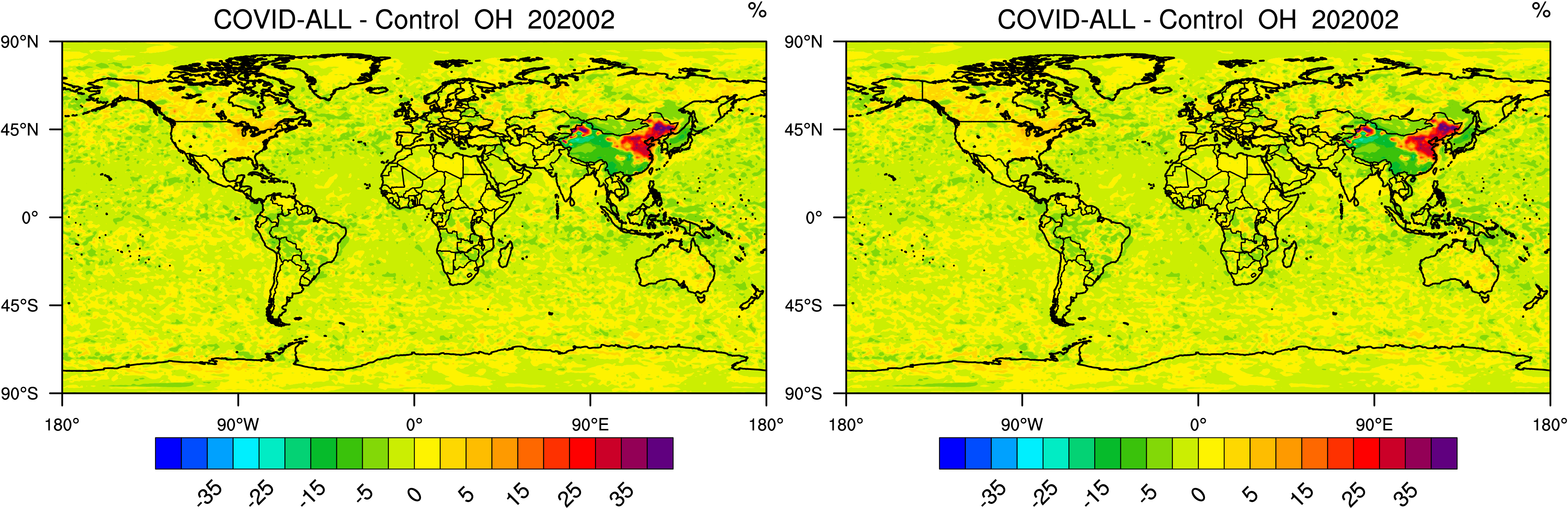

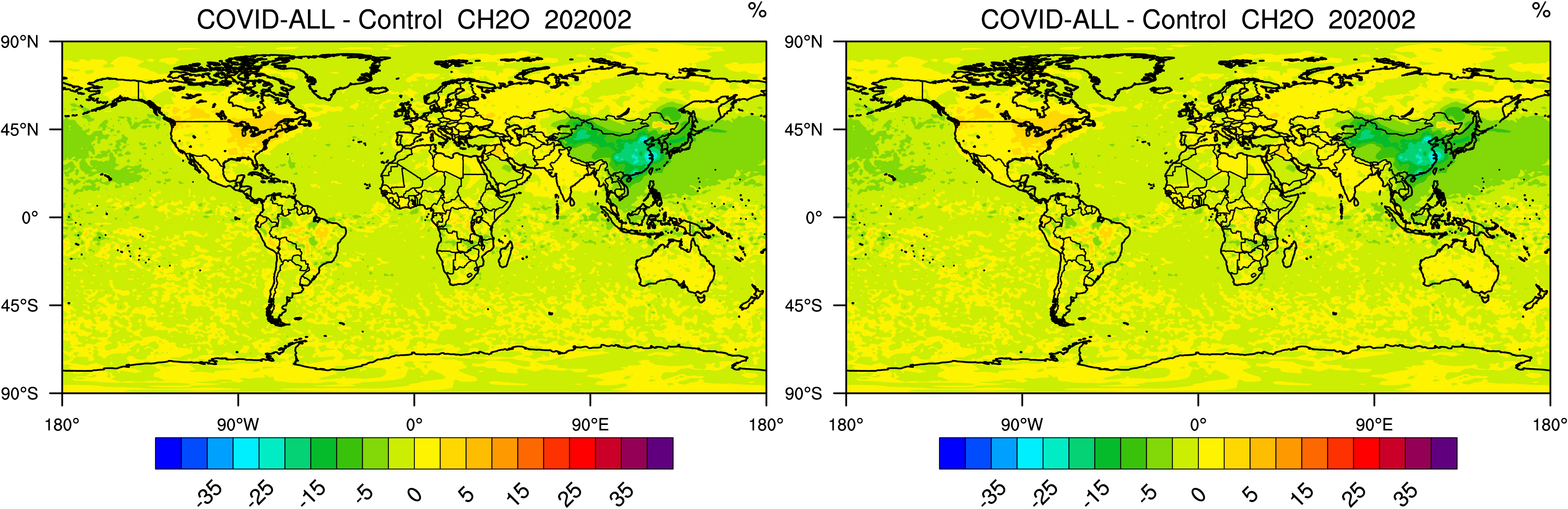


**Figure S6.** Relative change (percent) in February 2020 in the global monthly mean concentration of (from top to bottom) of OH and H_2_CO resulting from the change to the adopted surface emissions of primary pollutants during the COVID-19 pandemic period.

**Text S7.** Global response of Secondary Organic aerosol concentrations due to the change in emissions during the COVID-10 pandemic**.**


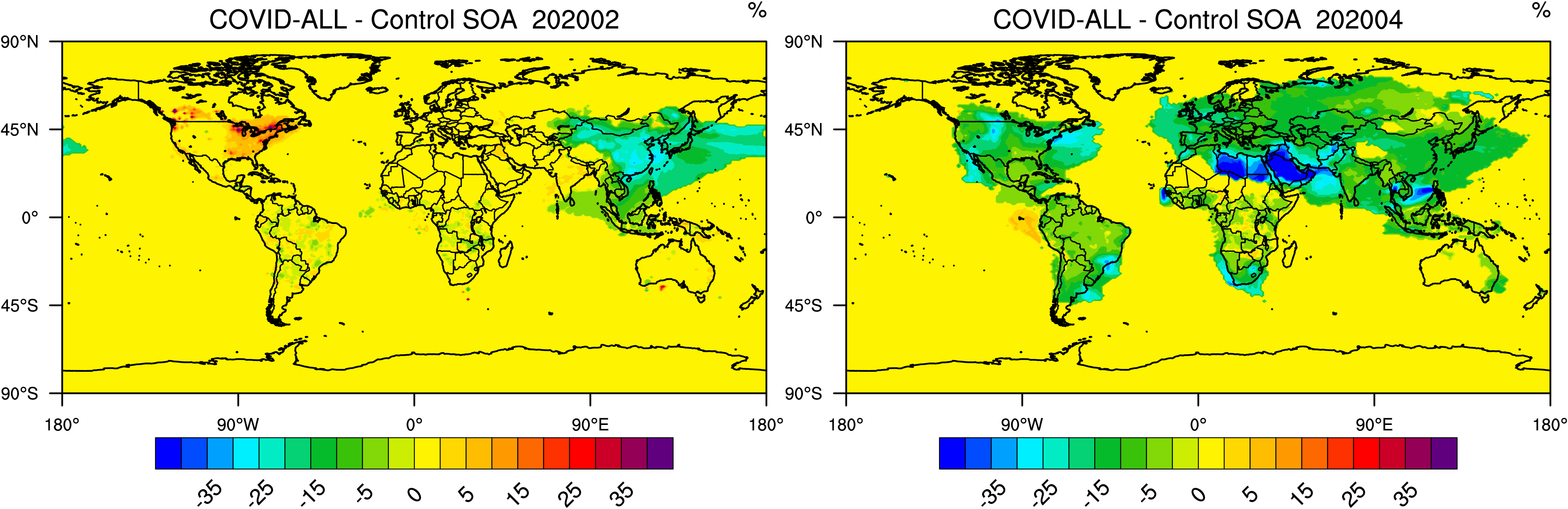


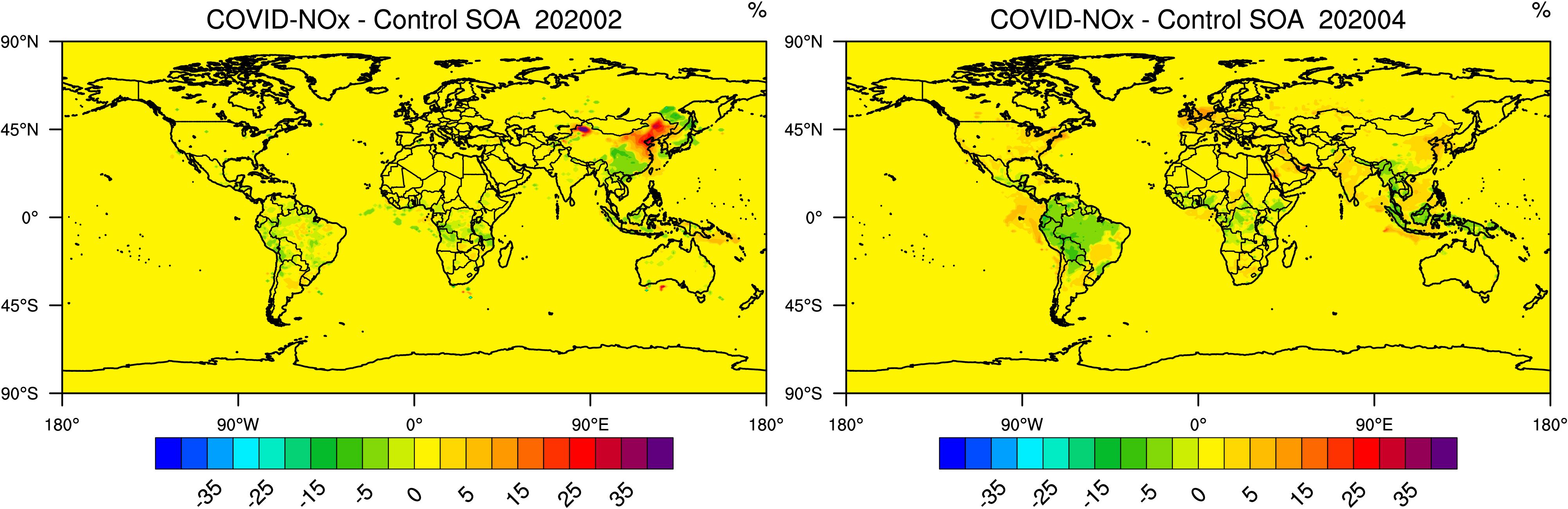


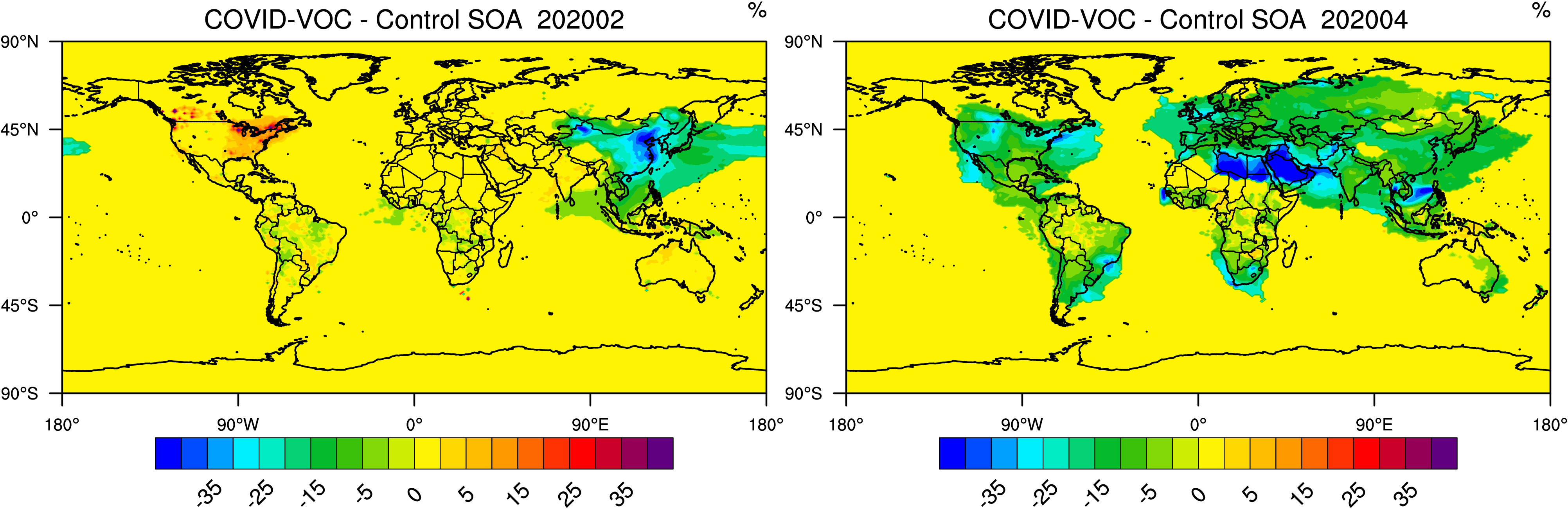


Figure S7. Relative change in the concentration of secondary organic aerosols (SOA) resulting from reduced emissions of primary pollutants during the COVID-19 pandemic for February (left panels) and April 2020 (right panels). Upper panels: all emissions reduced; middle panels: reduction of NOx emissions only; lower panels: reduction in VOC and CO emissions only.

Text S8. Radical sources, specifically the sources of HOx, determine the sensitivity of ozone to NOx and VOCs. Li et al (2021) have shown that the photolysis of formaldehyde (H_2_CO) constitutes the largest sources of HOx during winter in the Northern Plain of China, specifically during haze events. Nitrous acid (HONO) is another significant source. We represent here the calculated monthly mean source of HOx (February and July) resulting from the photolysis of H_2_CO and HONO during COVID-19 situations and their relative importance. Our model suggests that the production by CH_2_O photolysis dominates in most areas of Asia, except in northern China where the contribution of the HONO photolysis is largest. The size of the geographical area where HONO photolysis dominates is considerably largest in winter.


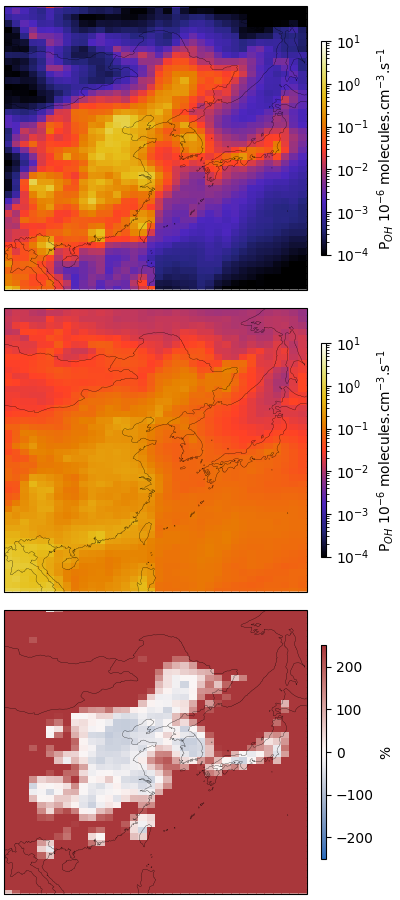

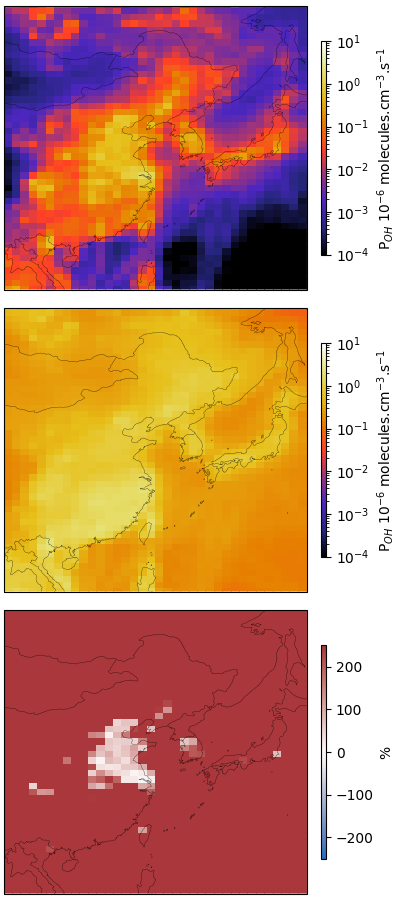


Figure S8. Top and middle panels: Production rate (10^-6^ molecules cm^-2^ s^-1^) of odd hydrogen radicals (HOx) at the surface by the photolysis of HONO [*P*_HONO_(HOx); top panel] and H_2_CO [*P*_H2CO_(HOx); middle panel]. The lower panel shows the ratio 100 x (*P*_H2CO_ (HOx) - *P*_HONO_ (HOx))/*P*_HONO_(HOx), and highlights the relative importance of the two HOx production processes.

**Text S9**. Changes in the monthly mean surface concentration of several species in Asia during February 2020 relative to a baseline case.


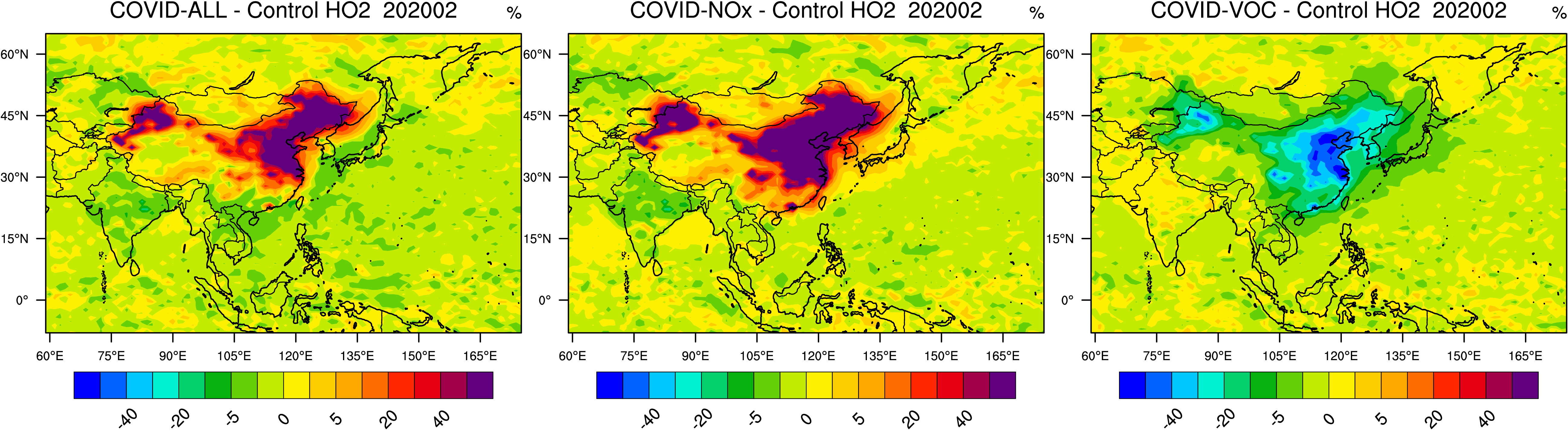


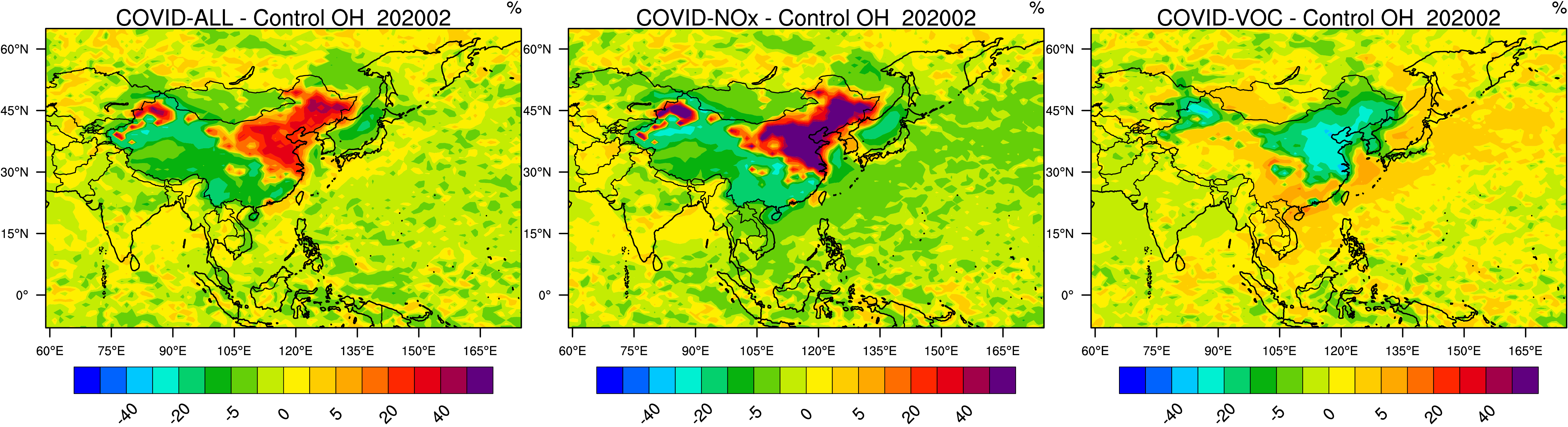


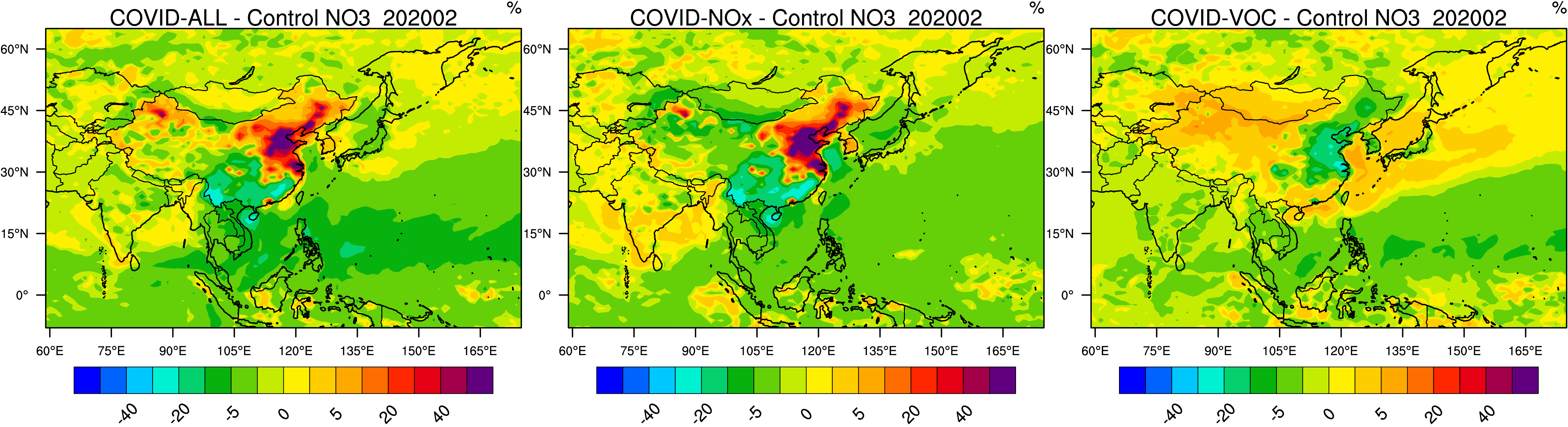


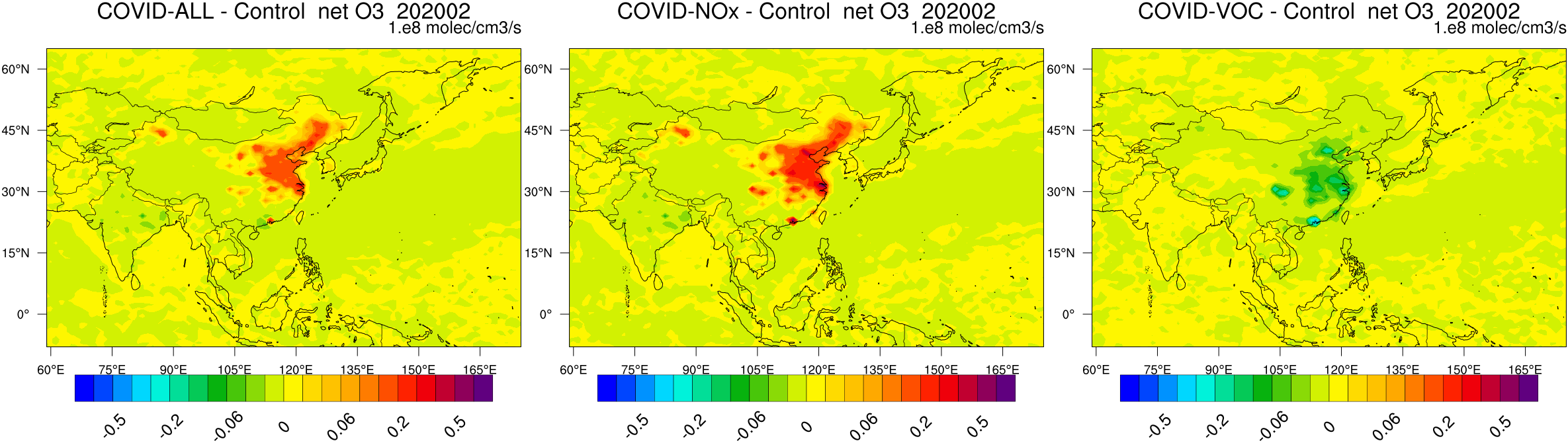


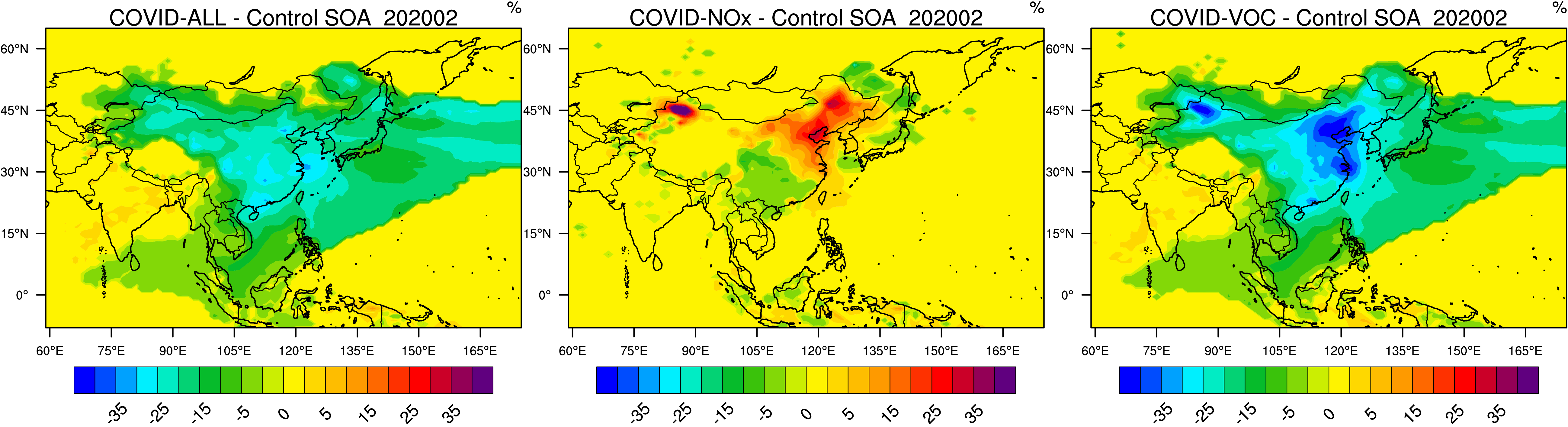


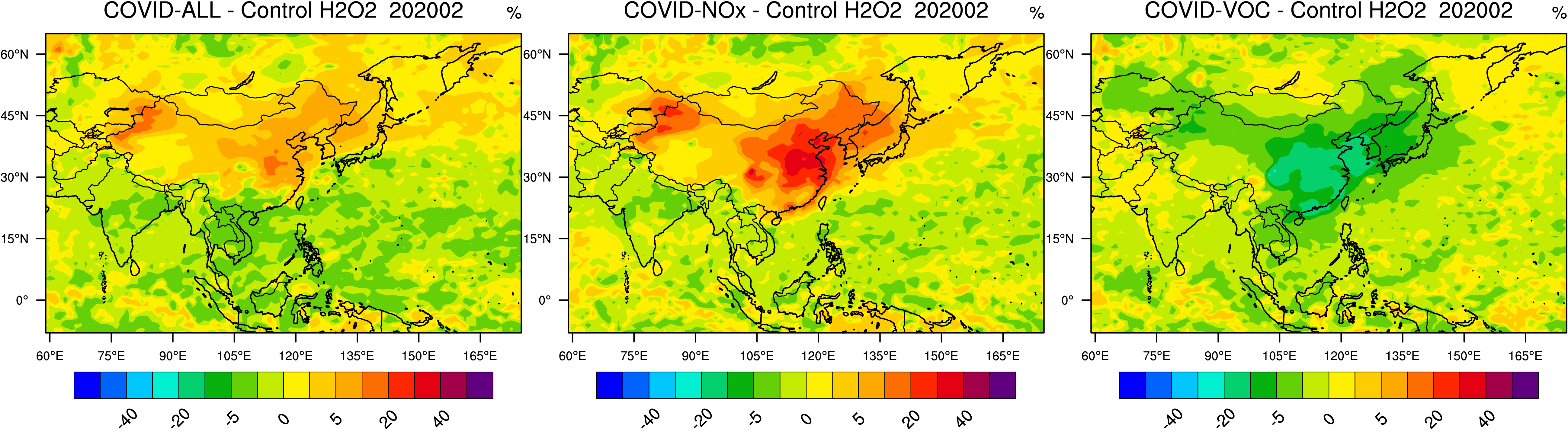


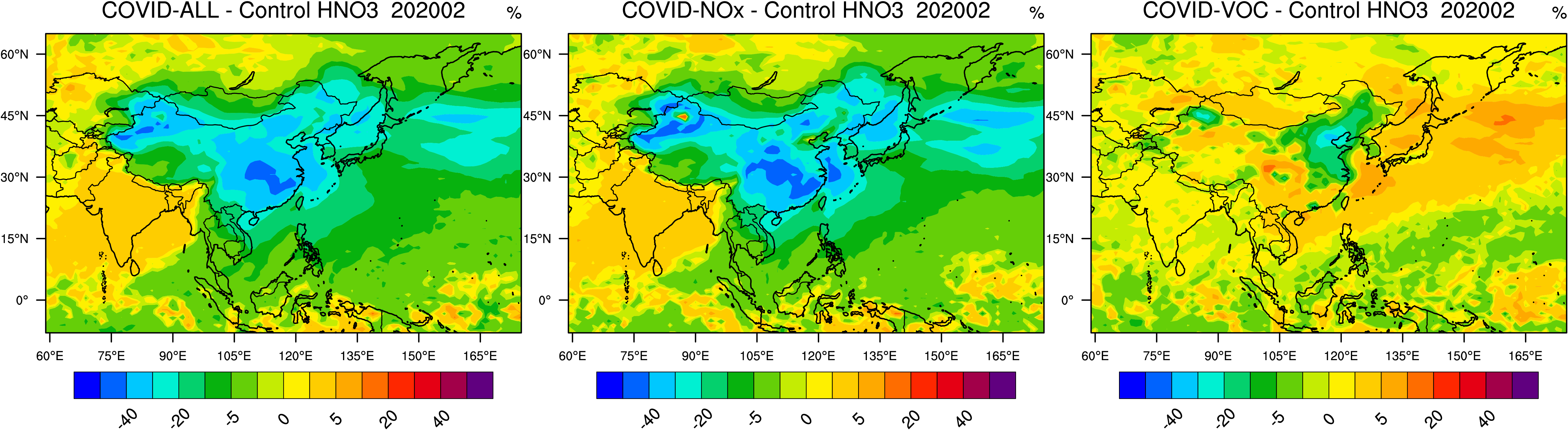


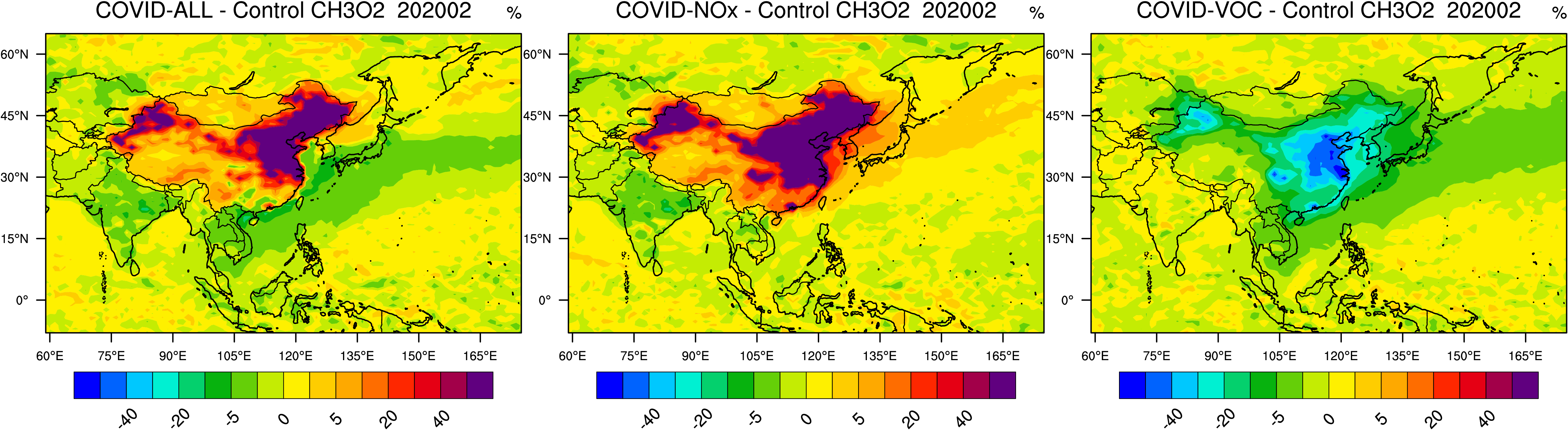


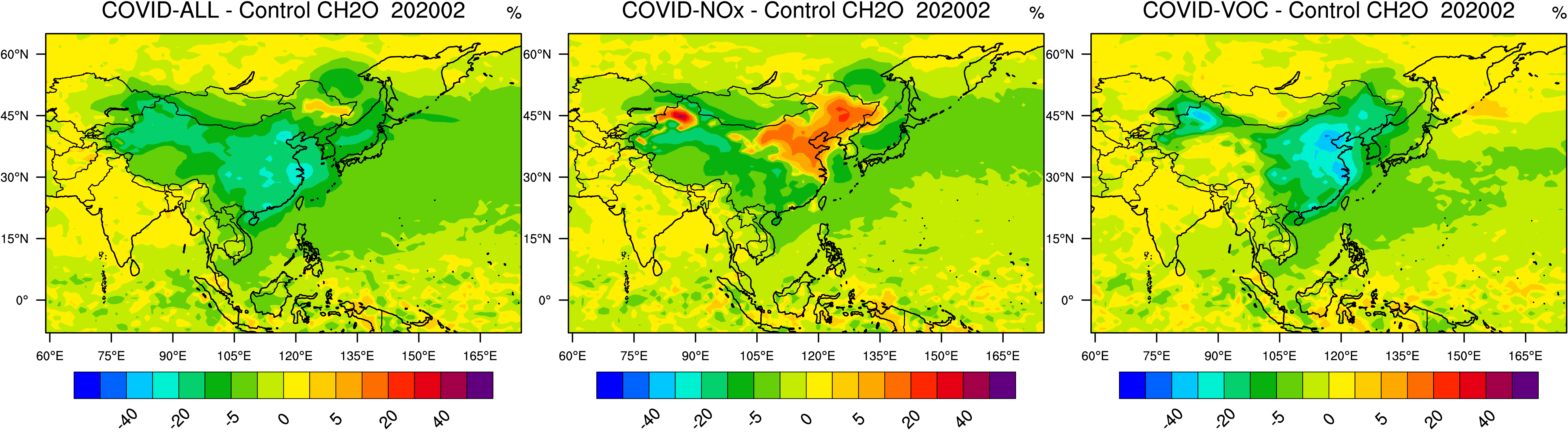


Figure S9. Percentage change in several chemical variables in China in response to reduced emissions of primary pollutants in February 2020 during the COVID-19 pandemic. From the top panels to the bottom panels: HO_2_, OH, NO_3_, net ozone production (cm^-3^ s^-1^), SOA H_2_O_2_, HNO_3_, HO_2_, CH_3_O_2_, HCHO. Left column: reduction in all emissions; center panel: reduction in NOx emissions only; right panel: reduction in VOC and CO emissions only.

**Text S10**. Effect of meteorological variability on the response of NOx and ozone in Asia to the COVID-related changes in emissions.

In order to provide some insight on the relative forcing effects of the emission reduction during the pandemic and of the meteorological variability, we provide in Figure S9 an estimate of the ozone anomaly generated by weather dynamics and by the combined effects of the two forcing factors. Wang and Zhang (2020) provide a detailed assessment of the effects of meteorological elements during the pandemic period. Our model simulations as nudged towards the MERRA-2 meteorology show that during February 2020 and relative to our 5-year “pseudo-climatology”, Eastern China was abnormally warm by 1.5 to 2.5 K and subject to high cloud fraction; northern China was 2-4 K warmer with cloud fraction lower relative to the previous 5-year average. During this month, ozone anomalies associated with meteorological variability were dominant in the tropical regions south of China, but were relatively weak on the Chinese mainland. Abnormally low ozone was found along the border between China and Mongolia related to the abnormally high NO_2_ concentration calculated during February 2020. The increase in the monthly mean ozone concentration in the North China Plain (up to 5%) predicted by the model in response to meteorological anomalies adds to the ozone perturbation caused by the reduction in emissions. Our simulations suggest that chemical disturbances rather than meteorological anomalies explain the ozone concentration increase in the North China Plain during February 2020. Shorter time fluctuations linked to specific weather conditions should be considered in a finer analysis to explain, for example, the acute air pollution episodes reported in several urban areas during January and February 2020 (Wang et al., 2020). In southern China, where the perturbed chemistry tended to reduce ozone, a small positive anomaly is visible along the South China Sea. The change resulting from the two simultaneous effects is however negative except in the urban zone of Guangzhou/Hong Kong/Macao. In short, the enhancement in the level of oxidants in the North China Plain appears to be primarily a direct consequence of the reduction on chemical emissions triggered by the pandemic, but could have been facilitated by unfavorable weather conditions.


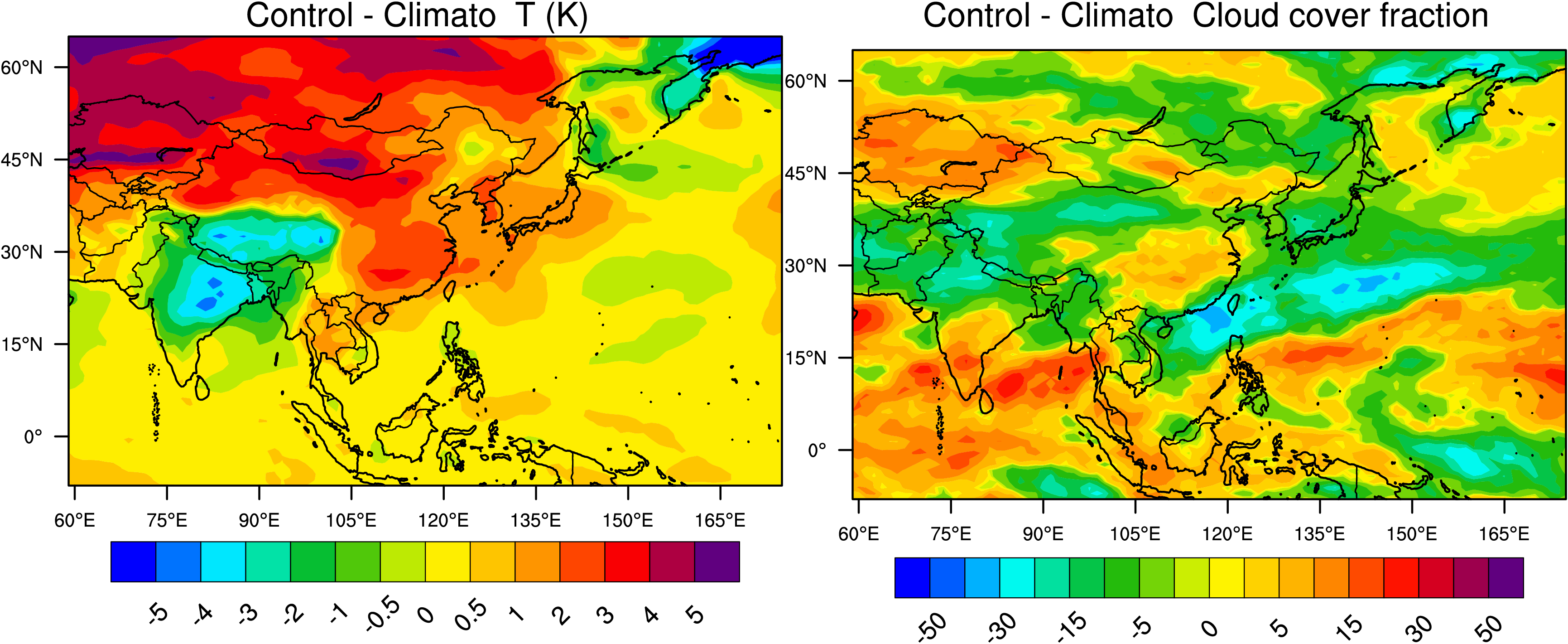


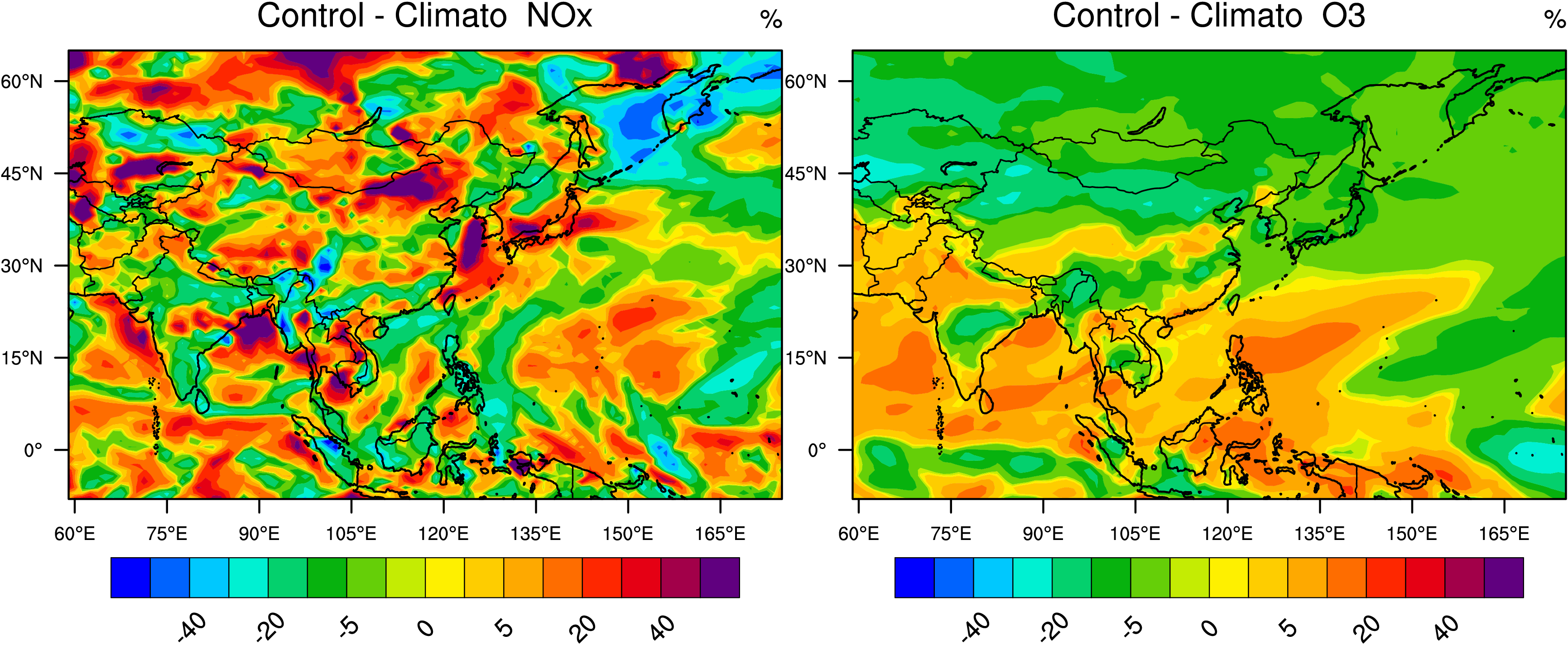


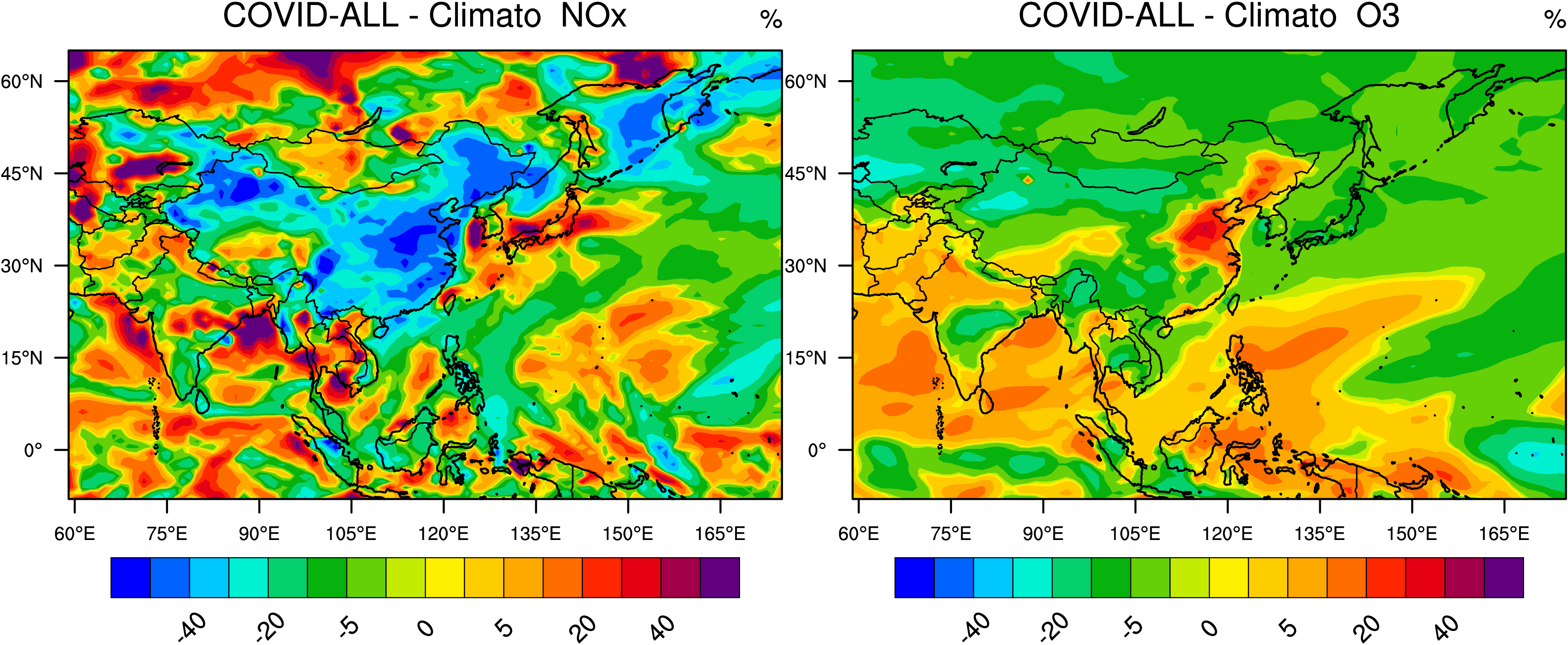


**Figure S10**. Changes in 2020 variables relative to a 5-year climatology: Upper panel: anomaly in temperature (Kelvin; left) and cloud cover fraction (right). Middle panel: change in NOx (percent; left) and ozone (percent; right) for the CONTROL minus CLIMATO case (effect of meteorological anomaly only). Lower panel: same as middle panel but for the COVID-ALL minus CLIMATO-case (effect of emission changes due to COVID and meteorological anomaly).

**Text S11**. Changes in the monthly mean surface concentration of several species in Europe during 15 March to 15 April 2020 relative to a baseline case.


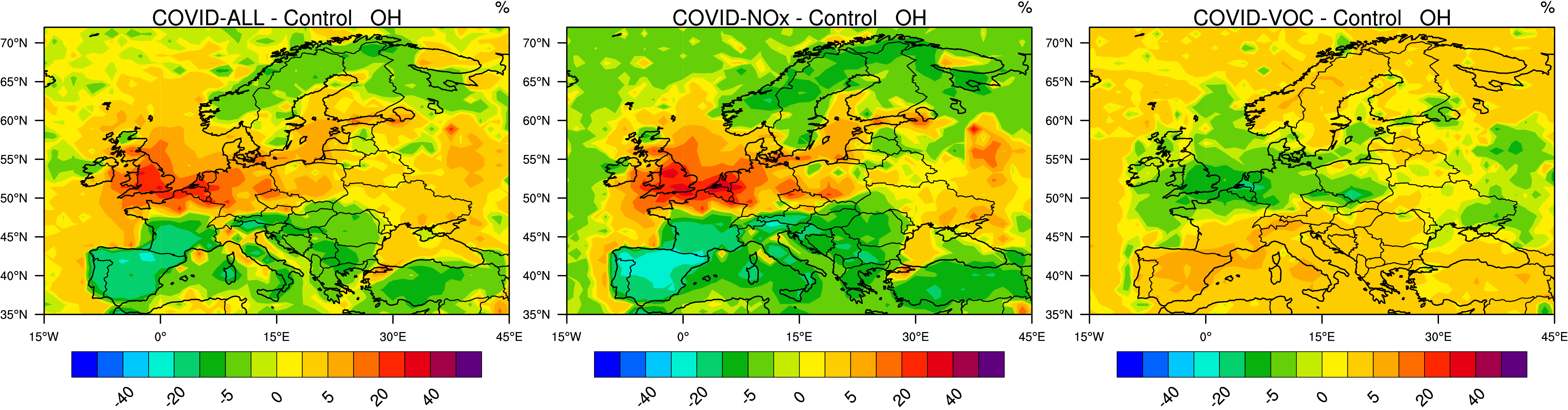


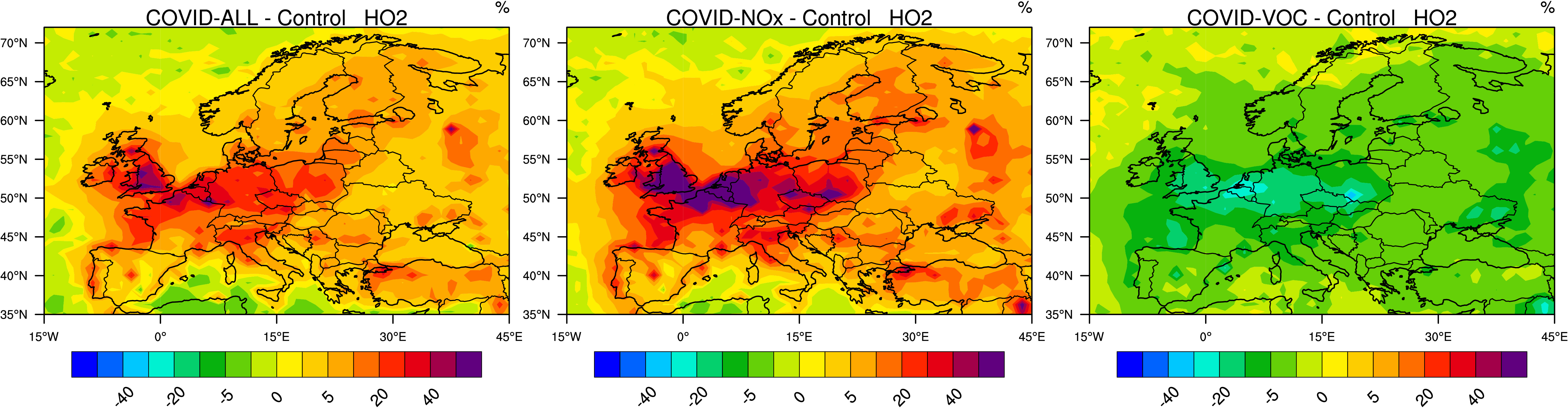


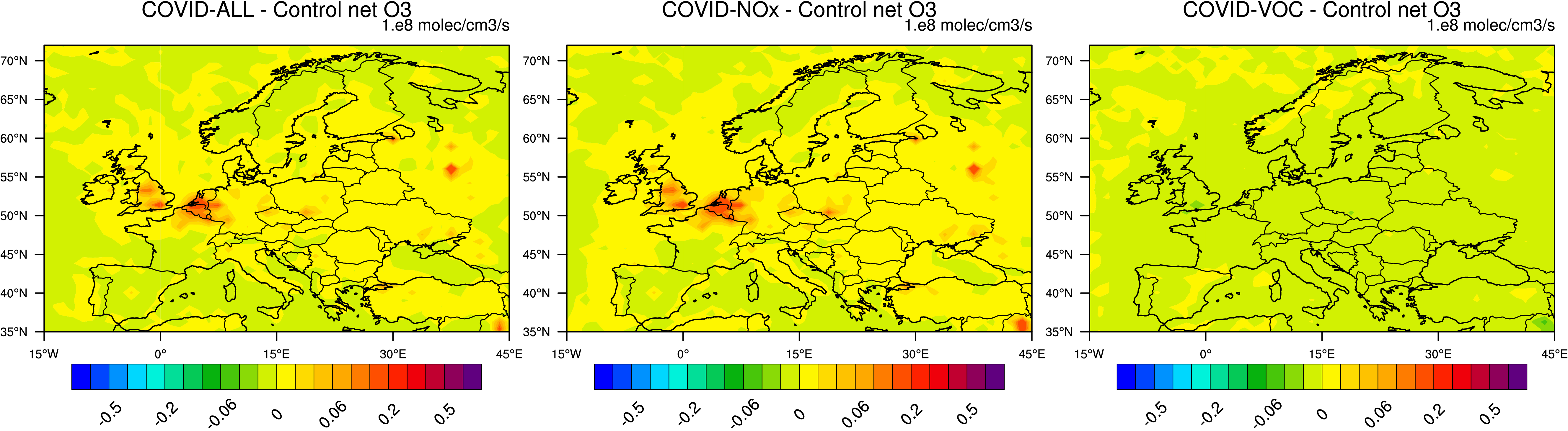


Figure S11. Percentage change in the surface concentration and in the net ozone production across Europe in response to the emissions of primary pollutants adjusted for the COVID-19 pandemic period of 15 March-14 April 2020. From top to bottom: NOx, OH, HO_2_, net ozone production (cm^-3^ s^-1^), ozone concentrations. Left column: reduction in all emissions; center panel: reduction in NOx emissions only; right panel: reduction in VOC and CO emissions only.

**Text S12**. Response to the monthly mean surface concentration of selected species in North America during the period 15 March-15 April 2020 relative to the baseline simulation.


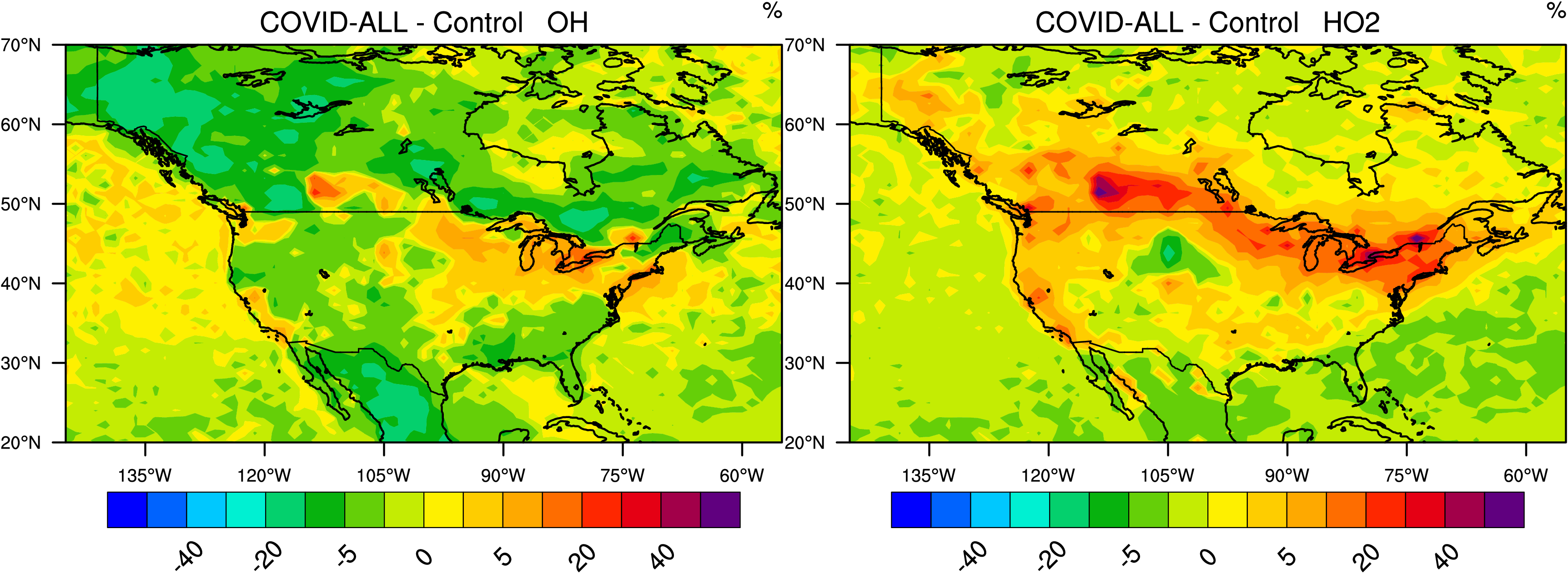


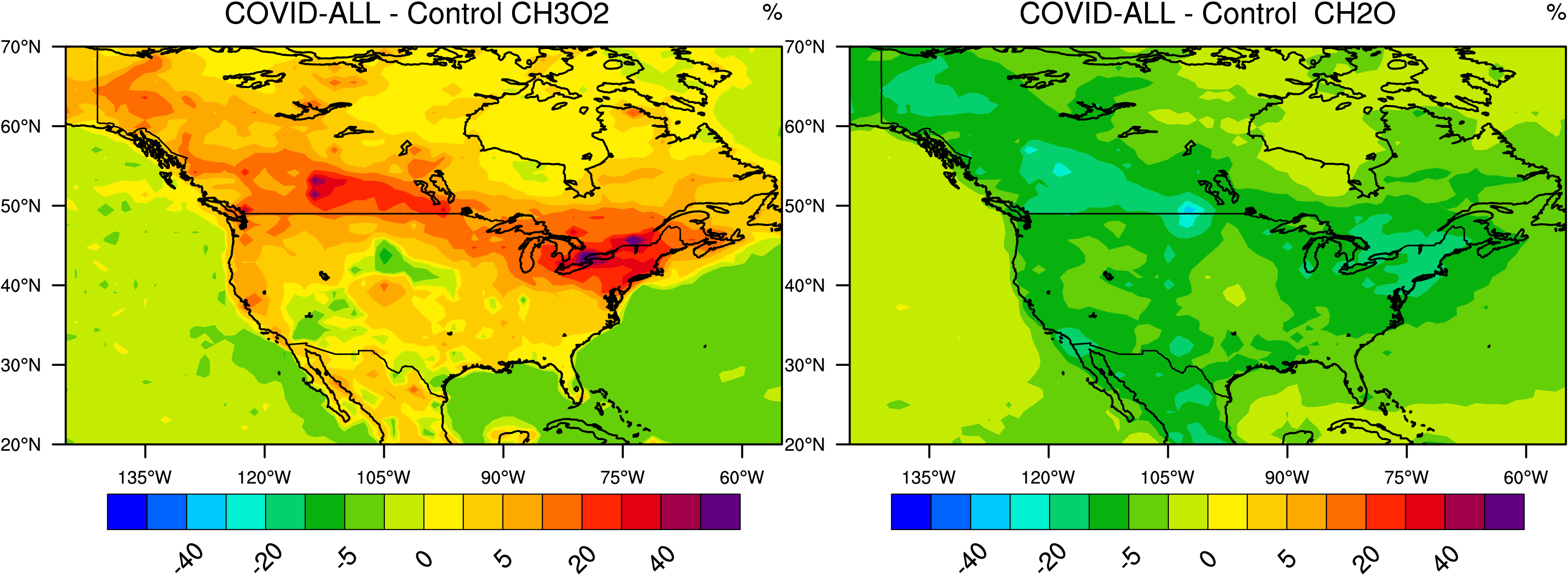


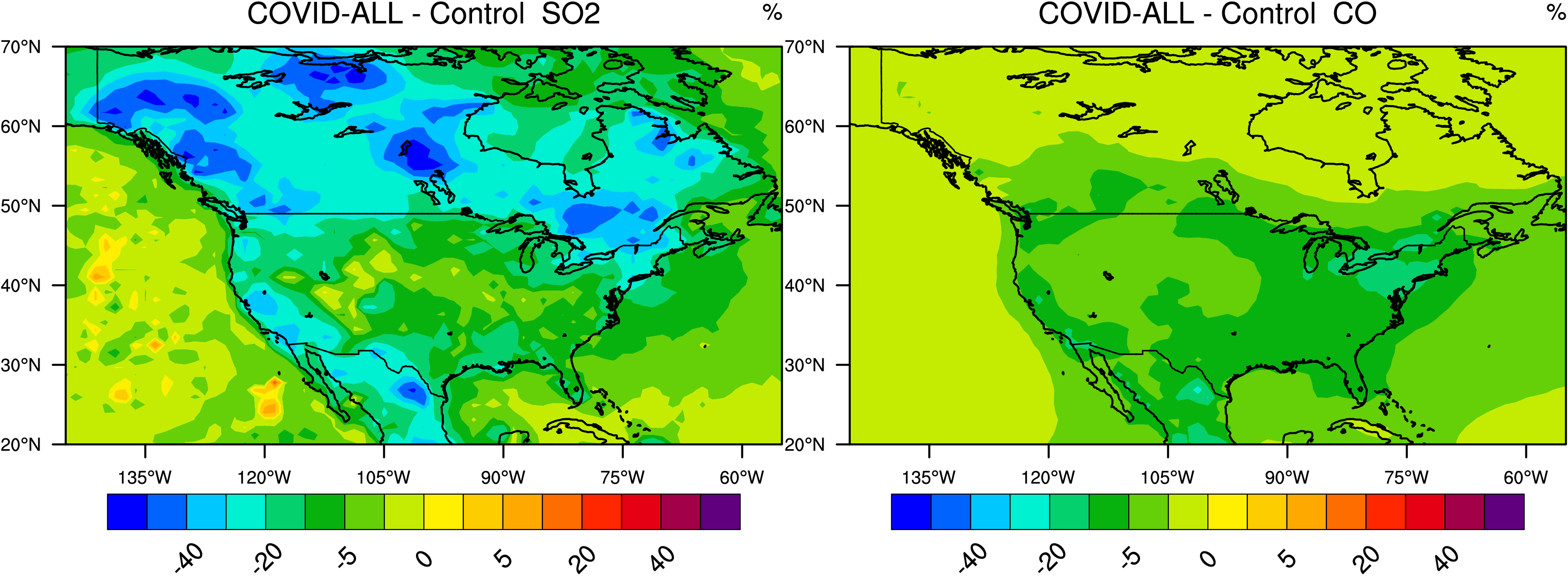


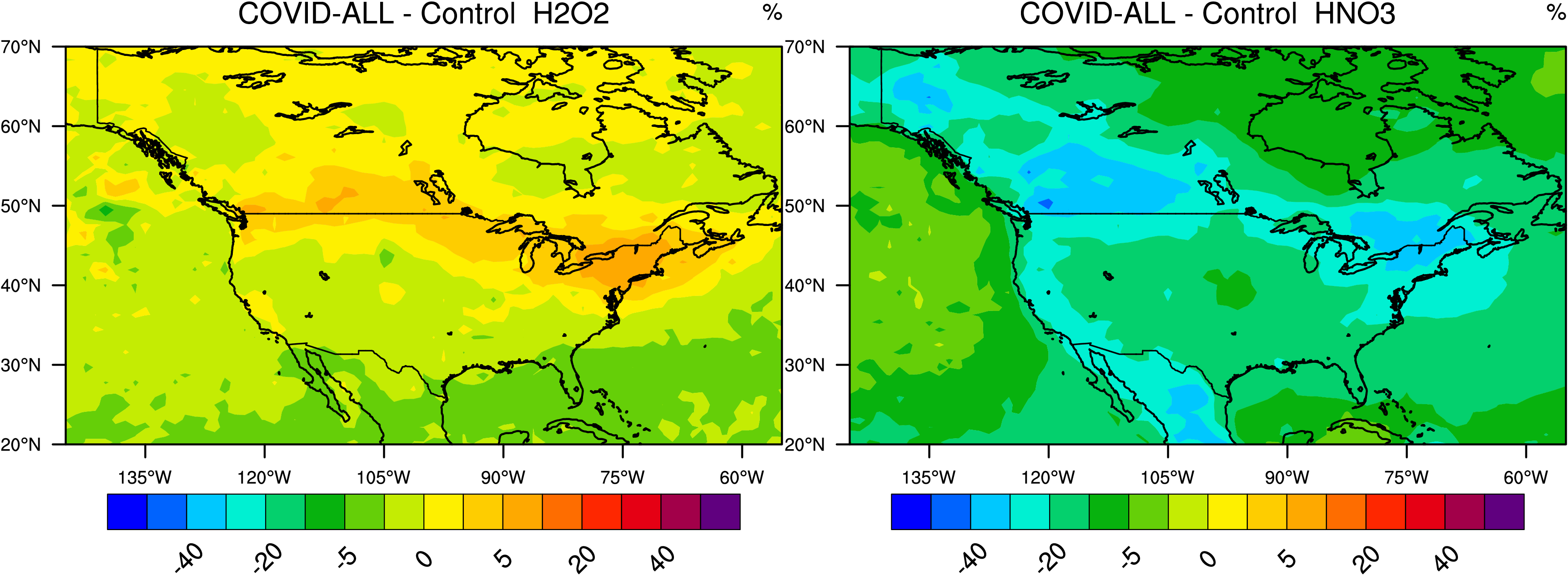


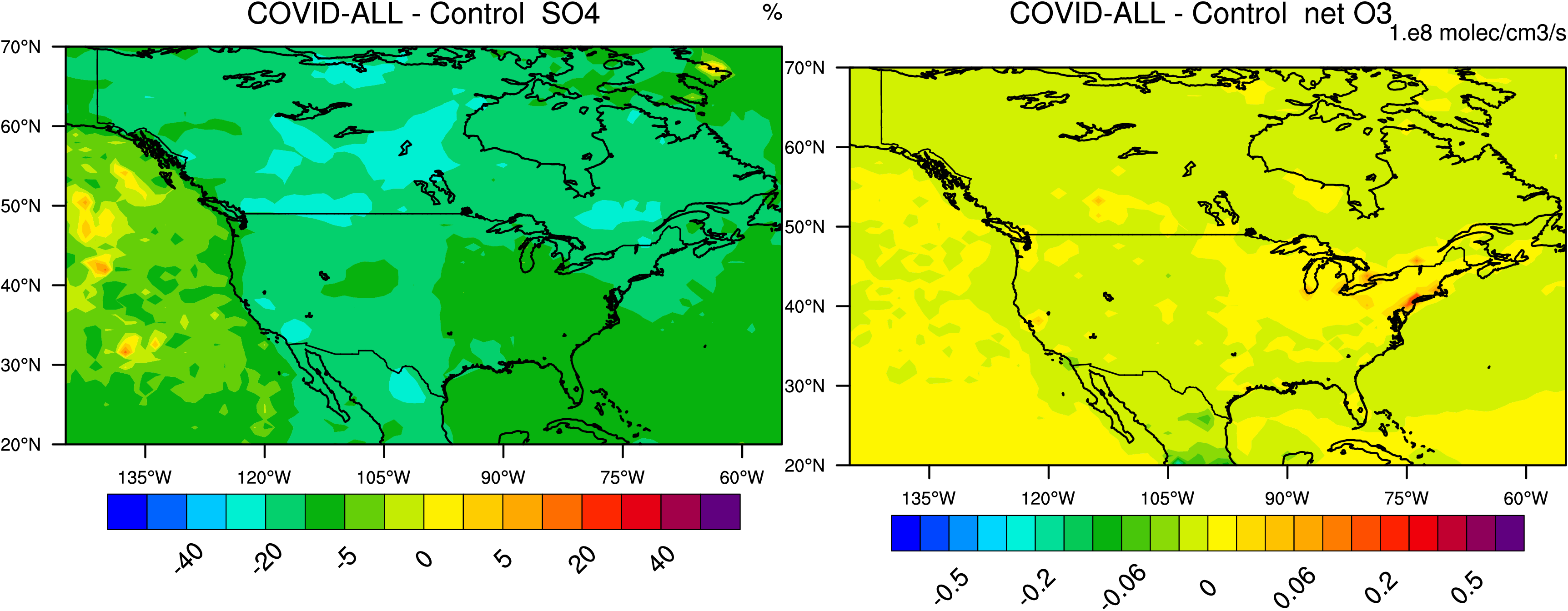
Figure S12. Change (from the top left panel to the bottom left panel) in the surface concentrations of OH, HO_2_, CH_3_O_2_, CH_2_O), SO_2_, CO, H_2_O_2_, HNO_3_, and SO_4_ [percent] and in the net ozone production rate [cm^-3^ s^-1^] in North America in response to reduced emissions of primary pollutants during the period 15 March - 14 April 2020 during the COVID-19 pandemic.

**Text S13**. Response to the monthly mean surface concentration of OH and HO_2_ in South America during the period 15 March-15 April 2020 relative to a baseline simulation.

**
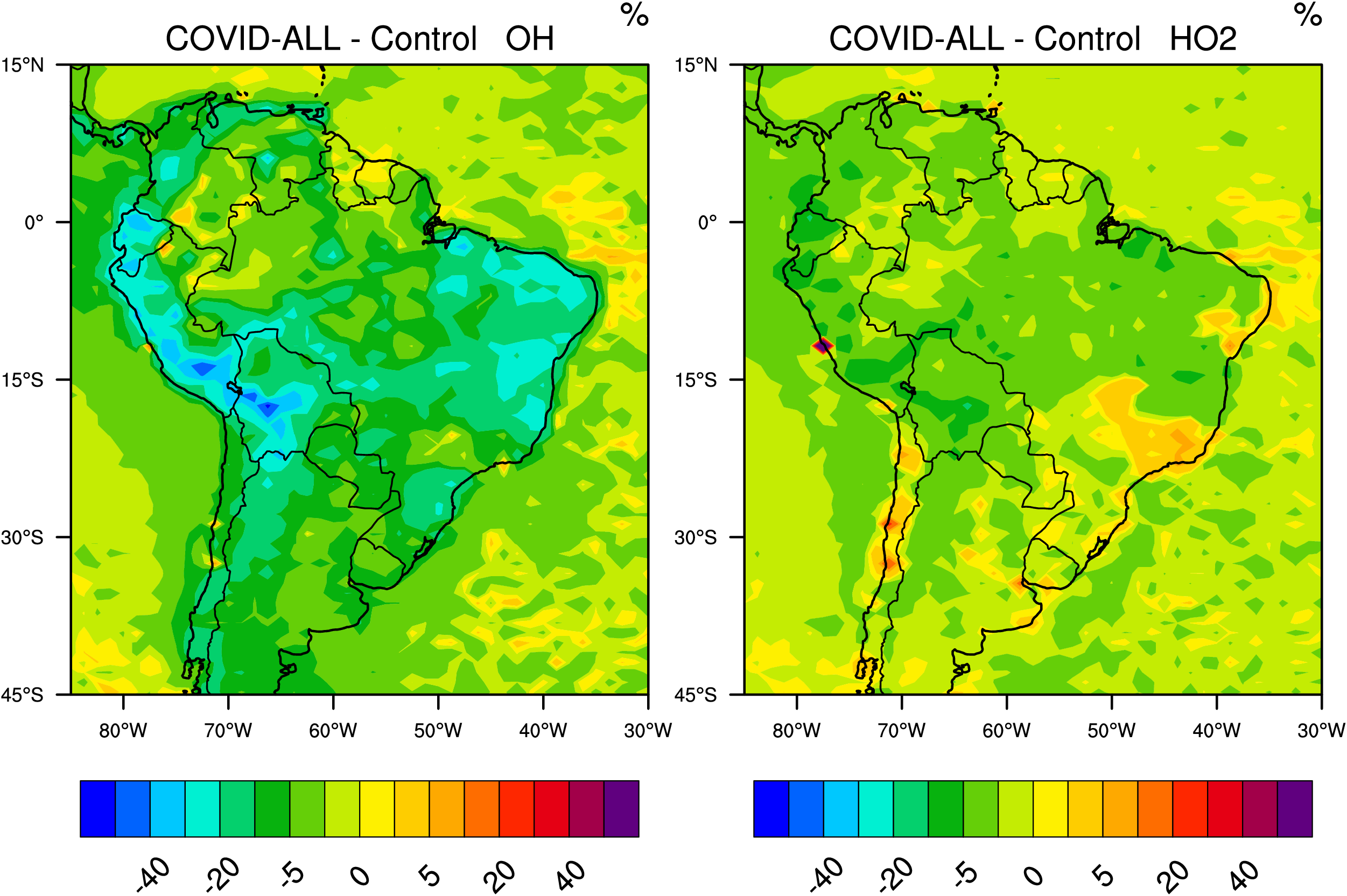
**

**Figure S13.** Percentage change in the surface concentration of several chemical species across South America in response to adjusted emissions of primary pollutants during the COVID-19 period of 15 March-14 April 2020.

**References**

Barré, J., Petetin, H., Colette, A., Guevara, M., Peuch, V.-H., … Kouznetsov, V. (2020) Estimated lockdown induced European NO_2_ changes, *Atmospheric Chemistry and Physics Discussions*, https://doi.org/10.5194/acp-2020-995

Bauwens, M., Compernolle, S., Stavrakou, T., Müller, J.-F., van Gent, J., Eskes, H., … Zehner, C. (2020). Impact of coronavirus outbreak on NO_2_ pollution assessed using TROPOMI and OMI observations. *Geophysical Research Letters*, *47*, e2020GL087978. <https://doi.org/10.1029/2020GL087978>

Crippa, M., Guizzardi, D., Muntean, M., Schaaf, E., Dentener, F., van Aardenne, … Janssens-Maenhout, G. (2018) Gridded emissions of air pollutants for the period 1970–2012 within EDGAR v4.3.2, *Earth Syst. Sci. Data,* *10*, 1987–2013, https://doi.org/10.5194/essd-10-1987-2018

Danabasoglu, G., Lamarque, J.‐F., Bacmeister, J., Bailey, D. A., DuVivier, A. K., Edwards, J., et al. (2020). The Community Earth System Model Version 2 (CESM2). *Journal of Advances in Modeling Earth Systems*, 12, e2019MS001916. <https://doi.org/10.1029/2019MS001916>.

Darmenov, A., & da Silva, A.M. (2014). The QuickFire Emissions Dataset (QFED)—Documentation of versions 2.1, 2.2 and 2.4, NASATM-2013-104606, vol. 35, 183 pp. (data available at: https://portal.nccs.nasa.gov/datashare/iesa/aerosol/emissions/QFED/v2.4r6/, last access: 17 January 2020).

Doumbia, T., Granier, C., Elguindi, N., Bouarar, I., Darras, S., Brasseur, G., … Wang, T. (2021) Changes in global air pollutant emissions during the COVID-19 pandemic: a dataset for atmospheric chemistry modeling, submitted to *Earth. Sys. Sci. Data*

Elguindi, N., Granier, C., Stavrakou, T., Darras, S., Bauwens, M., Cao, … Zheng, B. (2020) Intercomparison of Magnitudes and Trends in Anthropogenic Surface Emissions from Bottom-Up Inventories, Top-Down Estimates, and Emission Scenarios. *Earth's Future*, 8: e2020EF001520. doi:10.1029/2020EF001520

Emmons, L. K., Schwantes, R. H., Orlando, J. J., Tyndall, G., Kinnison, D., Lamarque, J.-F., … Pétron, G. (2020). The Chemistry Mechanism in the Community Earth System Model version 2 (CESM2). *Journal of Advances in Modeling Earth Systems*, *12*, e2019MS001882. <https://doi.org/10.1029/2019MS001882>

Gaubert, B., et al. (2016), Toward a chemical reanalysis in a coupled chemistry‐climate model: An evaluation of MOPITT CO assimilation and its impact on tropospheric composition, *J. Geophys. Res. Atmos.*, 121, 7310– 7343, doi:[10.1002/2016JD024863](https://doi.org/10.1002/2016JD024863).

Gaubert, B., Emmons, L.K., Reader, K., Tilmes, S., Miyazaki, K., … Ren, X. (2020). Correcting model biases of CO in East Asia: impact on oxidant distributions during KORUS-AQ. *Atmos. Chem. Phys. Discuss*., <https://doi.org/10.5194/acp-2020-599>.

Gettelman, A., Mills, M. J., Kinnison, D. E., Garcia, R. R., Smith, A. K., Marsh, D. R., … Randel, W.J. (2019). The whole atmosphere community climate model version 6 (WACCM6). *Journal of Geophysical Research: Atmospheres,* *124*, <https://doi.org/10.1029/2019JD030943>.

Goldberg, D. L., Anenberg, S. C., Griffin, D., McLinden, C. A., Lu, Z. & Streets, D. G. (2020). Disentangling the Impact of the COVID‐19 Lockdowns on Urban NO2 From Natural Variability. ***Geophysical Research Letters,*** *47*, https://doi.org/10.1029/2020GL089269.

Granier, C., Darras, S., Denier van der Gon, H., Doubalova, J., Elguindi, N., Galle, B., … Sindelarova, K. (2019). The Copernicus Atmosphere Monitoring Service global and regional emissions, Copernicus Atmosphere Monitoring Service (CAMS), doi:10.24380/d0bn-kx16

Guenther, A. B., Jiang, X., Heald, C.L., Sakulyanontvittaya, T., Duhl, T., Emmons, L.K. & Wang X. (2012). The Model of Emissions of Gases and Aerosols from Nature version 2.1 (MEGAN2.1): An extended and updated framework for modeling biogenic emissions, *Geosci. Model Dev*., *5*, 1471–1492, doi:10.5194/gmd-5-1471-2012

Hoesly, R. M., Smith, S. J., Feng, L., Klimont, Z., Janssens-Maenhout, G., Pitkanen, … Zhang, Q. (2018). Historical (1750–2014) anthropogenic emissions of reactive gases and aerosols from the Community Emissions Data System (CEDS), *Geosci. Model Dev*., *11*, 369–408, <https://doi.org/10.5194/gmd-11-369-2018>.

Keller, C. A., Evans, M. J., Knowland, K. E., Hasenkopf, C. A., Modekurty, S., Lucchesi, R. A., Oda, T., Franca, B. B., Mandarino, F. C., Díaz Suárez, M. V., Ryan, R. G., Fakes, L. H., and Pawson, S. (2020). Global Impact of COVID-19 Restrictions on the Surface Concentrations of Nitrogen Dioxide and Ozone, Atmos. Chem. Phys. Discuss., https://doi.org/10.5194/acp-2020-685.

Lawrence, D. M., Fisher, R. A., Koven, C. D., Oleson, K. W., Swenson, S. C., Bonan, G., … Zeng, X. (2019). The Community Land Model version 5: Description of new features, benchmarking, and impact of forcing uncertainty. *Journal of Advances in Modeling Earth Systems,* *11*, 4245– 4287. <https://doi.org/10.1029/2018MS001583>

Li, K., Jacob, D. J., Liao, H., Qiu, Y., Shen L., Zhai, S., Bates, K. H., Sulprizio, M. P., Song, S., Lu, X., Zhamg, Q., & Zheng, B. (2021) Ozone pollution in the North China Plain spreading into late-winter haze season, *Proceedings of the National Academy of Sciences,* *118*(10) e2015797118; doi: 10.1073/pnas.2015797118

Liu, X., Ma, P.-L., Wang, H., Tilmes, S., Singh, B., Easter, R. C., Ghan, S. J., & Rasch P.J. (2016). Description and evaluation of a new four-mode version of the Modal Aerosol Module (MAM4) within version 5.3 of the Community Atmosphere Model. *Geoscientific Model Development*, *9* (2), 505–522. <https://doi.org/10.5194/gmd-9-505-2016>

Mills, M. J., Schmidt, A., Easter, R., Solomon, S., Kinnison, D. E., Ghan, S. J., … Gettelman, A. (2016). Global volcanic aerosol properties derived from emissions, 1990–2014, using CESM1c(WACCM), *Journal of Geophysical Research: Atmospheres*, *121*, 2332–2348. <https://doi.org/10.1002/2015JD024290>

Stein, O., Schultz, M. G., Bouarar, I., Clark, H., Huijnen, V., Gaudel, A., ... Clerbaux, C. (2014). On the wintertime low bias of Northern Hemisphere carbon monoxide found in global model simulations. Atmospheric Chemistry and Physics, 14, 9295–9316. https://doi.org/ 10.5194/acp-14-9295-2014.

Tilmes, S., Hodzic, A., Emmons, L. K., Mills, M. J., Gettelman, A., Kinnison, D. E., … Liu, X., (2020). Climate forcing and trends of organic aerosols in the Community Earth System Model (CESM2). *Journal of Advances in Modeling Earth Systems,* *11*, 4323–4351. <https://doi.org/10.1029/2019MS001827>.

Wang, P., Chen, K., Zhu, S., Wang, P. & Zhang, H. (2020). Severe air pollution events not avoided by reduced anthropogenic activities during COVID-19 outbreak, *Resources, Conservation and recycling, 158*, https://doi.org/10.1016/j.resconrec.2020.104814

Zheng, B., Tong, D., Li, M., Liu, F., Hong, C., Geng, G., Li, H., Li, X., Peng, L., Qi, J., Yan, L., Zhang, Y., Zhao, H., Zheng, Y., He, K., and Zhang, Q. (2018) Trends in China's anthropogenic emissions since 2010 as the consequence of clean air actions, Atmos. Chem. Phys., 18, 14095–14111, https://doi.org/10.5194/acp-18-14095-2018.
